# Scaffold RNA Reengineering for a Leakage‐Suppressed CRISPR/Cas12a Cascade System Toward Precise Nucleic Acid Diagnostics

**DOI:** 10.1002/advs.78246

**Published:** 2026-10-22

**Authors:** Xingrong Li, Honghua Hu, Xinli Jiang, Xuhuai Fu, Lin Hu, Qinghua Wu, Jiatong Chai, Diangeng Li, Jinhong Guo, Zhiqiang Li, Yi Li, Yirong Li

**Affiliations:** ^1^ Department of Clinical Laboratory Zhongnan Hospital of Wuhan University Wuhan China; ^2^ Key Laboratory of Clinical Laboratory Diagnostics (Ministry of Education) College of Laboratory Medicine Chongqing Medical University Chongqing China; ^3^ Department of Laboratory Medicine and Sichuan Provincial Key Laboratory for Human Disease Gene Study Sichuan Provincial People's Hospital University of Electronic Science and Technology of China Chengdu China; ^4^ Department of Academic Research Beijing Ditan Hospital Capital Medical University National Center for Infectious Diseases (Beijing) Beijing China; ^5^ Brain Glioma Center Zhongnan Hospital of Wuhan University Wuhan University Wuhan China; ^6^ Western Institute of Digital‐Intelligent Medicine Chongqing China; ^7^ Hubei Provincial Clinical Medical Research Center for Molecular Diagnostics Wuhan China; ^8^ Wuhan Research Center for Infectious Diseases and Cancer Chinese Academy of Medical Sciences Wuhan China; ^9^ Hubei Engineering Center for Infectious Disease Prevention Control and Treatment Wuhan China

**Keywords:** amplification‐free, CRISPR/Cas12a, engineering modification, nucleic acid detection, steric hindrance

## Abstract

Amplification‐free CRISPR detection has attracted significant attention for its capacity to circumvent the intricacies of nucleic acid amplification. Yet, the persistent challenge of high background signals remains a critical bottleneck, motivating intensive efforts toward background suppression strategies. Here, we present a 3’‐end DNA extension of scaffold RNA (3’D‐SR) conformational switch to drive the cascade‐activatable and leakage‐suppressed Cas12a system, a mechanism we denote as CALCas. By engineering the crRNA of Cas12a system and introducing steric hindrance as an activation barrier, we reshape crRNA into a “threshold‐controlled” smart molecular switch, enabling Cas12a to shift from “inhibition” to “precise activation”. Unlike other Cas cascade reaction generally suffer from internal self‐activation that leads to uncontrolled background increase, this newly developed framework, leveraging a DNA‐mediated allosteric regulation mechanism and a leakage‐suppressed design based on inherent steric hindrance, effectively solves the dilemma between signal enlargement and minor background variation. Through rational design, this cascaded CRISPR system achieves femtomolar sensitivity (over 10,000‐fold higher than single Cas12a system), enables rapid HPV16/18 detection, and reduces assay time to under 30 min. This engineered crRNA approach unlocks a new pathway to reprogram Cas12a's enzymatic activities with a stabilized benchmark signal that can be critical in precise quantitative nucleic acid detection.

## Graphical abstract

Schematic illustration of the CALCas system, in which a conformational switch in the 3' terminal DNA extension of scaffold RNA (3'D‐SR) drives cascade‐activatable Cas12a cleavage while suppressing leakage.

## 1 Introduction

As a prominent next‐generation diagnostic technology, the CRISPR/Cas (Clustered Regularly Interspaced Short Palindromic Repeats/CRISPR‐associated proteins) system exhibits a powerful potential for biosensing applications [1, 2]. Particularly, Cas12a has been employed for nucleic acid detection based on the discovery of the indiscriminate cleavage of non‐target nucleic acids (termed *trans*‐cleavage activity) [3, 4, 5]. Nevertheless, the intrinsic *trans*‐cleavage activity confines Cas12a systems to pM‐nM detection sensitivity, hampering the detection of low‐abundance targets. Although subsequent systems (DETECTR [6], SHERLOCK [7], et al.) achieved a higher sensitivity, they rely on pre‐amplification steps like RPA (recombinase polymerase amplification), LAMP (loop‐mediated isothermal amplification), RCA (rolling circle amplification), or EXPAR (exponential amplification reaction), which could elevate costs, introduce aerosol contamination, and compromise their utility in point‐of‐care or resource‐limited environments [8, 9, 10, 11, 12]. Subsequently, the integration of the CRISPR/Cas system with enzyme‐free amplification methods, such as CHA (catalytic hairpin assembly) [13, 14] or HCR (hybridization chain reaction) [15, 16], enables efficient signal cycling amplification by employing the products of enzyme‐free circuits as Cas recognition targets. However, the insufficient thermodynamic stability of such systems may trigger nonspecific Cas cleavage, elevating background noise and limiting their practical applicability.

To move beyond these constraints, recent studies capitalize on exploiting the endogenous high‐turnover *trans‐*cleavage activity of CRISPR/Cas systems as programmable circuitry for constructing nucleic acid autocatalytic systems. The fundamental objective is to remove steric or sequence‐level repression using *trans*‐cleavage of Cas, consequently enabling the liberation or reformation of active crRNA or activators to construct a positive feedback loop for potent signal amplification. Nie and colleagues [17] pioneered a double‐bulge caged switchable gRNA (scgRNA) Cas12a system, wherein *trans*‐cleavage‐mediated removal of the inhibitory strand liberates the gRNA, which subsequently assembles into active RNP complexes, thereby establishing a positive‐feedback loop termed CONAN (CRISPR‐Cas‐only amplification network). Leveraging the high site accessibility of the bulge structure, which provides rapid kinetics and efficient cleavage for positive‐feedback amplification, subsequent studies have developed V‑probe [18], CHA‑coupled [19], split‑crRNA topologies [20], or double‐bulge [21, 22] through varied spatial locking/unlocking mechanisms, collectively optimizing the activation efficiency and flexibility of the feedback loop and thus further improving overall detection performance. Building on this concept, we also established the AutoCAR (Autocatalytic Cas12a Circular DNA Amplification Reaction) system in the previous study [23]. This circular‐DNA strategy not only enables autocatalytic Cas12a activation through topological caging and Cas‐triggered linearization, but has also been broadly extended to diverse diagnostic scenarios, accommodating various target types and readout modalities [24, 25]. In a related approach, researchers conceal the activator within the stem duplex. Then, Cas‐mediated cleavage in the loop region opens the hairpin, releasing the intact activator [26, 27]. Similarly, precise chemical modifications such as LNA and the spatial arrangement of phosphorothioate (PS) modifications can also regulate the transition of Cas12a from a dormant state to an active state, thereby establishing CRISPR‑Cas autocatalysis amplification, which effectively overcomes the degradation susceptibility of traditional probe designs [28, 29]. Additionally, by modularly cascading Cas proteins (e.g., Cas13a/Cas14a [30], Cas13a/Cas12a [31, 32], Cas12a/Cas13a [33]) into tandem signal‐amplifying units, a Cas‐driven autocatalytic system is established. Whereas carefully engineered circular, hairpin, or self‐complementary structures may still suffer from thermodynamic instability under reaction conditions. Moreover, perturbations to secondary/tertiary folding, including changes in ionic strength, temperature variations, or off‐target nucleic acid interactions, may impair probe functionality and inadvertently initiate signal cycling, yielding false‐positive outputs. The multi‐enzyme cooperative strategy faces practical engineering hurdles, including enzyme kinetics matching, buffer compatibility, and system robustness. Thus, with complex reaction matrices or longer reaction times, unexpected structural changes, such as nonspecific unfolding or degradation, can potentially lead to an increased or fluctuated baseline signal.

Recognized as an intrinsic functional element with high programmability and structural flexibility, crRNA offers significant promise for pioneering applications [34, 35, 36]. Through systematic exploration, we present an innovative strategy that integrates conformational switching and amplification capabilities into the engineered 3’D‐SR (DNA extension at the 3’ end of scaffold RNA) framework, creating a multifunctional “smart” CALCas (the cascade‐activatable and leakage‐suppressed Cas12a) system. In this design, upon target recognition, the upstream Cas12a complex is activated and cleaves the 3’D‐SR. This cleavage spatially relieves the inhibitory constraints imposed by the 3’D‐SR and promotes the orderly assembly of the resulting fragments with the downstream Cas12a complex, thereby establishing a molecular circuit for signal transduction and coordinated cascade amplification. Through the steric hindrance of a 3’D‐SR lock, a physical barrier is imposed directly at the signal origin, creating a “source‐blocking” mechanism for precise unlocking. Beyond leakage suppression, this design integrates a commercially ready‐to‐use strategy to build a modularized amplification module, fundamentally redefining crRNA as a multifaceted molecular platform rather than a mere targeting scaffold, thus providing an essential but simple principle for effective Cas‐associated molecular switch design with emphasis on stable and reproducible background control in a single enzyme‐based diagnostic scheme.

## 2 Results and Discussion

### 2.1 Design Strategy of the CALCas System

The crRNA in the CRISPR/Cas12a system generally comprises two critical sections: a protein‐binding scaffold region and a target‐recognition and hybridization spacer region, and can be artificially split into scaffold RNA and spacer RNA segments. It has been reported that the split crRNA has a similar potential to standard crRNA for the activation of Cas12a, which relies on the formation of precise complementary conformations between crRNA and Cas12a [37, 38, 39]. On this basis, we engineered the scaffold RNA segment by appending a 3’ DNA extension to create a sterically hindered variant (3’D‐SR). In the absence of the target, this 3’D‐SR may serve as a safety lock that blocks Cas12a activation, thereby suppressing nonspecific fluorescence background. Upon recognition of the target sequence by the crRNA, the activated upstream Cas12a cleaves 3’D‐SR to release the scaffold RNA, which then serves as a bridging molecule that cooperates with the spacer RNA, substrate DNA, and downstream Cas12a to establish the CALCas (the cascade‐activatable and leakage‐suppressed Cas12a) system, ultimately producing a remarkably enhanced fluorescence signal (Scheme 1).

**SCHEME 1:**
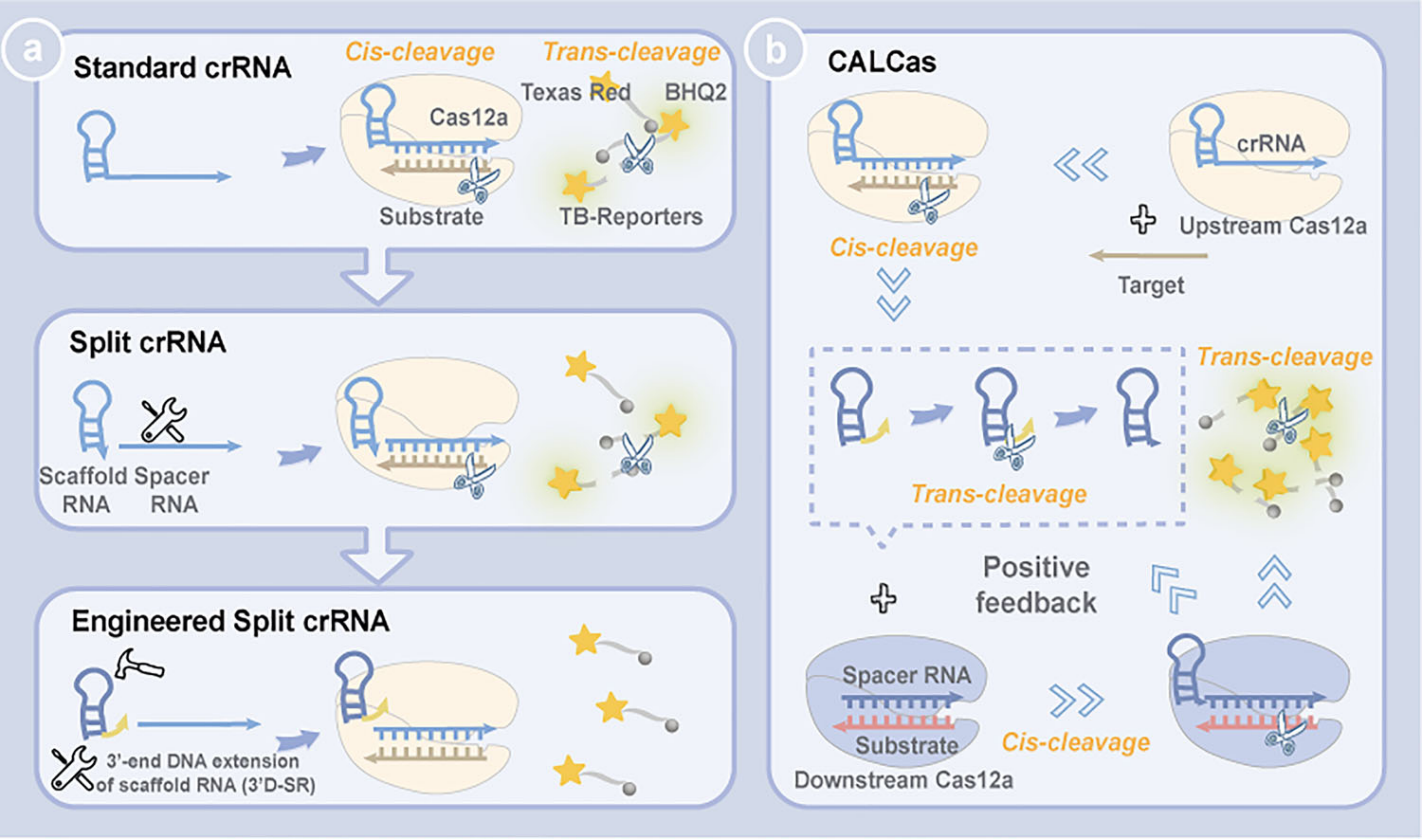
Principle of the CALCas system. (a) Diagram illustrating the binding of three distinct crRNAs (Standard crRNA, Split crRNA, and Engineered Split crRNA) to Cas12a and their respective cleavage activities. (b) Schematic of the CALCas system.

### 2.2 Evaluation of Engineered crRNA Variants for Cas12a Inhibition

The cleavage activity of Cas12a was reported to be inhibited by biotin modifications on the 3’ ends of the scaffold RNA [40]. For confirmation, the split crRNA and its variants with 5’ or 3’ terminal biotin modifications were synthesized, and a fluorescence verification assay was employed to test their cleavage activity using TB (Texas Red/BHQ2 dual‐labeled) reporter probes (Figure 1a). Among all tested modification patterns, only the 3’ end biotin modification in the scaffold RNA strongly attenuated the *trans* cleavage activity by 95% to 75%; 5’ terminal modifications on the spacer RNA can cause a moderate inhibition of approximately 45% to 65%, whereas other modifications showed minimal effects (Figure 1b and Figure S1).

**FIGURE 1:**
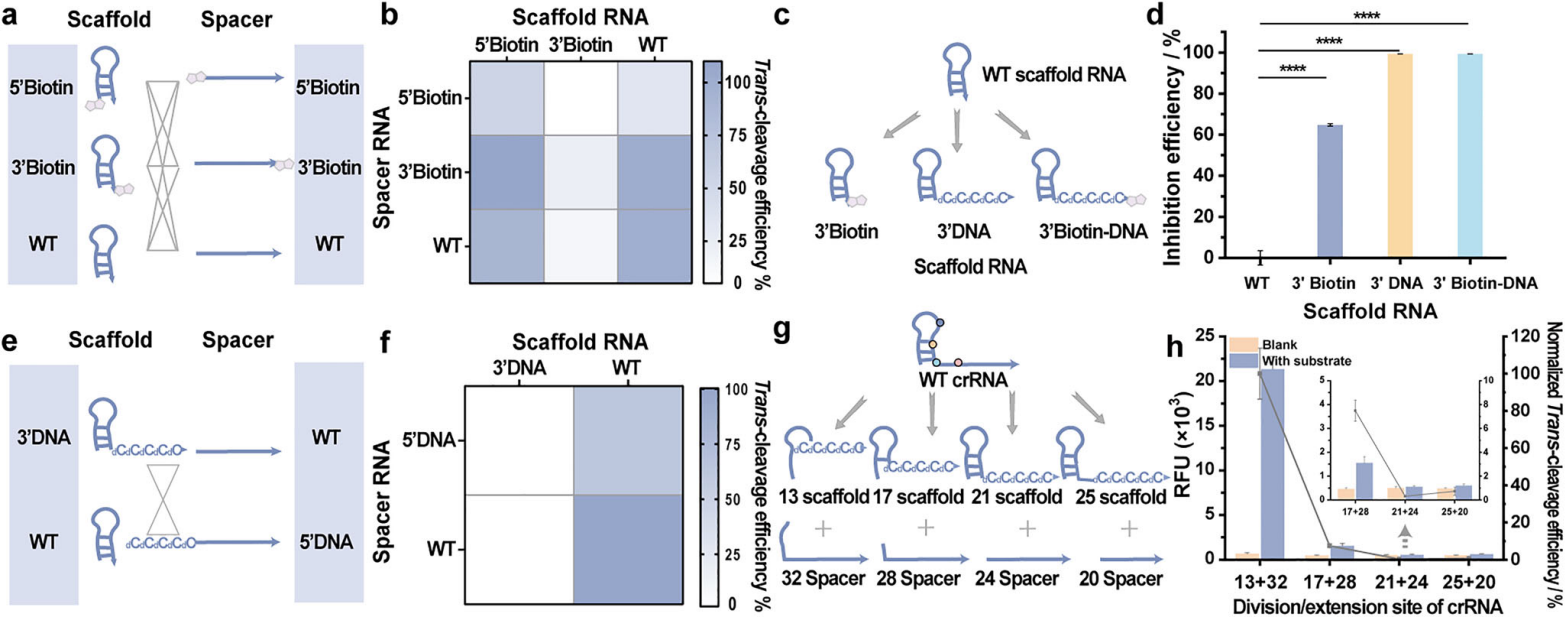
Evaluation of engineered crRNA variants for Cas12a inhibition. (a,b) Schematic illustration of the scaffold RNA and spacer RNA with or without 5’/3’ end biotin modifications, and the *trans*‐cleavage efficiency of Cas12a under these modifications (WT split crRNA's *trans*‐cleavage efficiency was defined as 100%). (c,d) Schematic illustration of the scaffold RNA modified with biotin, DNA, or biotin‐DNA conjugate at the 3’ end, and the inhibitory efficacy of these modifications on Cas12a activity (The inhibition efficiency of the 3’ Biotin‐DNA scaffold RNA was set to 100%). (e,f) Schematic illustration of the 3’ DNA extension of scaffold RNA and 5’ DNA extension of spacer RNA, and the *trans*‐cleavage efficiency of Cas12a following these modifications (WT split crRNA's *trans*‐cleavage efficiency was defined as 100%). (g,h) Structural illustrations of split crRNA variants with DNA extension, and the *trans*‐cleavage activity of Cas12a in response to these variations (The *trans*‐cleavage efficiency of the “13+32” group was set to 100%). Data are expressed as mean ± SD (*n* = 3). In Figure 1d, the levels of significance relative to the WT group were assessed by one‑way ANOVA with Dunnett's post‑hoc test and are denoted as ^****^, *p* < 0.0001.

It has also been reported that locally introducing DNA chimeric fragments is a successful method for disrupting the activity of Cas12a [41]. Thus, we wondered whether DNA extension at the 3’ end of scaffold RNA could also produce the inhibitory effect. Three modification configurations (biotin attachment exclusively, DNA sequence solely, and a combination modification featuring both biotin and DNA) were synthesized to evaluate their inhibitory effect, respectively (Figure 1c). Compared to the WT scaffold RNA, the cleavage activity of Cas12a was inhibited to varying levels in all modified scaffold RNAs. Notably, modification with DNA alone was sufficient to completely inhibit the cleavage activity of Cas12a (Figure 1d and Figure S2). A plausible explanation is that the DNA extension on the scaffold RNA creates a sustained steric barrier, thereby effectively inhibiting the substrate DNA binding to the catalytic site of Cas12a. In contrast, biotin modification, owing to its small molecular weight and flexible conformation, only achieves a localized and transient spatial hindrance, resulting in substantially weaker inhibitory efficacy.

To further elucidate whether the inhibitory effect of DNA extension on Cas12a activity operates through a universal mechanism, we designed and synthesized spacer RNA with 5’ end DNA extension and performed comparative analyses with previously validated scaffold RNA with 3’ DNA extension (Figure 1e). It was found that DNA extension on the 5’ end of the spacer RNA led to only partial inhibition of Cas12a's cleavage activity (Figure 1f and Figure S3). We suspect that this level of modification is an “affinity‐weakening” inhibition (impaired hybridization), which is insufficient to fully compromise the activation of Cas12a.

Since 3’ DNA extension on scaffold RNA completely inhibits the cleavage activity of Cas12a, we wonder whether identical 3’ end DNA extension on different split variants causes the same inhibitory effect. Therefore, a break was introduced into the scaffold, spacer, or the scaffold‐spacer junction, yielding four split variants: “13+32”, “17+28”, “21+24”, and “25+20” (Figure 1g). The numbers denote the nucleotide lengths of the scaffold RNA and spacer RNA, respectively. Among them, “21+24” serves as the standard reference configuration, with a 21 nt scaffold segment and a 24 nt spacer segment. It was found that the DNA extension of the split variants, except for “13+32,” effectively suppressed Cas12a activity. (Figure 1h and Figure S4). The results confirm that the allostery of Cas12a under engineered RNA is closely associated with the interface between the scaffold RNA and spacer RNA. In the “17+28”, “21+24”, and “25+20” split crRNA variants, the introduced DNA extension creates a steric hindrance that may stably shield the active site of Cas12a. In contrast, the “13+32” split crRNA variant couldn't inhibit Cas12a's activity, likely because its division site is located outside the key recognition region of Cas12a. Notably, a basal level of background noise was detected throughout all segmentation sites in the absence of substrate. By conducting a comprehensive evaluation, “21+24” was identified as the optimal division and extension site, which facilitated the combined enhancement of inhibitory efficacy and conformational stability. Furthermore, a set of crRNA variants combining DNA modifications with engineered conformational changes were also assessed, with details provided in Figure S5. Among these, the 3’D‐SR, the split crRNA with a 3’ DNA extension on the scaffold RNA, remained the most potent inhibitor, surpassing all other engineering strategies tested.

### 2.3 Exploration of the Molecular Mechanism Underlying the Inhibition of 3’D‐SR

Prior work found that 3’D‐SR strongly suppressed Cas12a's enzymatic activity. To further elucidate the underlying inhibitory mechanism, we used fluorescence resonance energy transfer (FRET) to conduct a multi‐faceted analysis of the dynamic conformational changes of 3’D‐SR at the molecular level. First, we attached the donor fluorophore Cy5 to the 3’ terminus of the WT spacer RNA and an acceptor fluorophore Cy3 to the 5’ terminus of the WT substrate, and continuously monitored the time‐dependent changes in the fluorescence intensities of the FRET, Cy3, and Cy5 channels. As shown in Figure 2a,b, the Cy3‐labeled WT substrate was introduced at the start of the reaction, resulting in a high fluorescence signal exclusively in the Cy3 channel. Subsequently, the Cy5‐labeled WT spacer was added at 10 min, bringing the donor (Cy3) and acceptor (Cy5) into proximity and thereby leading to a significant increase in the FRET signal accompanied by a rapid decrease in the Cy3 channel and a slight increase in the Cy5 channel. Upon addition of WT scaffold RNA and Cas12a at 30 min, Cas12a was activated and subsequently cleaved the substrate, resulting in the physical separation of Cy5 and Cy3. This decoupling elicited a prompt diminution of the FRET signal, after which the signal gradually reverted to its initial baseline level over a period of approximately 20 min. In parallel, the respective fluorescence emissions of Cy5 and Cy3 exhibited sustained increases until reaching a terminal plateau. In contrast, the addition of 3’D‐SR and Cas12a behaved in an opposite manner under the same conditions. The FRET signal maintained a robust high plateau over the entire subsequent monitoring period, while the Cy5 and Cy3 channels remained essentially flat. Quantitatively, the FRET efficiency of the WT scaffold RNA decreased by approximately 80%, while that of 3'D‐SR decreased by approximately 20%. This compelling evidence strongly demonstrates that the 3’D‐SR effectively suppresses *cis*‐cleavage of Cas12a. Besides, this phenomenon was also validated by fluorescence assays and gel electrophoresis analysis (Figures 2c,d and S6–S8).

**FIGURE 2:**
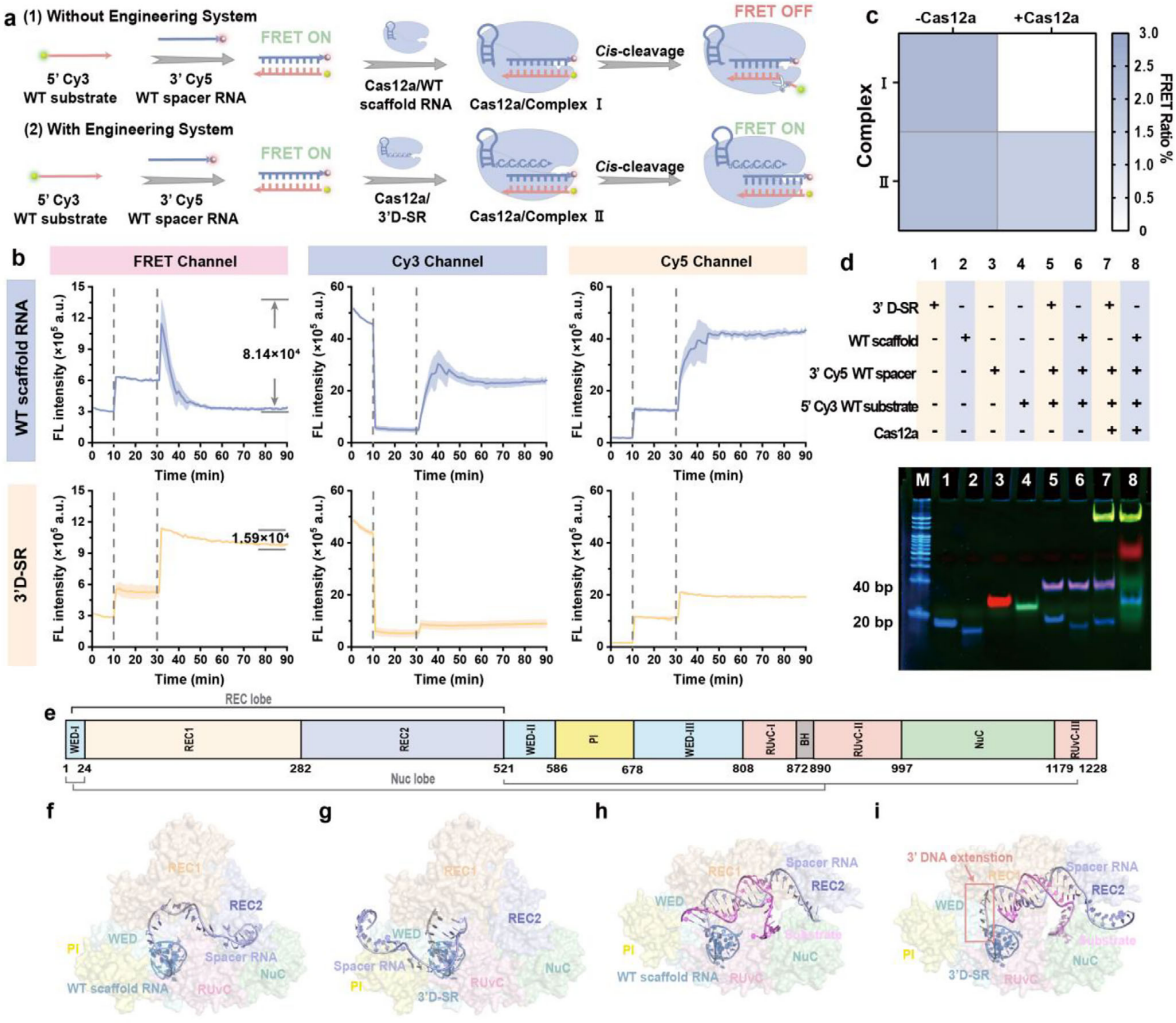
Potential mechanisms of the inhibition of Cas12a activity by 3’D‐SR. (a–c) Schematic representation of the FRET‐based assay and the corresponding real‐time fluorescence kinetic traces. (d) PAGE assay. Lane M: 20 bp marker; lane 1:3’D‐SR; lane 2: WT scaffold RNA; lane 3: 3’ Cy5 WT spacer RNA; lane 4: 5’ Cy3 WT substrate DNA; lane 5: 3’D‐SR, 3’ Cy5 WT spacer RNA and 5’ Cy3 WT substrate DNA, called Complex II; lane 6: WT scaffold RNA, 3’ Cy5 WT spacer RNA and 5’ Cy3 WT substrate DNA, called Complex I; lane 7: Complex II with Cas12a; lane 8: Complex I with Cas12a. (e) Domain organization of Cas12a. (f‐i) Molecular docking simulation results of multiple complex models. Data are expressed as mean ± SD (*n* = 3).

To further directly explore the molecular mechanisms, we performed molecular docking and molecular dynamics simulations, and constructed multiple complex models containing Split RNP (Cas12a with WT spacer RNA and WT scaffold RNA), engSplit RNP (Cas12a with WT spacer RNA and 3’D‐SR), and their respective ssDNA substrate‐bound counterparts (Split RNP/DNA complex and engSplit RNP/DNA complex). In the absence of ssDNA substrate, both the Split RNP and the engSplit RNP adopt a compact closed conformation, wherein the RuvC catalytic domain is shielded by surrounding residues and structural loops, thereby suppressing nuclease activity and maintaining the complexes in a catalytically inert state (Figure 2f,g). Upon addition of the ssDNA substrate to the Split RNP, the WT spacer RNA can effectively base‐pair with the ssDNA substrate to form an RNA‐DNA heteroduplex. The formation of this heteroduplex induces a pronounced conformational rearrangement of Cas12a, which repositions the target ssDNA into the RuvC catalytic pocket and converts the RuvC nuclease active center from a resting state to an activated state, thereby initiating the *cis*‐cleavage of the ssDNA substrate (Figure 2h and Figure S9a,d). In contrast, in the engSplit RNP/DNA complex, the additional DNA of scaffold RNA causes local steric hindrance by occupying the limited space within Cas12a, inducing conformational perturbation of the RNA‐DNA heteroduplex formed between the spacer RNA and target DNA. As a result, the ssDNA deviates from its normal binding position and moves away from the catalytic pocket, ultimately inhibiting Cas12a activity (Figure 2i and Figure S9c,f). Then, we compared the root mean square deviation (RMSD) and root mean square fluctuation (RMSF) between the Split RNP/DNA and engSplit RNP/DNA complex. As shown in Figure S9g–l, the 5 nt DNA extension has a negligible impact on the overall conformational stability or residue flexibility of the Cas12a. Thus, this inhibitory effect is primarily due to the steric hindrance effect generated by the DNA extending from the scaffold RNA. Consistent with structural predictions, the calculated ΔG (Change in Gibbs Free Energy) values of the engSplit RNP/DNA complex are elevated to near‐zero levels compared with the Split RNP/DNA complex, indicating that 3’D‐SR strongly suppressed Cas12a's enzymatic activity (Figure S9m–o).

### 2.4 Inhibition of Cas12a by 3’D‐SR: influencing Factors and Applicability

To assess how different DNA nucleotide bases affect 3’D‐SR‐mediated inhibition of Cas12a, we generated four 3’D‐SR variants composed solely of A, T, C, or G. As shown in Figure 3a,b, all extension DNA base types effectively inhibited Cas12a. We also substituted the first base at the 5’ end of the spacer RNA, in addition to changing the DNA base composition of 3’D‐SR, to exclude any potential interaction between this position and the 3’D‐SR terminal base that might affect inhibition. These changes did not significantly alter the extent of Cas12a inhibition, indicating that 3’D‐SR inhibition is broadly effective (Figure 3a,b and Figure S10).

**FIGURE 3:**
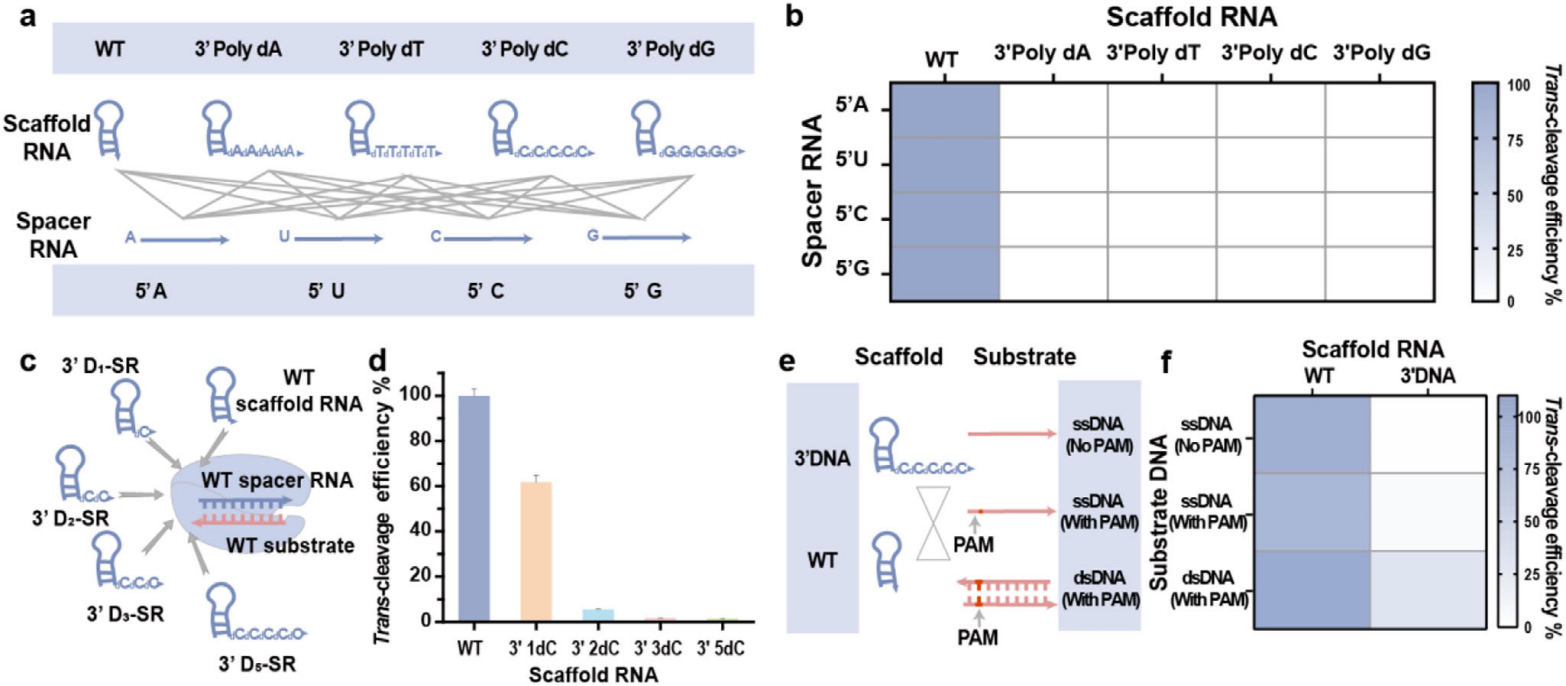
Exploring the factors influencing 3′D‐SR‐mediated inhibition of Cas12a activity. (a,b) Schematic illustration of the 3’D‐SR with different DNA bases (A/T/G/C) and the impact of these extension on Cas12a‐mediated *trans*‐cleavage efficiency (WT split crRNA's cleavage efficiency was defined as 100%). (c,d) Schematic illustration of the scaffold RNA with different lengths of DNA and the *trans*‐cleavage efficiency of Cas12a activity under these lengths (WT split crRNA's *trans*‐cleavage efficiency was defined as 100%). (e,f) Schematic illustration of the 3’D‐SR with different structures of substrate, and the *trans*‐cleavage efficiency of Cas12a under different structures of substrate (the *trans*‐cleavage efficiency of dsDNA with PAM site was defined as 100%). The red highlights the PAM site. Data are expressed as mean ± SD (*n* = 3).

Then, we proceeded to explore the impact of different DNA lengths of 3’D‐SR on the activity of Cas12a (Figure 3c,d and Figure S11). Experimental results showed that signal inhibition efficiency is positively correlated with the DNA length of 3’D‐SR. Single‐base extension resulted in partial inhibition (40%), whereas double‐base extension achieved near‐complete inhibition (95%). Once the length of DNA reached three or more bases, the inhibition efficiency became saturated, resulting in total signal blockage. The molecular docking results further corroborated this finding. Compared with the five base DNA extensions, the two base DNA extensions induced less local steric hindrance and caused only minor conformational perturbation of the RNA‐DNA heteroduplex. However, although the substrate underwent a slight displacement, it was largely displaced away from the catalytic center, leading to a marked reduction in Cas12a cleavage activity and leaving only minimal residual activity (Figure S9).

Given that the catalytic efficiency of Cas12a is highly influenced by the structures of its corresponding nucleic acid substrate, we thoroughly investigated the repressive activity of Cas12a against both ssDNA substrates containing either a canonical PAM (TTTN) or no PAM, and dsDNA containing a PAM (Figure 3e,f and Figure S12). In comparison to the WT scaffold RNA, the 3’D‐SR group exhibited lower cleavage activity on both ssDNA and dsDNA. Among these, a graded reduction in inhibition was observed: from the PAM‐free ssDNA (strongest) to the PAM‐containing ssDNA, to the PAM‐containing dsDNA (weakest). This differential behavior may arise from the fact that high‐affinity PAM binding partially alleviates the allosteric inhibitory barrier, causing Cas12a to transition into a metastable state that facilitates *trans*‐cleavage of the TB reporter.

To elucidate the generality of this suppression strategy, various spacer sequences were employed (Figure S13). The results showed that the 3’D‐SR yielded potent inhibition regardless of spacer identity, highlighting its broad applicability across diverse spacer sequences.

### 2.5 Conformation‐Gated Positive Feedback Cascade Amplification: CALCas System

The scaffold RNA functions as an essential and non‐redundant element of a molecular logic gate for the split Cas12a system, as definitively demonstrated by the signal disappearance following experimental deletion of this component (Figure 4a,b). Given that the engineered scaffold RNA (3’D‐SR) with a precisely controllable inhibitory capacity renders it an ideal mediator for orchestrating signal transduction within synthetic positive feedback networks, we established a specialized experimental framework consisting of three well‐defined sequential steps: (i) the initial inhibition stage, wherein the 3’D‐SR locks the system into an “off” state to minimize background; (ii) the controlled release stage, triggered by the specific target, which relieves the steric hindrance of 3’D‐SR; and (iii) the amplification stage, the liberated scaffold RNA propagate the signal onward, culminating in the activation of downstream cascades.

**FIGURE 4:**
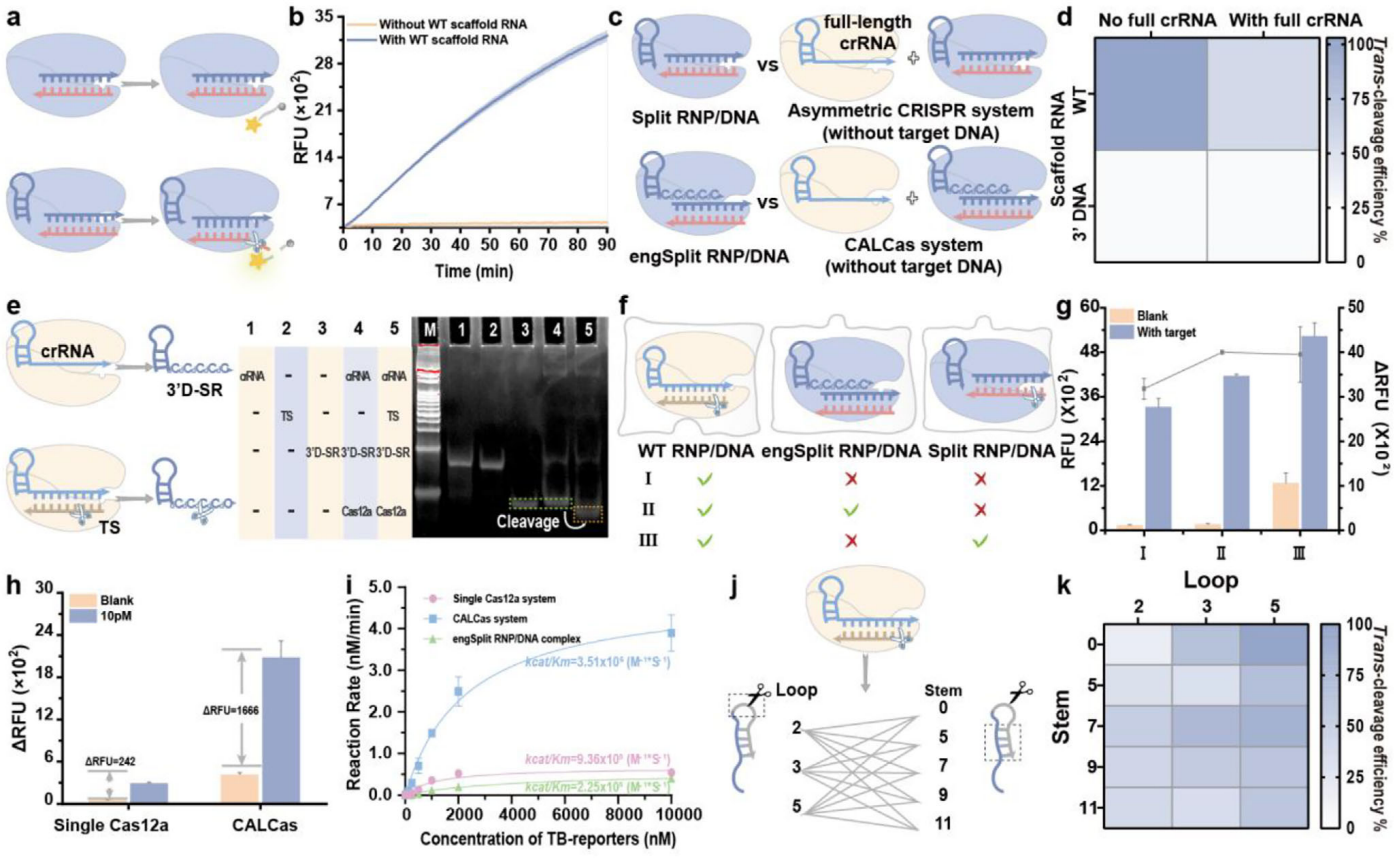
Construction of CALCas System. (a) Schematic diagram of the scaffold RNA‐mediated Cas12a system. (b) The real‐time fluorescence shown in the above Figure 4a. (c) Comparison of the inhibitory performance of the CALCas system and the Asymmetric CRISPR system. (d) The *trans*‐cleavage efficiency of Cas12a in Figure 4c (the *trans‐*cleavage efficiency of the Split RNP/DNA complex was defined as 100%). (e) PAGE validation of Cas12a‐mediated cleavage of 3’D‐SR. Lane M: 20 bp marker; lane 1: crRNA; lane 2: TS; lane 3: 3’D‐SR; lane 4: Cas12a, crRNA with 3’D‐SR; lane 5: Cas12a, crRNA, target with 3’D‐SR. (f) Schematic diagram of the single Cas12a system (I), the CALCas system (II), and the Asymmetric CRISPR assay (III). (g) ΔRFU (The change in RFU) in Figure 4f. (h) Comparison of the ΔRFU of the single Cas12a and CALCas system. (i) Curves of reaction velocity versus substrate concentration for different Cas12a systems. (j,k) Schematic illustration of the scaffold RNA with different loops and stems (blue represents RNA, and gray represents DNA), and the corresponding *trans‐*cleavage efficiency of Cas12a. (3’D_(5,0)_‐SR's *trans*‐cleavage efficiency was defined as 100%). Data are expressed as mean ± SD (*n* = 3).

To assess the inhibitory performance of the CALCas system, we compared it with the Asymmetric CRISPR assay [42], in which high‐affinity full‐length crRNA competitively inhibits split crRNA‐Cas12a binding. Unlike the reported case, our system displays superior inhibition performance in the absence of target DNA, irrespective of the presence of full‐length crRNA. The cleavage activity of the CALCas system is further suppressed to nearly 40% of the Asymmetric CRISPR assay, indicating that the inhibitory mechanism is potentiated in the CALCas system (Figure 4c,d and Figure S14a). To rule out nonspecific activation, we performed a control experiment verifying that the spacer RNA‐substrate DNA hybrid assembled with the full‐length crRNA does not trigger Cas12a cleavage without target DNA (Figure S14b,c). Consistently, the fluorescence intensity showed no significant increase with rising 3’D‐SR concentration, confirming that the background signal from the system is negligible (Figure S14d–f).

Thereafter, pre‐activated Cas12a was utilized as the “molecular scissors” for the validation of the system's release capacity. The 3’D‐SR were separately incubated with pre‐activated and non‐activated Cas12a, followed by analysis via gel electrophoresis. As shown in Figure 4e, compared with the untreated 3’D‐SR (Lane 3), there was no obvious change in the band position of the unactivated Cas12a group (Lane 4). In contrast, a band with a faster migration rate appeared in the pre‐activated Cas12a group (Lane 5). These results preliminarily confirm that the activated Cas12a can cleave the 3’D‐SR and release scaffold RNA. Afterward, the 3’D‐SR was labeled with biotin and a Cy3 fluorophore at its 5’ and 3’ ends, respectively, which allowed monitoring of fragment release through band migration in gel and fluorescence intensity change. It was also shown that the activated Cas12a can cleave the 3’D‐SR (Figure S15). Furthermore, to verify that the cleavage activity of Cas12a is primarily directed against DNA while exerting only minimal effects on the scaffold RNA backbone, additional gel electrophoresis analysis was performed (Figure S16). It was found that a faster‐migrating band appeared in the reaction products derived from the 3’D‐SR treated with pre‐activated Cas12a (lane 3), which was consistent with the untreated WT scaffold RNA control (lane 4). Meanwhile, the band in lane 6 is unchanged compared to lanes 4 and 5. These results confirmed that the released fragments were DNA rather than RNA, concurrently demonstrating the preserved integrity and functionality of the scaffold RNA, thereby robustly validating system reliability.

Meanwhile, to further determine whether the scaffold RNA liberated from 3’D‐SR retains the capacity to activate downstream Cas12a and complete the signal amplification circuit, we prepared three RNP/DNA complexes: WT RNP/DNA (Cas12a with crRNA and target DNA, also called the single Cas12a system), Split RNP/DNA, and engSplit RNP/DNA. The WT RNP/DNA and Split RNP/DNA complexes represent the previously reported CRISPR/Cas12a systems (the Asymmetric CRISPR assay), whereas the WT RNP/DNA and engSplit RNP/DNA complexes correspond to our CALCas system, in which 3’D‐SR was used to substitute for the split scaffold RNA (Figure 4f). As shown in Figure 4g, the net fluorescence signal intensity in the CALCas system was roughly 800 RFU (Relative Fluorescence Units) higher than that in the single Cas12a system, and was slightly above that in the Asymmetric CRISPR system mentioned in the literature [42] (Figure S17). These results validate that the released scaffold RNA has favorable bioactivity to efficiently mediate the cascade reaction. To further exploit the potential of its signal enhancement capability, we implemented a systematic optimization of the critical reaction parameters, which cover the ratio and concentration of upstream Cas12a/crRNA, the concentration of spacer RNA and substrate, along with the concentration of downstream Cas12a/3’D‐SR (Figure S18). Under the optimized protocol, addition of the same target DNA concentration yielded a markedly enhanced signal from the CALCas system compared to that of the single Cas12a system (Figure 4h and Figure S19). Standard Michaelis‐Menten kinetic analysis also revealed an enhanced *k_cat_/K_m_* in the CALCas system, thereby establishing a robust kinetic foundation for the system's subsequent application (Figure 4i and Figure S20). This result validates that in the CALCas system, the presence of a single target molecule leads to the activation of many additional RNPs throughout the reaction process (Figure S21).

To fully leverage the potential of the positive feedback circuit and improve the activation kinetics of Cas12a, we concentrated on refining the sequence composition, length, and spatial conformation of its core regulatory element‐ the extension DNA of the 3’D‐SR to achieve a leap from optimization to rational design. According to the literature, the *trans*‐cleavage activity of Cas12a primarily relies on the base sequence of its DNA substrate [43]. Thus, we designed four different groups of DNA base extension 3’D‐SR and two fluorogenic reporter substrates, aiming to enhance cleavage dynamics of Cas12a by governing the release rate and amount of the functional scaffold RNA, and the cleavage efficiency of the reporter probe that determines the final signal output intensity (Figure S22). Research data demonstrate that coupling cytosine extension of 3’D‐SR with cytosine‐rich reporter designs greatly improves the effectiveness of the cascade reaction.

The non‐specific *trans*‐cleavage activity of Cas12a would cause hydrolysis at random positions, producing fragment populations characterized by high length heterogeneity and indeterminate termini. Consequently, the length of the extension DNA plays a vital role in determining the initial containment efficiency, governs the properties and release of the functional fragment after cleavage, and ultimately impacts the capability of signal amplification. Thus, taking into account the insights mentioned above and Cas12a's documented affinity for hairpin cleavage [44], we strategically integrated length and secondary structure optimization. A structured set of 3’D‐SR variants was developed, incorporating specific loop sizes (2, 3, 5 nt) and stem lengths (0, 5, 7, 9, 11 bp), followed by comprehensive evaluation of their cleavage efficiency in the loop regions (Figure 4j,k and Figures S23–S25). Combinations that included two loop bases show diminished net signal values relative to other combinations, probably due to weakened enzyme‐substrate binding affinity that results in lower cleavage efficiency. Notably, the 3’D_(2‐loop,0‐stem)_‐SR exhibits an extremely high background signal. This is primarily attributed to the absence of secondary structure confinement of the probe within a high‐concentration reaction environment. This entirely flexible single‐stranded state greatly enhances the kinetic probability of nonspecific encounters and leakage activation, thus strongly generating background noise. In addition, although probes with duplex stems enhance stability, they also introduce dual liabilities. Cognate DNA sequences serve as competing off‐target substrates for Cas12a, leading to wasted enzyme activity and minor steric interference, which together reduce the effective signal output. In contrast, the probe without a stem guarantees that the enzyme's cleavage activity is fully focused on the intended site. In conclusion, the 3’D_(5,0)_‐SR configuration minimizes non‐productive depletion of enzymatic activity and maximizes target site accessibility, leading to an optimal synergistic improvement in Cas12a's recognition and cleavage efficiency.

Having identified the 3’ D_(5,0)_‐SR as the most effective probe of conformational accessibility, we investigate the potential of adding a biotin tag at the terminus of the scaffold RNA, which might adjust the probe‐protein interface through modulating the local hydrophilicity‐lipophilicity balance or introducing subtle steric effects. To validate this hypothesis, we developed several 3’D‐SR variants with terminal extensions of 2, 3, or 5 DNA bases with biotin modifications (Figure S26a–f). A systematic comparison of their performance to that of unmodified biotin 3’D‐SR was carried out under rigorously controlled parallel conditions. Experimental outcomes demonstrated that the3’D_3B_‐SR outperforms the 3’D_5_‐SR, emphasizing that biotin modification improves probe‐protein interactions without significantly hindering enzymatic cleavage. To further enhance the signal output of the cascade reaction, we integrated a flexible tetraethylene glycol (TEG) linker between the biotin and the DNA (Figure S26g–i). Results indicate that the flexible TEG spacer may spatially isolate the biotin moiety from the protein's cleavage active site, thereby eliminating potential interference and preserving optimal cleavage efficiency. Concurrently, it provides the biotin with sufficient conformational freedom to adopt an optimal orientation for effective binding to Cas12a, ultimately maximizing the signal enhancement efficiency of the positive feedback circuit. Subsequent molecular docking and free energy analysis further confirmed this finding (Figure S26j–o).

### 2.6 Evaluation of Detection Performance of the CALCas System

Following our previous optimization of experimental parameters, we conducted a comprehensive system‐level assessment of the performance of three detection methods: one‐step method, two‐step homogeneous method, and two‐step magnetic bead‐assisted method (Figure S27). Although the one‐step method is operationally simple, the competition and interference among components may result in suboptimal cleavage of the 3’D‐SR, producing a net signal value significantly inferior to those of the other two approaches. In the two‐step magnetic bead‐based method, multiple washing steps elute weakly bound or dissociated target molecules‐a loss negligible in absolute terms at high concentrations, yet at low concentrations, where even femtomolar losses per wash can compromise the method's sensitivity. In contrast, the two‐step homogeneous strategy facilitates temporal separation of cleavage from the subsequent detection step, thereby enabling full utilization of the structural advantages of 3’D‐SR and leading to dependable, high‐intensity detection signals. Our experimental results clearly demonstrate that this two‐step homogeneous method achieves significantly superior sensitivity compared to other approaches. Consequently, we selected this optimal format for all subsequent sample detection experiments.

To assess the detection performance of the CALCas system, we performed parallel comparisons with the single Cas12a system (Figure 5a). Remarkably, the CALCas system achieved a detection limit of ∼1 fM, corresponding to an approximate 10,000‐fold enhancement in sensitivity compared with the single Cas12a system (Figure 5b–d and Figures S27e–h and S28a). This result underscores its exceptional capacity to detect trace amounts of target without additional amplification. Specificity validation using two non‐target nucleic acids revealed that only the target sequence triggered substantial fluorescence enhancement, whereas all non‐target sequences generated signals comparable to the negative control (Figure 5e and Figure S28b). Moreover, relative to the reported Asymmetric CRISPR approach (Figure S27m–p), our system showed a 100‐fold gain in detection sensitivity (1 fM versus 100 fM) without compromising specificity. Collectively, these findings highlight that the CALCas system achieves markedly higher sensitivity than the Asymmetric CRISPR approach without compromising specificity, emphasizing its strong potential for practical detection applications.

**FIGURE 5:**
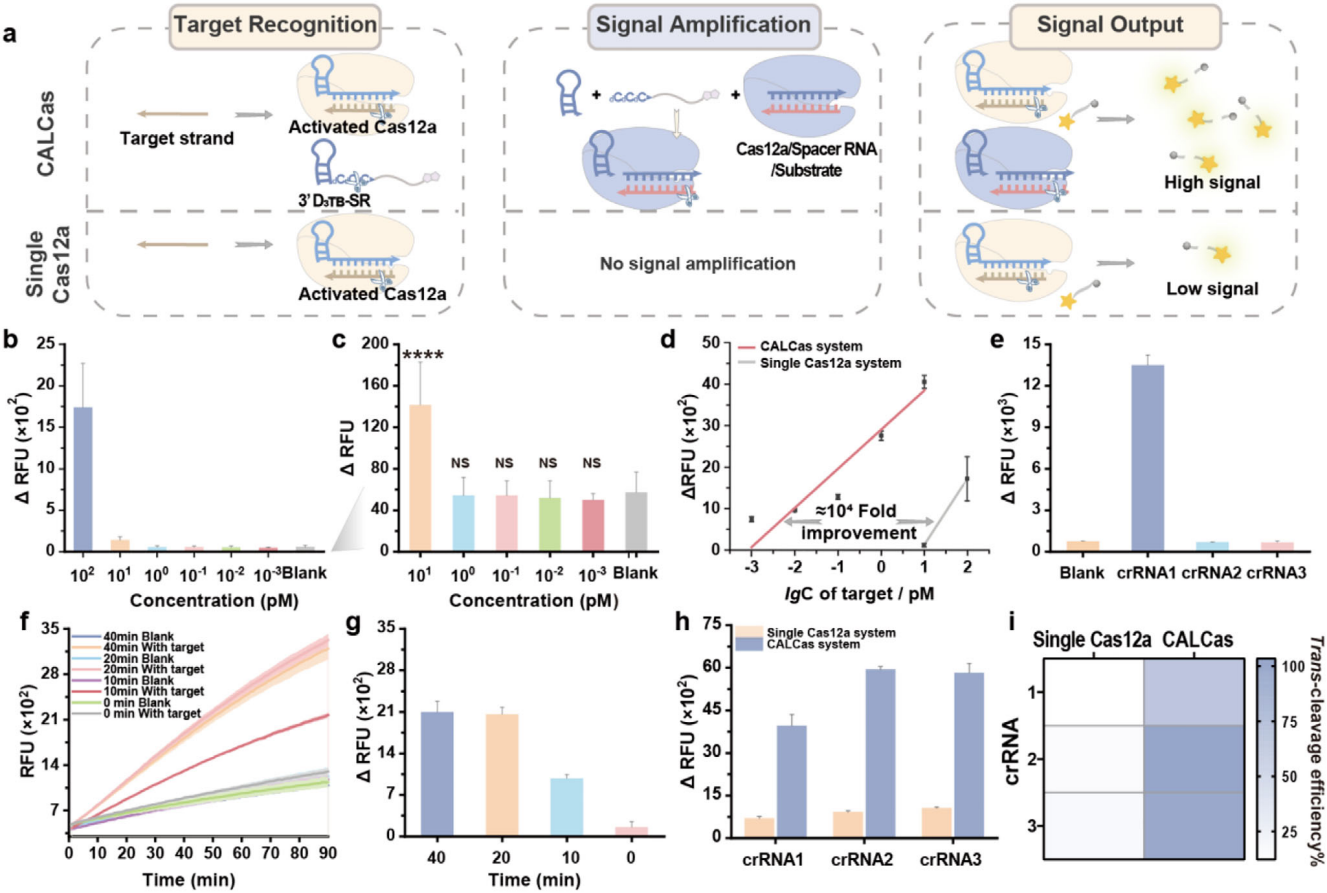
Detection performance of the CALCas system. (a) Schematic diagram of the differences between CALCas and single Cas12a systems. (b,c) ΔRFU of the target DNA from 100 pM to 1 fM, using the single Cas12a system. (d) Relationship between ΔRFU and concentrations of the target in the single Cas12a system and CALCas system. (e) Specificity of the CALCas system for the detection of different targets. (f,g) Using different times to detect the target strand. (h,i) Comparing the ΔRFU of the CALCas and single Cas12a (The combination with the highest *trans‐*cleavage efficiency was set as 100%). Data are expressed as mean ± SD (*n* = 3). The levels of significance relative to the control group were assessed by one‑way ANOVA with Dunnett's post‑hoc test and are denoted as ^****^, *p* < 0.0001; NS, not significant.

To boost detection efficiency without compromising performance, we systematically optimized the timeline of the CALCas system. The first step takes only 20 min, and the target amplification signal can reach a plateau, which ensures the robustness of the initial reaction. The second step takes 10 min to distinguish between the positive samples and the negative control. Consequently, the entire assay can be completed within 30 minutes, significantly reducing the overall processing time to satisfy the demands for rapid diagnosis (Figure 5f,g).

Meanwhile, to assess the general applicability of the 3’D‐SR as a signal‐transducing center, we evaluated detection performance using various crRNAs. The CALCas system performs consistently better in signal enhancement than the single Cas12a system (Figure 5h–i and Figure S29). Overall, utilizing the 3’D‐SR architecture that effectively decouples the target recognition from the cleavage activity of Cas12a, the CALCas system established in this research not only reaches superior levels of detection sensitivity and specificity without the requirement for pre‐nucleic acid amplification, but also guarantees its versatility and reliability.

### 2.7 Assessing the Clinical Performance of the CALCas System

Viral nucleic acid in host tissue and body fluid has been demonstrated to be inextricably associated with early clinical diagnosis, monitoring of disease progression, and prognostic assessment of multiple viral infectious diseases. The persistent infection of human papillomavirus (HPV) 16 and HPV18 constitutes the most significant predictor for the progression to cervical intraepithelial neoplasia and invasive cancer. In this study, the CALCas system was employed to detect nucleic acids of HPV 16 and HPV18 (Figure 6a). Preliminary assessment revealed high detection sensitivity with a detection limit of 10 and 1 fM, respectively (Figure 6b–e and Figure S30a,b), and moreover, the system shows excellent specificity, with no significant cross‐reactivity signals found in comparative tests against non‐target sequences (Figure S30c–f). Subsequently, total DNA was extracted from cervical secretions obtained from 60 subjects. Of them, 21 were identified to be HPV16 DNA‐positive, 2 were HPV18 DNA‐positive, and 1 was double‐positive for HPV16 and HPV18 DNA, whereas the remaining were HPV16 and HPV18 DNA‐negative using a commercial real‐time PCR assay kit. All the DNA samples were analyzed using the CALCas system (Figure 6f–i and Figures S30g–l and S31 and S32). The Cas12a assay demonstrated high concordance with the real‐time PCR findings, except for three discordant cases (samples 23, 31, and 49). The false‐negative results for samples 23 and 31 may be due to the target copy number falling below the effective limit of detection (LOD) of the CALCas system. Meanwhile, the false‐positive signal in sample 49 could be related to non‐specific triggering or cross‐reactivity in the Cas reaction. For a comprehensive assessment of the assay's diagnostic efficacy, we constructed a Receiver Operating curve (ROC) and found it had a superior ability to distinguish positive from negative cases (Figure S30k,l). Collectively, these findings substantiate the method's high accuracy and specificity for HPV16/18 DNA detection in cervical samples, underscoring its reliable clinical diagnostic potential.

**FIGURE 6:**
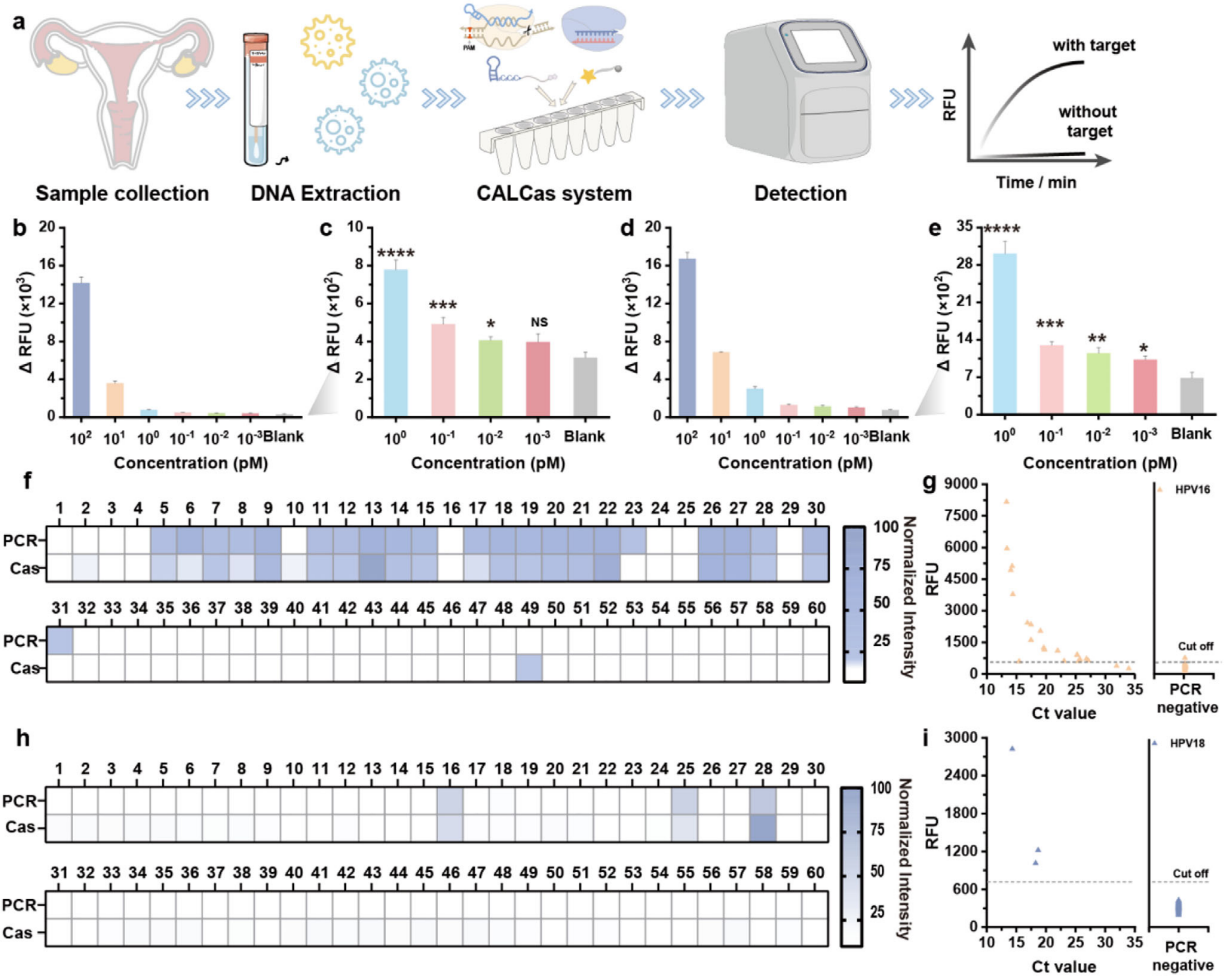
Clinical performance of the CALCas system. (a) Graphic illustration of the CALCas system for detecting HPV DNA in clinical samples. (b,c) Sensitivity analysis for testing HPV16 DNA from 100 pM to 1 fM. (d,e) Sensitivity analysis for testing HPV18 DNA from 100 pM to 1 fM. (f–i) Comparison of analytical performance between the CALCas system and the real‐time PCR assay. Fluorescence intensity is displayed in the histogram (f,h). Grouped scatter plots illustrating the detection results for HPV 16 and HPV 18 in clinical samples (g,i). Data are expressed as mean ± SD (*n* = 3). The levels of significance relative to the control group were assessed by one‑way ANOVA with Dunnett's post‑hoc test and are denoted as ^****^, *p* < 0.0001; ^***^, *p* < 0.001; ^**^, *p* < 0.01; ^*^, *p* < 0.05; NS, not significant.

## 3 Conclusion

This investigation is dedicated to engineering the crRNA structure to reveal the underlying mechanisms that fine‐tune Cas12a activity. The exploration of engineered crRNA variants uncovered an important mechanistic insight in which Cas12a can recognize target DNA without the complete continuous conformation of the crRNA. Whether the crRNA is divided, modified with biotin or DNA at non‐critical positions, or engineered into closed topological forms, the main recognition capability of Cas12a is unaffected. Conversely, adding DNA to the 3’ end of the scaffold RNA can hinder the *trans*‐cleavage activity of Cas12a by occupying the limited space within Cas12a. This reveals that 3’D‐SR functions as a powerful “allosteric switch” that separate the recognition and cleavage activities of Cas12a, providing essential structural biological insights and a foundational technical pathway for creating activity‐controllable Cas12a systems.

By utilizing 3’D‐SR, we created an innovative molecular switch platform for CRISPR/Cas12a. In this system, the 3’D‐SR serves not just as a sophisticated switch, but also as a signal amplifier to enhance the output. Upon activation by the initial Cas12a protein, this 3’D‐SR functions as the driver of a precise molecular logic circuit, immediately alleviating the inhibition on the downstream Cas12a protein and initiating a robust cascaded amplification response. This design successfully creates a physical shield for the downstream Cas12a, suppressing non‐specific cleavage or leakage at the molecular level. This effectively addresses the common challenge of concurrent background noise amplification in high‐sensitivity systems, enabling the unification of the high net signal with high sensitivity.

Nucleic acid detection platforms building on these philosophies have successfully achieved over 10,000‐fold increased sensitivity without adding additional amplification. This pioneering study of engineering crRNA hub architecture serves as an essential technical pathway to controllably reprogram Cas enzymatic activities. Its practical potential in diagnostics has also been well proven through specific clinical scenarios for patient HPV detection. Hence, from molecular mechanism exploration to enzyme activity programming, from biosensor design to real‐life application, our strategy in CRISPR/Cas biosensing development provides innovative perspectives for the advancement of next‐generation deployable molecular diagnostic tools.

## 4 Experimental Section

### 4.1 Materials and Reagents

All the DNA and RNA oligonucleotides were synthesized and purified by General Biosystems Co., Ltd. (Chuzhou, Anhui, China), with sequences detailed in Table S1. GenCRISPR LbCas12a Nuclease from Lachnospiraceae bacterium ND2006 was obtained from Genscript Biotech Co., Ltd. (Nanjing, Jiangsu, China). NEBuffer^TM^ r2.1 (10 mM Tris‐HCl, 50 mm NaCl, 10 mm MgCl_2_, 100 µg/mL Recombinant Albumin, pH 7.9, 1×) and NEBuffer^TM^ 3.0 (100 mm NaCl, 50 mm Tris‐HCl, 10 mm MgCl_2_, 1 mm DTT, pH 7.9, 1×) were purchased from New England Biolabs (Beijing, China). TE (Tris‐ethylenediaminetetraacetic acid) buffer, 4R GelRed nucleic acid gel stain (10000×), Acryl/Bis 30% Solution (29:1) and RNase‐free water were acquired from Sangon Biotech Co., Ltd. TEMED was offered by Beyotime Biotechnology (Shanghai, China). The 6x loading buffer and DNA Marker‐20 bp DNA Ladder were offered by Takara Biotech, Inc. (Dalian, Liaoning, China). Streptavidin magnetic beads (SA‐MBs, 300 nm) were from Suzhou Beaver Biomedical Engineering Co., Ltd. (Suzhou, Jiangsu, China). Nucleic acid templates included HPV16/18 DNA plasmid synthesized by Tyhygene (Wuhan, Hubei, China). One Step PrimeScript^TM^ RT‐qPCR Mix from Vazyme (Nanjing, China). All chemicals were of analytical grade and used without further purification.

### 4.2 Preparation of dsDNA Substrate, Topological Structures and Hairpin 3’D‐SR

To obtain the dsDNA substrate, equal concentrations and volumes of the targeting strand (TS) and non‐targeting strand (NTS) were mixed in 1× NEBuffer^TM^ 3.0. The mixture was then heated to 95°C for 5 min and gradually cooled to 25°C. The annealed products (1 µM) were finally stored at −20°C for further use.

For different topological structures and a hairpin 3’D‐SR, they were mixed in 1× NEBuffer^TM^ 3.0, yielding a final strand concentration of 1 µM. This mixture followed the same procedure as detailed previously.

### 4.3 Evaluation of the Inhibitory Effect on Cas12a Cleavage Activity

To analyze the inhibitory effect on Cas12a cleavage activity, various modifications (including biotin or DNA) of split crRNA were utilized. A 25 µL Cas12a reaction pre‐mixture was prepared, containing Cas12a (12.5 nm), scaffold RNA (10 nm), spacer RNA (10 nm), substrate (10 nm), TB‐Reporter (1000 nm), and RNase‐free water in 1× NEBuffer^TM^ r2.1. The modified crRNA (with alterations in the scaffold or spacer region) served as the experimental groups, while the standard, unmodified split crRNA was used as the standard control. Subsequently, these reactions were immediately transferred to a fluorescence plate reader and incubated at 37°C for 90 min on a Gentier 96R Automatic Medical PCR Analysis System, with fluorescence measurements taken every minute.

For the inhibition experiments of engineered topological structures on Cas12a endonuclease activity, a 25 µL Cas12a reaction pre‐mixture was prepared, containing Cas12a (12.5 nM), different topological structure complexes (10 nm), TB‐Reporter (1000 nm), and RNase‐free water in 1×NEBuffer^TM^ r2.1. Reactions containing the topological structure were designated as experimental groups, while a standard, unmodified split crRNA or crRNA reaction served as the standard control. Fluorescence signal output followed the same procedure as detailed previously.

### 4.4 Time‐Resolved Fluorescence Assay for Stepwise Complex Assembly

To characterize the conformational dynamics during sequential component addition, the fluorescence‐based cleavage assay was performed as follows. Prepare a 186 µL pre‐mix solution consisting of 2 µL of 5’ Cy3 WT substrate (5 µM) in 1× NEBuffer^TM^ r2.1. Transfer this mixture to a 96‐well black microplate, incubate at 37°C, and immediately start monitoring baseline fluorescence intensity (*F*_530 → 660_, *F*_530 → 565,_ *F*_630 → 660_) using the SpectraMax iD3, recording data at 1 min intervals. After 10 min of incubation, add 2 µL of the 3’ Cy5 WT Spacer RNA (5 µM) to the above mixture. Continue monitoring fluorescence intensity. After another 20 min, add 10 µL Cas12a (1 µM) and 2 µL WT scaffold RNA/3’D‐SR (5 µm) to the system (final volume: 200 µL). Maintain the 37°C incubation and continue recording fluorescence intensity for an additional 60 min (total assay duration: 90 min).

### 4.5 Non‐denatured Polyacrylamide Gel Electrophoresis (PAGE) Analysis

To detect the formation of hairpins, the denatured and undenatured strands were mixed with 6 × loading dye and carried out in 1×TBE buffer (90 mM Tris‐boric acid, 2 mM EDTA, pH 8.4) at 110 V constant voltage for 45 min, and stained with GelRed. Afterward, the gel was imaged using the multichannel ChemiDoc Imaging System (Bio‐Rad).

For the binding interaction test between the protein and nucleic acid strand, a total of 25 µL of the reaction solution containing different fluorescent nucleic acids (200 nm) and Cas12a (200 nM) was prepared by mixing in 1×NEBuffer^TM^ r2.1 buffer and RNase‐free water. After incubation of the reaction solution at 37°C for 60 min. The image output followed the same procedure as detailed previously, but synchronous image acquisition and analysis were achieved by configuring the parameters of different fluorescence channels (Cy3, Cy5, and GelRed).

For the purpose of validating the hypothesis that 3'D‐SR can be cleaved by the activated primary Cas12a, which in turn achieves the accurate release of scaffold RNAs, PAGE was employed as the verification assay. A total of 25 µL of the reaction solution containing crRNA (200 nm), Cas12a (200nm), WT scaffold RNA/3’D‐SR (200 nm), and TS (2nm) was prepared by mixing in 1×NEBuffer^TM^ r2.1 buffer and RNase‐free water. After incubation of the reaction solution at 37°C for 60 min. The image output followed the same procedure as detailed previously.

### 4.6 Exploring the Significance of Scaffold RNA and 3’D‐SR

To analyze the significance of scaffold RNA on Cas12a cleavage activity, we prepared a 25 µL Cas12a reaction pre‐mixture containing Cas12a (10 nm), scaffold RNA (10 nm), WT spacer RNA (1 nm), WT substrate (1 nm), TB‐Reporter (1000 nm), and RNase‐free water in 1×NEBuffer^TM^ r2.1. Scaffold RNA was used as the positive control and without scaffold RNA was used as the negative control. Subsequently, the fluorescence signal output was measured using the same procedure as detailed previously.

To assess the inhibitory performance of the CALCas system, we prepared a 25 µL reaction containing Cas12a (60 nm), WT scaffold RNA or 3’D‐SR (10 nm), WT spacer RNA (1 nm), WT substrate (1 nm), TB‐Reporter (1000 nm), and RNase‐free water in 1×NEBuffer^TM^ r2.1. For the competition test, a 17.5 µL reaction system was prepared containing Cas12a (60 nm), crRNA (40 nm), WT scaffold RNA or 3’D‐SR (10 nm), and RNase‐free water in 1× NEBuffer^TM^ r2.1. The mixed solutions were incubated for 1 h at 37°C. Then, the above solution was added to the reaction solutions containing WT spacer RNA, WT substrate, and TB‐Reporter, supplemented to 25 µL. Subsequently, the fluorescence signal output followed the same procedure as detailed previously.

### 4.7 Construction of the DNA‐MBs Nanointerface

Before conjugation, 20 µL of SA‐MBs (10 mg/mL) was washed three times and resuspended in 200 µL of NEBuffer^TM^ r2.1. Then, 50 µL of 5’ Biotin‐3’ DNA Cy3 scaffold RNA (200 nM) was added to 50 µL of SA‐MBs (1 mg/mL), which was mixed and shaken at room temperature for 1 h. The resultant DNA‐conjugated MBs were separated from excess DNA strands by a magnet and rinsed three times with the NEBuffer ^TM^ r2.1.

### 4.8 Magnetic Bead Capture Experiments

A total of 100 µL of the reaction solution containing Cas12a (25 nm), crRNA‐1 (25 nm), TS‐1 (1 nm), and DNA‐MBs was prepared by mixing in 1×NEBuffer^TM^ r2.1 buffer and RNase‐free water. After incubation of the reaction solution at 37°C for 60 min, the released fragments were separated by the magnet and measured by the FL‐970 spectrophotometer.

### 4.9 The Single Cas12a System for Nucleic Acid Detection

Cas12a/crRNA complex (10 nm) and different concentrations of TS and TB‐Reporter (1000 nm) were added to 1×NEBuffer^TM^ r2.1 buffer. The total reaction volume was then adjusted to 25 µL with DEPC‐treated water. Subsequently, the fluorescence signal output followed the same procedure as detailed previously.

### 4.10 One‐Step CALCas System for Nucleic Acid Detection

Cas12a (70 nm), crRNA complex (10 nm), different concentrations of TS, 3’D‐SR (60 nm), WT spacer RNA (10 nm), WT substrate (10 nm), and TB‐Reporter (1000 nm) were added to 1×NEBuffer^TM^ r2.1 buffer. The total reaction volume was then adjusted to 25 µL with DEPC‐treated water. Subsequently, the fluorescence signal output followed the same procedure as detailed previously.

### 4.11 CALCas System with MB for Nucleic Acid Detection

First, a mixture containing Cas12a/crRNA (10 nm), different concentrations of TS, and 3’D‐SR (60 nm) and NEBuffer^TM^ r2.1 was prepared in a total volume of 10.5 µL, adjusted with DEPC water. Then, 5 µL MB was added to the above mixture for 10 min at 37°C. Then, the uncleaved scaffold RNA was separated by MB. After that, Cas12a (60 nm), WT spacer RNA (10 nm), WT substrate (10 nm), and TB‐Reporter (1000 nm) were added to the above mixture with total 25µL. Finally, the fluorescence signal was measured immediately as detailed previously.

### 4.12 Two‐step CALCas System for Nucleic Acid Detection

First, a mixture containing Cas12a/crRNA (10 nM), different concentrations of target strand, and 3’D‐SR (60 nm) and NEBuffer^TM^ r2.1 was prepared in a total volume of 15.5 µL, adjusted with DEPC water. This mixture was incubated at 37°C for 20 min, followed by adding Cas12a (60 nm), WT spacer RNA (10 nm), WT substrate (10 nm), and TB‐Reporter (1000 nm) to the above mixture with total 25µL. Finally, the fluorescence signal was measured immediately as detailed previously.

### 4.13 Nucleic Acid Extraction of Clinical Samples

The clinical DNA samples used in this study were extracted from swab samples using the nucleic acid DNA Kit, following the respective manufacturer's protocols. The obtained nucleic acid products were stored at −80°C for further use.

### 4.14 Analysis of Clinical DNA Samples by qPCR

A 20 µL qPCR reaction mixture was prepared using 2x ChamQ Blue Universal SYBR qPCR Master Mix and primers (10 µM each). To each reaction, 2.5 µL of DNA extracted from clinical samples was added. For HPV16/18 samples, the qPCR thermal cycling program is set as follows: pre ‐denaturation at 95°C for 30 s, followed by 40 cycles of amplification (95°C for 10 s and 60°C for 30 s).

### 4.15 Statistical Analysis

All statistical analyses were performed using GraphPad Prism 9.5.1 and Origin 2018. Data were normalized to the control group and expressed as a percentage of the control value. Additionally, data are presented as mean ± SD from three independent experiments (n = 3). Statistical differences between groups were analyzed via one‐way ANOVA followed by Dunnett's post‐hoc test. *P* values are defined as follows: ^****^, *p* < 0.0001; ^***^, *p* < 0.001; ^**^, *p* < 0.01; ^*^, *p* < 0.05; NS, not significant. The specific statistical tests used are provided in the corresponding figure legends.

## Author Contributions

**Xingrong Li**: conceptualization, validation, methodology, writing – original draft, writing – review and editing, data curation, investigation, formal analysis. **Honghua Hu**: visualization, resources, validation, data curation, investigation. **Xinli Jiang**: writing – original draft, data curation, visualization, project administration, investigation. **Xuhuai Fu**: data curation, resources. **Lin Hu**: writing – original draft, data curation, visualization. **Qinghua Wu**: project administration, resources. **Jiatong Chai**: writing – review and editing, writing – original draft, conceptualization, methodology, supervision, funding acquisition. **Diangeng Li**: writing – review and editing, conceptualization. **Jinhong Guo**: writing – review and editing, conceptualization. **Zhiqiang Li**: writing – review and editing, methodology, conceptualization, supervision. **Yi Li**: conceptualization, methodology, funding acquisition, writing – review and editing, supervision. **Yirong Li**: writing – review and editing, writing – original draft, conceptualization, methodology, supervision, funding acquisition.

## Ethics Statement

The studies involving human clinical samples were approved by the ethics committee of Sichuan Provincial People's Hospital (Approval No. 2025642). The participants provided their written informed consent to participate in this study.

## Conflicts of Interest

The authors declare no conflicts of interest.

## Supporting information




**Supporting File 1**: advs78246‐sup‐0001‐SuppMat.pdf.

**Supporting File 2**: advs78246‐sup‐0002‐SuppMat.pdf.

## Data Availability

The data that support the findings of this study are available from the corresponding author upon reasonable request.
