# Supplementary material for: Scaffold RNA Reengineering for a Leakage‐Suppressed CRISPR/Cas12a Cascade System Toward Precise Nucleic Acid Diagnostics
Source: Adv Sci (Weinh). 2026 Oct 22:e78246. Online ahead of print. doi: 10.1002/advs.78246 (PMC13690970; doi:10.1002/advs.78246)
Supplement: Supplementary file 1 [file ADVS-9999-e78246-s001.pdf]

Supporting Information

Scaffold RNA Reengineering for a Leakage-Suppressed CRISPR/Cas12a Cascade System toward Precise Nucleic Acid Diagnostics

Xingrong Li [a, 1], Honghua Hu [c,b,1], Xinli Jiang [a,1], Xuhuai Fu [b], Lin Hu [b], Qinghua Wu[b], Jiatong Chai [a], Diangeng Li [h], Jinhong Guo [b], Zhiqiang Li [i*], Yi Li [b,g*], Yirong Li [a,d,e,f*]

[a] *Department of Clinical Laboratory, Zhongnan Hospital of Wuhan University, Wuhan 430071, China.*

[b] *Key Laboratory of Clinical Laboratory Diagnostics (Ministry of Education), College of Laboratory Medicine, Chongqing Medical University, Chongqing 400016, China*

[c] *Department of Laboratory Medicine and Sichuan Provincial Key Laboratory for Human Disease Gene Study, Sichuan Provincial People's Hospital, University of Electronic Science and Technology of China, Chengdu 610072, China*

[d] *Hubei Provincial Clinical Medical Research Center for Molecular Diagnostics, Wuhan 430071, China.*

[e] *Wuhan Research Center for Infectious Diseases and Cancer, Chinese Academy of Medical Sciences, Wuhan 430071, China.*

[f] *Hubei Engineering Center for Infectious Disease Prevention, Control and Treatment, Wuhan 430071, China.*

[g] *Western Institute of Digital-Intelligent Medicine, Chongqing 401329, China*

[h] *Department of Academic Research, Beijing Ditan Hospital, Capital Medical University, National Center for Infectious Diseases (Beijing), 8th Jingshun East Road, Beijing 100015, China*

[i] *Brain Glioma Center, Zhongnan Hospital of Wuhan University, Wuhan University, Wuhan 430071, China.*

---

*Corresponding Author.

E-mail: liyirong838@163.com; yi.li@cqmu.edu.cn; lizhiqiang@whu.edu.cn

[1] These authors contributed equally to this study.

Apparatus

DNA and RNA suspensions were quantified by the NanoDrop One spectrophotometer from Thermo Fisher Scientific (Waltham, MA, USA). The Mini Dry Bath MDB100C from JOANLAB Equipment Co., Ltd (Zhejiang, China) was used for DNA complex annealing and protein inactivation. The insulated electric thermostatic incubator and electric thermostatic three-hole water bath were from Yuejin Medical Instrument Co., Ltd (Shanghai, China). The low-speed centrifuge and oscillating vortex blender were supplied by DLAB Scientific Co., Ltd. (Beijing, China). Fresco™ 21 Microcentrifuge was supplied by Thermo Fisher Scientific Co., Ltd. (Shanghai, China). Gentier 96R Automatic Medical PCR Analysis System from Tianlong Technology Co., Ltd. (Xi'an, Shaanxi, China) detects all fluorescence spectra. Gel electrophoresis was performed on the electrophoresis apparatus (Bio-Rad, USA). The gel image was captured by the ChemiDoc XRS and ChemiDoc MP system (Bio-Rad, USA). The FL-970 spectrophotometer is from Techcomp company (Shanghai, China). The SpectraMax® iD3 is from Molecular Devices (Shanghai, China). The magnetic rack is from Tiangen Biotech Co., Ltd (Beijing, China). The TR-02U test tube rotary mixer was purchased from Jiangsu Jiemei Investment Development Group Co., Ltd (Suzhou, Jiangsu, China).

Molecular docking simulations

Initially, the LbCas12a structure (PDB ID: 8Y03) was downloaded from the worldwide PDB (Protein Data Bank) database. Then, the obtained 8Y03 complex structure was processed using PyMOL to independently generate the PDB file for the LbCas12a protein[1]. AlphaFold3 server (https://alphafoldserver.com) was employed to predict the nucleic acid structures of scaffold RNA by inputting its sequences[2]. Subsequently, the predicted nucleic acid structures obtained from AlphaFold3 were processed in PyMOL to generate their respective files, and based on these, three-dimensional models of the modified nucleic acid molecules were specifically constructed for subsequent conformational analysis. Docking simulations were set up where LbCas12a served as the receptor, and WT scaffold RNA/3'D-SR, WT spacer and WT substrate were each used as ligands. The resultant complexes were visualized and analyzed using PyMOL to assess the interactions and structural conformations.

FRET assay

To elucidate the binding interaction between the protein and nucleic acid strand, we performed fluorescence spectroscopy. A 100 μL reaction mixture was prepared in 1×NEBuffer^{TM} 2.1, containing Cas12a (100 nM) along with fluorescently labeled or unlabeled versions of WT scaffold RNA or 3'D-SR (100 nM), WT spacer RNA (100 nM), and substrate (100 nM). Then, all measurements were performed on an FL-970 spectrophotometer, with three independent replicates (n = 3) for each sample. For fluorescence signal detection, three measurement channels were employed sequentially: (1) the donor channel (Cy3 excitation→Cy3 emission), with the excitation wavelength (λ_{ex}) set to 530 nm and recording of the emission wavelength (λ_{em}) at 565 nm (Cy3 emission maximum), denoted as $F_{530\rightarrow565}$; (2) the aceptor direct excitation channel (Cy5 excitation→Cy5 emission), where λ_{ex} was set to 630 nm with subsequent scanning and recording of the λ_{em} at 660 nm (Cy5 emission maximum), denoted as $F_{630\rightarrow660}$; and (3) the FRET sensitized emission channel (Cy3 excitation→Cy5 emission) was measured by setting the λ_{ex} to 530 nm and recording the λ_{em} at 660 nm, denoted as $F_{530\rightarrow660}$.

To obtain the specific FRET signal (free of non-FRET background interference), dual-background subtraction was performed as follows:

1. Acceptor direct excitation ratio: Calculated as the ratio of fluorescence intensity of the Cy5-only sample excited at 530 nm and detected at 660 nm ($F^{\text{Cy5-only}}_{530\rightarrow660}$) to that excited at 630 nm and detected at 660 nm ($F^{\text{Cy5-only}}_{630\rightarrow660}$), denoted as $R_{\text{direct}} = \frac{F^{\text{Cy5-only}}_{530\rightarrow660}}{F^{\text{Cy5-only}}_{630\rightarrow660}}$.

2. Donor leakage ratio: Calculated as the ratio of fluorescence intensity of the Cy3-only sample excited at 530 nm and detected at 660 nm ($F^{\text{Cy3-only}}_{530\rightarrow660}$) to that excited at 530 nm and detected at 565 nm ($F^{\text{Cy3-only}}_{530\rightarrow565}$), denoted as $R_{\text{leak}} = \frac{F^{\text{Cy3-only}}_{530\rightarrow660}}{F^{\text{Cy3-only}}_{530\rightarrow565}}$.

3. Net FRET signal:

$$F_{\text{FRET}} = F^{\text{DA}}_{530\rightarrow660} - (R_{\text{direct}} \times F^{\text{DA}}_{630\rightarrow660}) - (R_{\text{leak}} \times F^{\text{DA}}_{530\rightarrow565})$$

4. FRET Ratio = $\frac{F_{\text{FRET}}}{F^{\text{DA}}_{530\rightarrow565}}$

5. FRET Ratio Result Interpretation:

A ratio in the range of 0.3-3.0 was defined as "effective FRET". A ratio between

0.1 and 0.3 was categorized as "weak FRET", reflecting partial energy transfer but with relatively low efficiency. For ratios ranging from 0 to 0.1 (classified as "suspected background"), no effective energy transfer was detected, suggesting a lack of sufficient proximity between the fluorophores.

Michaelis-Menten kinetics calculation

The Michaelis-Menten kinetics were measured following the protocol given by a previous report. The experimental procedures were as follows: Cas12a (10 nM) nuclease was incubated with crRNA (10 nM) and a high concentration of target strand (10 nM) in 1×NEBuffer r2.1 to form the active Cas12a-crRNA-ssDNA ternary complex. Then, the activated complex was used to cleave a dilution series of TB-Reporter (including 0, 31.25, 62.5, 125, 250, 500, 1000, and 2000 nM) at 37℃. The endpoint fluorescence intensity (F_{cl}') was recorded for each probe concentration. The net fluorescence signal attributable to probe cleavage (F_{cl}) for each concentration was calculated by subtracting the background saturation signal from the no-TB-Reporter control (F_{cl0}, at 0 nM) using the formula (1).

$$F_{cl} = F_{cl'} - F_{cl0} \quad (1)$$

In parallel, a no-template control was set up by replacing the target strand with RNase-free water. The saturated background fluorescence intensity ($F_{ucl'}$) for each ssDNA probe concentration in this control was recorded. The net background fluorescence (F_{ucl}) corresponding to the uncut probe at each concentration was then determined by subtracting the saturation value in the absence of probe (F_{ucl0}, at 0 nM) from $F_{ucl'}$, according to equation (2).

$$F_{ucl} = F_{ucl'} - F_{ucl0} \quad (2)$$

Since the quenching of a fluorophore in an intact reporter is imperfect, the background-subtracted fluorescence F_{total} measured during the *trans*-cleavage experiment is the sum of fluorescence from cleaved reporters F_{cl} and uncleaved reporters F_{ucl}.

$$F_{total} = F_{cl} + F_{ucl} \quad (3)$$

Thus, by displacing the calibration curve equations from Supplementary Figure S20 in Equation (3), we obtain the following equation:

$$F_{total} = 6.12\, C_{cl} + 0.12\, C_{ucl} \quad (4)$$

From mass conservation, C_{cl}+ C_{ucl}= C_{total}, where C_{total} is the initial concentration

of uncleaved reporters. Thus, Equation (4) can be rewritten as

$$F_{\text{total}} = 6.12\ C_{\text{cl}} + 0.12(C_{\text{total}} - C_{\text{cl}}) = 6.00\ C_{\text{cl}} + 0.12C_{\text{total}}\ (5)$$

The cleaved reporter concentration C_{cl} (in nM) versus time is estimated using Equation (5) as

$$C_{\text{cl}}\ (\text{in nM}) = \frac{F_{\text{total}} - 0.12C_{\text{total}}}{6.00}\ (6)$$

In this way, we are able to calculate the reaction velocity in nM by measuring the fluorescence enhancement rate.

When testing the reaction rate of the *trans*-enzyme cleavage reaction, first, 10 nM Cas12a, 10 nM crRNA, and 10 pM target nucleic acid are mixed to form a Cas12a/crRNA/target ternary complex. Then, a series of concentration gradients of TB-Reporter is added. The reaction is carried out at 37°C for 1.5 h. At the same time, a blank control group is set up, and the background fluorescence value of the blank control group is deducted. The fluorescence signal is converted into the concentration for cutting the TB-Reporter using the equation obtained in (6). Similarly, the CALCas system and the engSplit RNP/DNA complex response measurements are consistent with the above.

The regular Michaelis-Menten equation can eventually be written in the following form:

$$v = \frac{d[P]}{dt} = k_{cat} E_0 \frac{[S]}{K_m + [S]}\ (7)$$

where k_{cat} is the catalytic turnover rate of the enzyme, E_0 is the enzyme concentration (10 pM in this experiment), $[S]$ is the substrate concentration, and K_m is the Michaelis–Menten constant.

The measured reaction velocities versus reporter (substrate) concentration data were fitted to the Michaelis–Menten equation using GraphPad Prism 9 software to obtain k_{cat} and K_m.

Table S1. Oligonucleotide sequences used in this study.

Name		Sequence (5'-3')
WT scaffold RNA		UAAUUUCUACUAAGUGUAGAU
WT spacer (G/3) RNA		GACAUAGCACAUAGACUGAGACUG
WT substrate(C/3) DNA		CAGTCTCAGTCTATGTGCTATGTC
5'Biotin scaffold RNA		Biotin-UAAUUUCUACUAAGUGUAGAU
3'Biotin scaffold RNA		UAAUUUCUACUAAGUGUAGAU-Biotin
5'Biotin spacer RNA		Biotin-GACAUAGCACAUAGACUGAGACUG
3'Biotin spacer RNA		GACAUAGCACAUAGACUGAGACUG-Biotin
3'DNA(C) scaffold RNA		UAAUUUCUACUAAGUGUAGAU/dC//dC//dC//dC//dC/
3'Biotin-DNA scaffold RNA		UAAUUUCUACUAAGUGUAGAU/dC//dC//dC//dC//dC/-Biotin
5'DNA spacer RNA		/dC//dC//dC//dC//dC/GACAUAGCACAUAGACUGAGACUG
circularized architecture I	Closed-loop crRNA	/dC//dC//dT//dT//dT//dG//dG//dA//dC//dA//dT//dA//dG//dC//dA//dC//dA//dT//dA//dG//dA//dC//dT//dG//dA//dG//dA//dT//dG//dC//dA//dC//dC//dC//dC//dC//dC/UAAUUUCUACUAAGUGUAGAUCGAUGUGGAGUGUGACAAUG/dC//dC//dC//dC//dC//dC//dT//dG//dC//dA//dT//dC//dT//dC//dA//dG//dT//dC//dT//dA//dT//dG//dT//dG//dC//dT//dA//dT//dG//dT//dC//dC//dA//dA//dA//dG//dG/
	Substrate-4	ATCGTCCACTCACATTGTCACACTCCACATCGCAAATGCTCGTGATCT
Split architectures II	Closed-loop spacer	/dC//dT//dT//dT//dG//dG//dA//dC//dA//dT//dA//dG//dC//dA//dC//dA//dT//dA//dG//dA//dC//dT//dG//dA//dG//dA//dC//dC//dC/CGAUGUGGAGUGUGACAAUG/dC//dC//dC//dT//dC//dT//dC//dA//dG//dT//dC//dT//dA//dT//dG//dT//dG//dC//dT//dA//dT//dG//dT//dC//dC//dA//dA//dA//dG/

circularized architecture III	Docked crRNA	/dG//dA//dG//dC//dT//dG//dT//dT//dA//dC//dT//dG//dC//dT//dT//dT//dT//dG//dC//dT//dA//dC//dC//dC//dC//dC//dC//dC/UAAUUUCUACUAAGUGUAGAUGUAGCAAAAGCAGUAACAGCUC
Split architectures IV	Docked spacer	/dC//dA//dG//dT//dC//dT//dC//dA//dG//dT//dC//dT//dA//dT//dG//dT//dG//dC//dT//dA//dT//dG//dT//dC//dT//dT//dT//dT//dT/GACAUAGCACAUAGACUGAGACUG
3’DNA	13 scaffold RNA	UAAUUUCUACUAA/dC//dC/dC//dC//dC/
	17 scaffold RNA	UAAUUUCUACUAAGUGU/dC//dC/dC//dC//dC/
	25 scaffold RNA	UAAUUUCUACUAAGUGUAGAUGACA/dC//dC/dC//dC//dC/
WT	32 spacer RNA	GUGUAGAUGACAUAGCACAUAGACUGAGACUG
	28 spacer RNA	AGAUGACAUAGCACAUAGACUGAGACUG
	20 spacer RNA	UAGCACAUAGACUGAGACUG
3’DNA(A) scaffold RNA		UAAUUUCUACUAAGUGUAGAU/dA//dA//dA//dA//dA/
3’DNA(T) scaffold RNA		UAAUUUCUACUAAGUGUAGAU/dT//dT//dT//dT//dT/
3’DNA(G) scaffold RNA		UAAUUUCUACUAAGUGUAGAU/dG//dG//dG//dG//dG/
WT spacer (C) RNA		CACAUAGCACAUAGACUGAGACUG
WT spacer (A) RNA		AACAUAGCACAUAGACUGAGACUG
WT spacer (U) RNA		UACAUAGCACAUAGACUGAGACUG
WT substrate (G) DNA		CAGTCTCAGTCTATGTGCTATGTG
WT substrate (T) DNA		CAGTCTCAGTCTATGTGCTATGTT
WT substrate (A) DNA		CAGTCTCAGTCTATGTGCTATGTA
WT substrate DNA (TS)		CCGGGAGCTCAGTCTCAGTCTATGTGCTATGTCGAAAGGGCC
WT substrate DNA (NTS)		GGCCCTTTCGACATAGCACATAGACTGAGACTGAGCTCCCGG
WT spacer RNA-1		GUAGCAAAAGCAGUAACAGCUC
WT substrate DNA-1		AGGGGATCATCAAGAGCTGTTACTGCTTTTGCTACTAAATTGGTACTGGG
WT spacer RNA-2		GAAGCAAAAGCAGAAACAGCAC
WT substrate DNA-2		AGGGGATCATCAAGTGCTGTTTCTGCTTTTGCTTCTAAATTGGTACTGGG

<table>
<tr><td colspan="2">5’Cy3 WT scaffold RNA</td><td>5’Cy3-UAAUUUCUACUAAGUGUAGAU</td></tr>
<tr><td colspan="2">5’Cy5 WT spacer (G/3) RNA</td><td>5’Cy5-GACAUAGCACAUAGACUGAGACUG</td></tr>
<tr><td colspan="2">3’Cy5 WT spacer (G/3) RNA</td><td>GACAUAGCACAUAGACUGAGACUG-3’Cy5</td></tr>
<tr><td colspan="2">5’Cy3 WT substrate (C/3) DNA</td><td>5’Cy3-CAGTCTCAGTCTATGTGCTATGTC</td></tr>
<tr><td colspan="2">5’Cy3 3’DNA(C) scaffold RNA</td><td>5’Cy3-UAAUUUCUACUAAGUGUAGAU/dC//dC//dC//dC//dC/</td></tr>
<tr><td colspan="2">5’Biotin-3’DNA Cy3 scaffold RNA</td><td>5’Biotin-UAAUUUCUACUAAGUGUAGAU/dC//dC//dC//dC//dC/-Cy3</td></tr>
<tr><td rowspan="14">3’DNA(C) scaffold RNA (loop, stem)</td><td>1dC</td><td>UAAUUUCUACUAAGUGUAGAU/dC/</td></tr>
<tr><td>2dC (2,0)</td><td>UAAUUUCUACUAAGUGUAGAU/dC//dC/</td></tr>
<tr><td>3dC (3,0)</td><td>UAAUUUCUACUAAGUGUAGAU/dC//dC//dC/</td></tr>
<tr><td>(2,5)</td><td>UAAUUUCUACUAAGUGUAGAU/dC//dC//dA//dT//dC//dT//dA/</td></tr>
<tr><td>(2,7)</td><td>UAAUUUCUACUAAGUGUAGAU/dC//dC//dA//dT//dC//dT//dA//dC//dA/</td></tr>
<tr><td>(2,9)</td><td>UAAUUUCUACUAAGUGUAGAU/dC//dC//dA//dT//dC//dT//dA//dC//dA//dC//dT/</td></tr>
<tr><td>(2,11)</td><td>UAAUUUCUACUAAGUGUAGAU/dC//dC//dA//dT//dC//dT//dA//dC//dA//dC//dT//dT//dA/</td></tr>
<tr><td>(3,5)</td><td>UAAUUUCUACUAAGUGUAGAU/dC//dC//dC//dA//dT//dC//dT//dA/</td></tr>
<tr><td>(3,7)</td><td>UAAUUUCUACUAAGUGUAGAU/dC//dC//dC//dA//dT//dC//dT//dA//dC//dA/</td></tr>
<tr><td>(3,9)</td><td>UAAUUUCUACUAAGUGUAGAU/dC//dC//dC//dA//dT//dC//dT//dA//dC//dA//dC//dT/</td></tr>
<tr><td>(3,11)</td><td>UAAUUUCUACUAAGUGUAGAU/dC//dC//dC//dA//dT//dC//dT//dA//dC//dA//dC//dT//dT//dA/</td></tr>
<tr><td>(5,5)</td><td>UAAUUUCUACUAAGUGUAGAU/dC//dC//dC//dC//dC//dA//dT//dC//dT//dA/</td></tr>
<tr><td>(5,7)</td><td>UAAUUUCUACUAAGUGUAGAU/dC//dC//dC//dC//dC//dA//dT//dC//dT//dA//dC//dA/</td></tr>
</table>

	(5,9)	UAAUUUCUACUAAGUGUAGAU/dC//dC//dC//dC//dC//dA//dT//dC//dT//dA//dC//dA//dC//dT/
	(5,11)	UAAUUUCUACUAAGUGUAGAU/dC//dC//dC//dC//dC//dA//dT//dC//dT//dA//dC//dA//dC//dT//dT//dA/
3’Biotin-2dC scaffold RNA		UAAUUUCUACUAAGUGUAGAU/dC//dC/Biotin
3’Biotin-3dC scaffold RNA		UAAUUUCUACUAAGUGUAGAU/dC//dC//dC/Biotin
3’Biotin-5dC scaffold RNA		UAAUUUCUACUAAGUGUAGAU/dC//dC//dC//dC//dC/Biotin
3’Biotin-TEG-3dC scaffold RNA		UAAUUUCUACUAAGUGUAGAU/dC//dC//dC//dC//dC/-TEG-Biotin
crRNA-1		UAAUUUCUACUAAGUGUAGAUUGAGUCGUAUUAUACUUACAC
TS-1		CTCAGGTGTAAGTATAATACGACTCACTACA
crRNA2		UAAUUUCUACUAAGUGUAGAUCGAUGUGGAGUGUGACAAUG
TS-2		CCGGGCCACTCACATTGTCACACTCCACATCGCAAACA
crRNA3		UAAUUUCUACUAAGUGUAGAUGUAGCAAAAGCAGUAACAGCUC
TS-3		GAGCTGTTACTGCTTTTGCTAC
crRNA-HPV16		UAAUUUCUACUAAGUGUAGAUUGAAGUAGAUAUGGCAGCAC
TS-HPV16		GTCATTATGTGCTGCCATATCTACTTCAGAAACTAC
NTS-HPV16		GTAGTTTCTGAAGTAGATATGGCAGCACATAATGAC
crRNA-HPV18		UAAUUUCUACUAAGUGUAGAUACAAUAUGUGCUUCUACACA
TS-HPV18		CAGGAGACTGTGTAGAAGCACATATTGTTAAATTGG
NTS-HPV18		CCAATTTAACAATATGTGCTTCTACACAGTCTCCTG
TS-HIV-1		TCTCTCTCCTTCTAGCCTCCGCTAGTCAAATAAAATGGCG
NTS-HIV-1		CGCCATTTTATTTGACTAGCGGAGGCTAGAAGGAGAGAGA
TS-TB		GCGAGGAATACAAGATTACGAACGTAAAGG

NTS-TB	CCTTTACGTTCGTAATCTTGTATTCCTCGC
HPV16 PCR PF	CCAGTATCTAAGGTTGTAAGCACG
HPV16 PCR PR	TGACCACGACCTACCTCAAC
HPV18 PCR PF	ACAATGGTGTTTGCTGGCATAATC
HPV18 PCR PR	AGTTAAAGTAATAGTACACAACTG
HPV18 (pUC57)	TTAATAAACCATATTGGTTACATAAGGCACAGGGTCATAACAATGGTGTTTGCTGGCATAATCAATTATTTGTTACTGTGGTAGATACCACTCCCAGTACCAATTTAACAATATGTGCTTCTACACAGTCTCCTGTACCTGGGCAATATGATGCTACCAAATTTAAGCAGTATAGCAGACATGTTGAGGAATATGATTTGCAGTTTATTTTTCAGTTGTGTACTATTACTTTAACTGCAGATGTTATGTCCTATATTCATAGTATGAATAGCAGTATTTTAGAGGATTGGAACTTTGGTG
HPV16 (pUC57)	TGTCTACTTGCCTCCTGTCCCAGTATCTAAGGTTGTAAGCACGGATGAATATGTTGCACGCACAAACATATATTATCATGCAGGAACATCCAGACTACTTGCAGTTGGACATCCCTATTTTCCTATTAAAAAACCTAACAATAACAAAATATTAGTTCCTAAAGTATCAGGATTACAATACAGGGTATTTAGAATACATTTACCTGACCCCAATAAGTTTGGTTTTCCTGACACCTCATTTTATAATCCAGATACACAGCGGCTGGTTTGGGCCTGTGTAGGTGTTGAGGTAGGTCGTGGTCAGCCATTA
ACTB-R	GAGCGCGGCTACAGCTT
ACTB-F	TCCTTAATGTCACGCACGATTT
TB-Reporter	Texas Red-CCCCCC-BHQ2
FB-Reporter	FAM-TTATT-BHQ1

*Orange represents the PAM sequence.

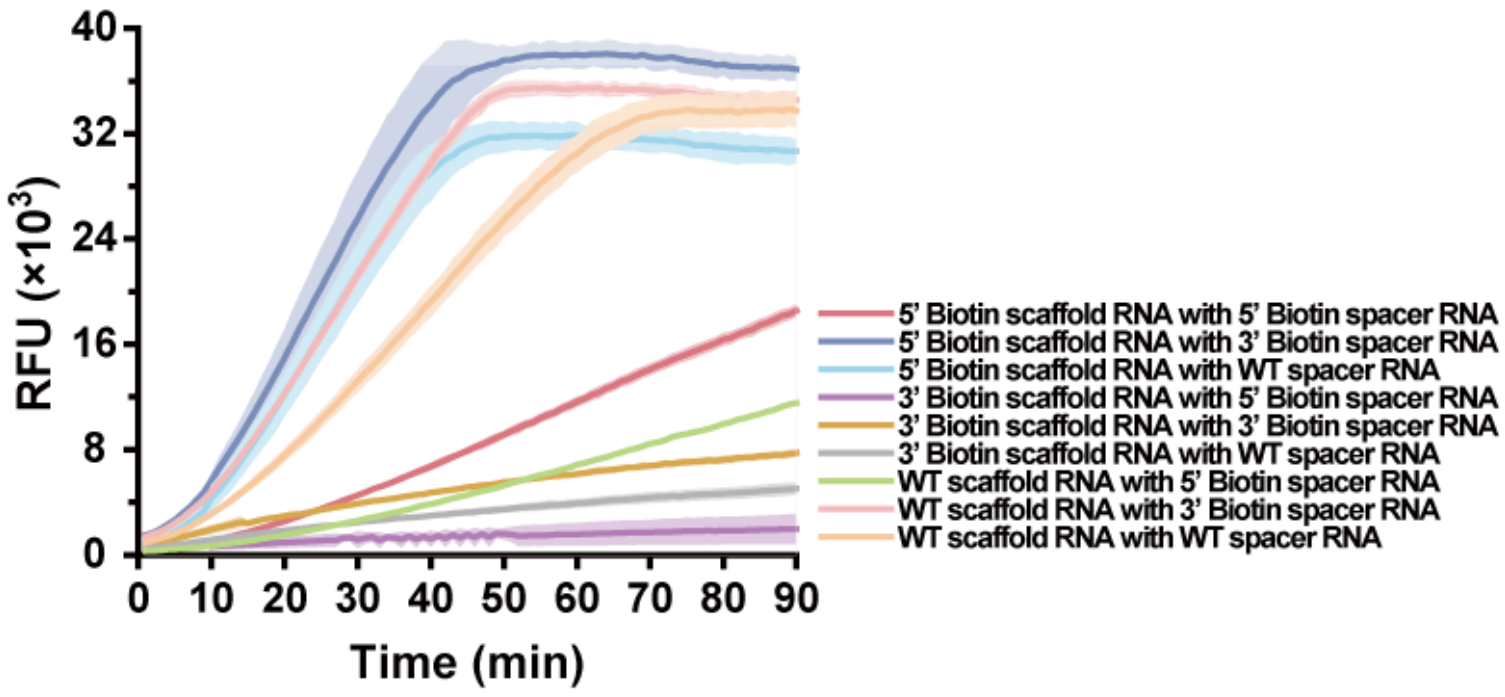

Figure S1. Monitoring real-time fluorescence signal to assess the *trans*-cleavage activity of Cas12a on scaffold RNA and spacer RNA fragments with or without 5'/3' end biotin modifications. Data are expressed as mean ± SD (n = 3).

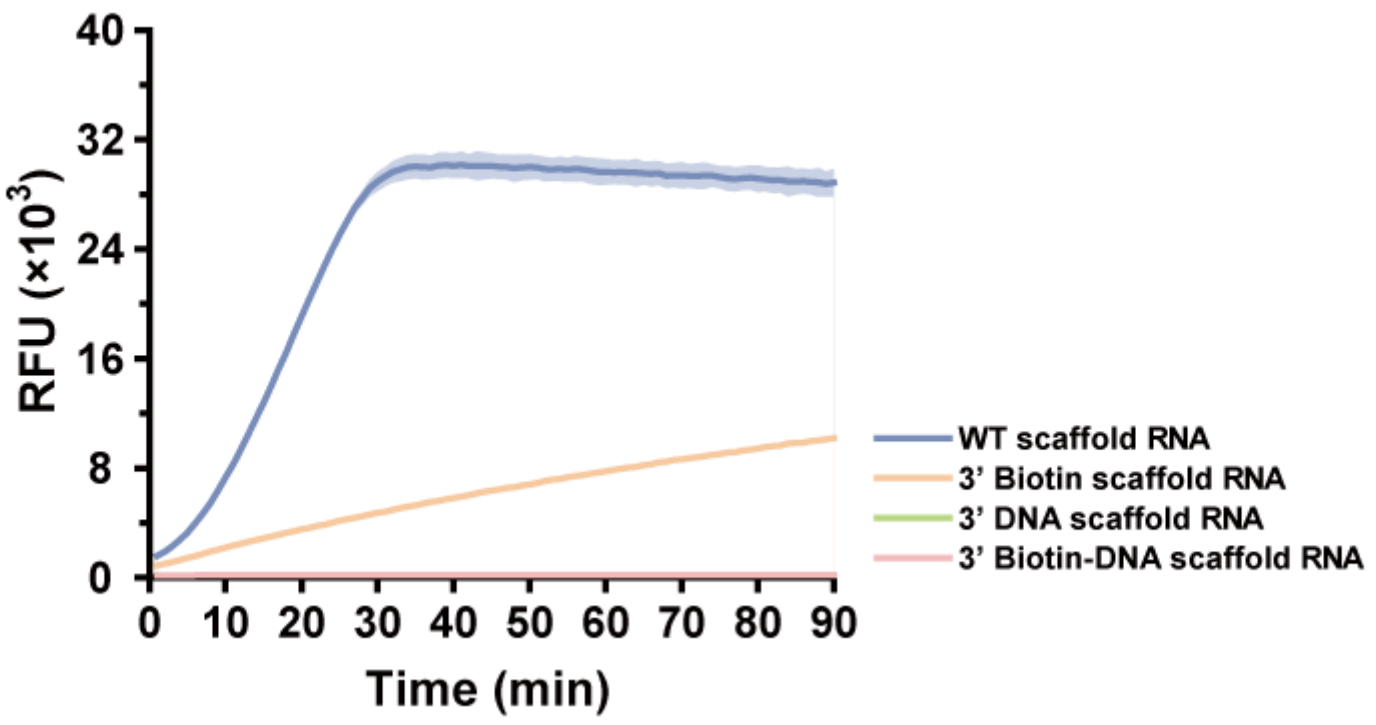

Figure S2. Monitoring real-time fluorescence to assess the *trans*-cleavage activity of Cas12a on scaffold RNA with 3' end modifications: Biotin only, DNA only, and Biotin-DNA conjugate. Data are expressed as mean ± SD (n = 3).

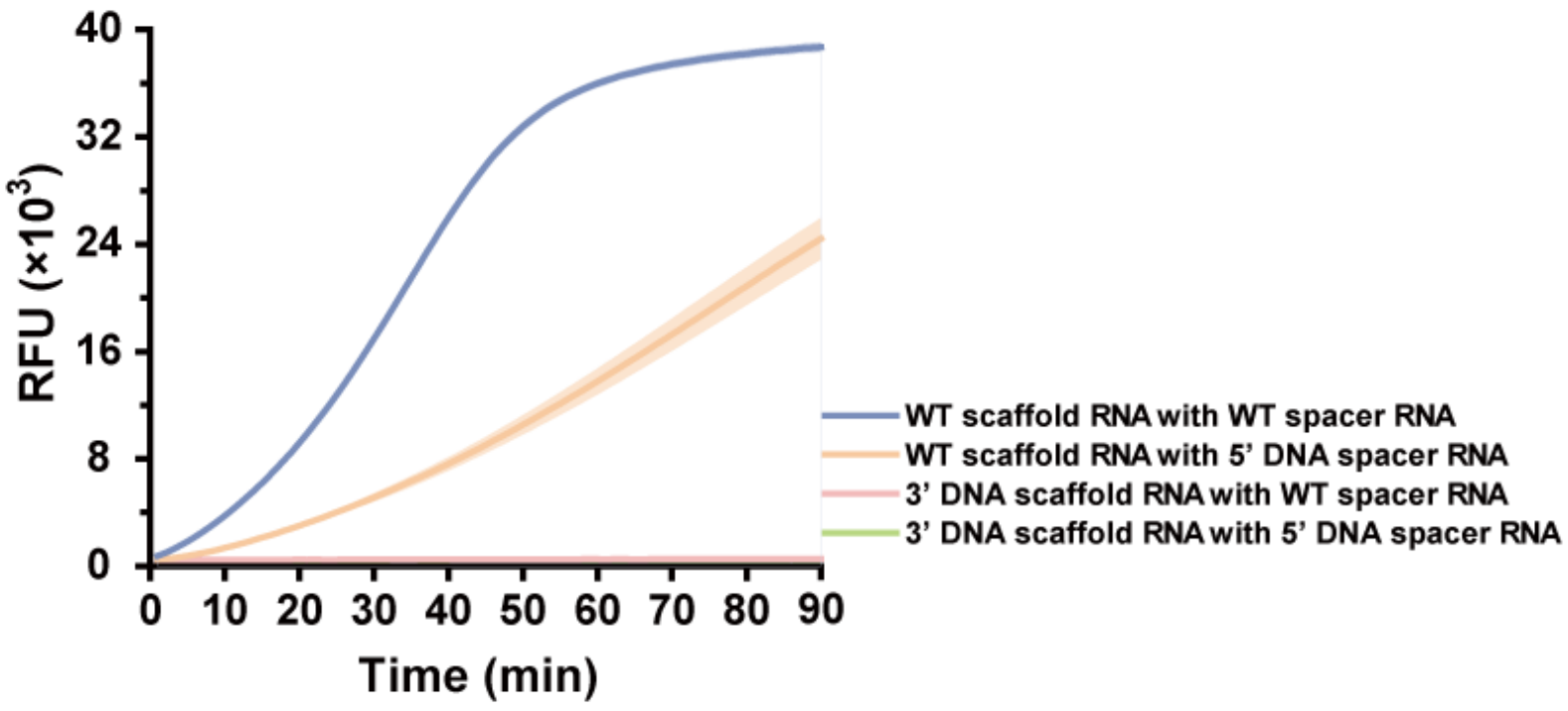

Figure S3. Monitoring real-time fluorescence to assess the *trans*-cleavage activity of Cas12a on scaffold RNA and spacer RNA fragments with or without DNA extension. Data are expressed as mean ± SD (n = 3).

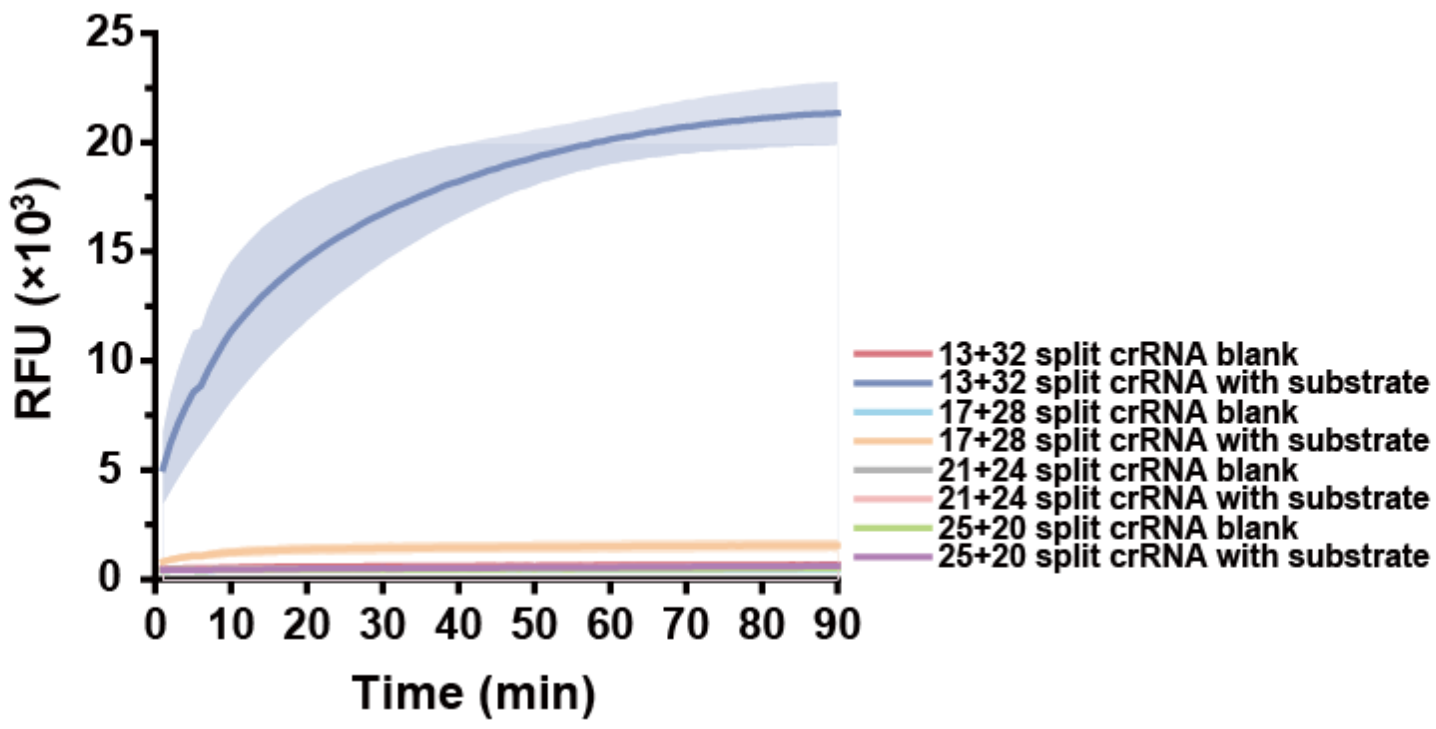

Figure S4. Monitoring real-time fluorescence to assess the *trans*-cleavage activity of Cas12a on split crRNA variants at different division/extension segmentation sites. Data are expressed as mean ± SD (n = 3).

Building upon the confirmed high efficacy of DNA extension of scaffold RNA in suppressing Cas12a, we further investigated whether synergistic effects could be achieved by combining the DNA extension with engineered conformational changes. To examine this combinatorial effect, we assembled a set of crRNA variants with specific topological characteristics (including circular and split designs) and systematically investigated how the integration of local chemical properties and global structural features together affects Cas12a activity (Figure S5a). We designed topological structures I/II by incorporating cleavable single-stranded DNA (ssDNA) and its complementary DNA strand to both ends of the standard crRNA or split spacer RNA, aiming to hinder the dynamic reorganization of crRNA during the assembly of the R-loop by strategically imposing global restrictions to decrease conformational freedom (Figure S5b, k). On the other hand, we designed a complex mimicking an intramolecular pre-hybridized state (Structures III/IV) by covalently linking the 5' end of the crRNA or spacer to the 3' end of complementary target DNA via a cleavable ssDNA (Figure S5c, l). This design was intended to competitively inhibit the binding of crRNA to free genomic targets through a steric occupancy mechanism. Whereas all data show that the engineered topology and flexible ssDNA component offered a suitable framework for its R-loop formation, causing insufficient suppression of Cas12a's activity (Figure S5i, j, s, t). In contrast, scaffold RNA with DNA extension (Structure V, also called 3'D-SR) achieved complete suppression (Figure S5m, s, t). Overall, with its optimal background suppression, 3'D-SR is poised to become a key switch module for cascade amplification via relief of inhibition.

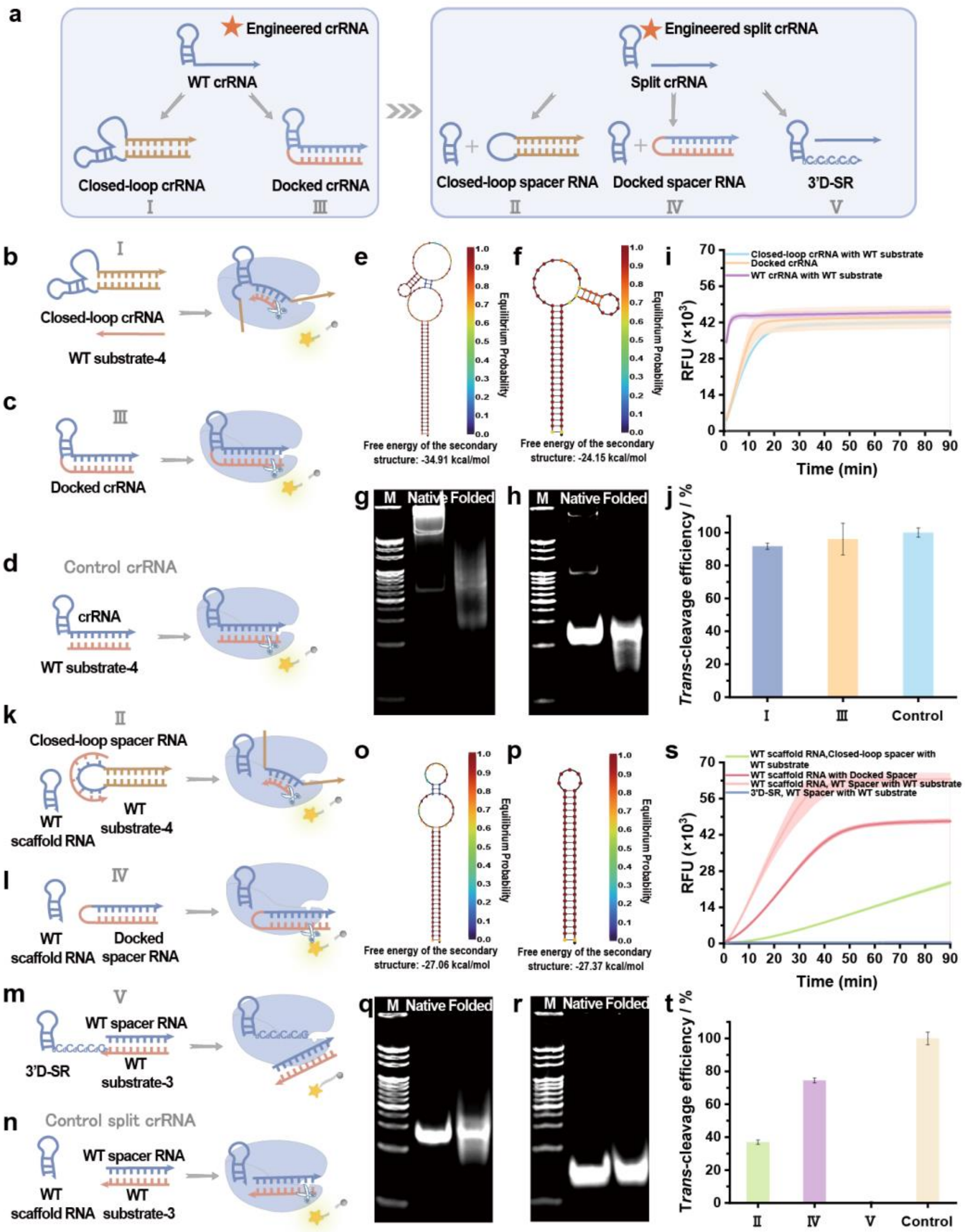

Figure S5. (a) Schematic illustration of different engineered topological structures of crRNA. (b-d, k-n) Schematic representations of binding modalities for different topological crRNA-substrate complexes. (e, f, o, p) NUPACK-predicted secondary structures for circularized and split architectures. Color coding (right color bar) is used to visualize structural formation probability, where higher color intensity correlates with a greater chance of structural folding. Calculated free energy values quantify the thermodynamic stability of each complex, with more negative values reflecting greater conformational stability. (g, h, q, r) 12% PAGE analysis of crRNA variants with distinct topological conformations, corresponding to the constructs illustrated in Figure S5e, f, o, p. A DNA marker was used as the reference. The concentrations of the above strands were set at 1 μM. (i, j, s, t) Assessment of Cas12a *trans*-cleavage activity on different topological crRNA architectures by real-time

fluorescence monitoring and final intensity recording at 90 min (The *trans*-cleavage efficiency of WT crRNA or WT split crRNA was defined as 100%). Data are expressed as mean ± SD (n = 3).

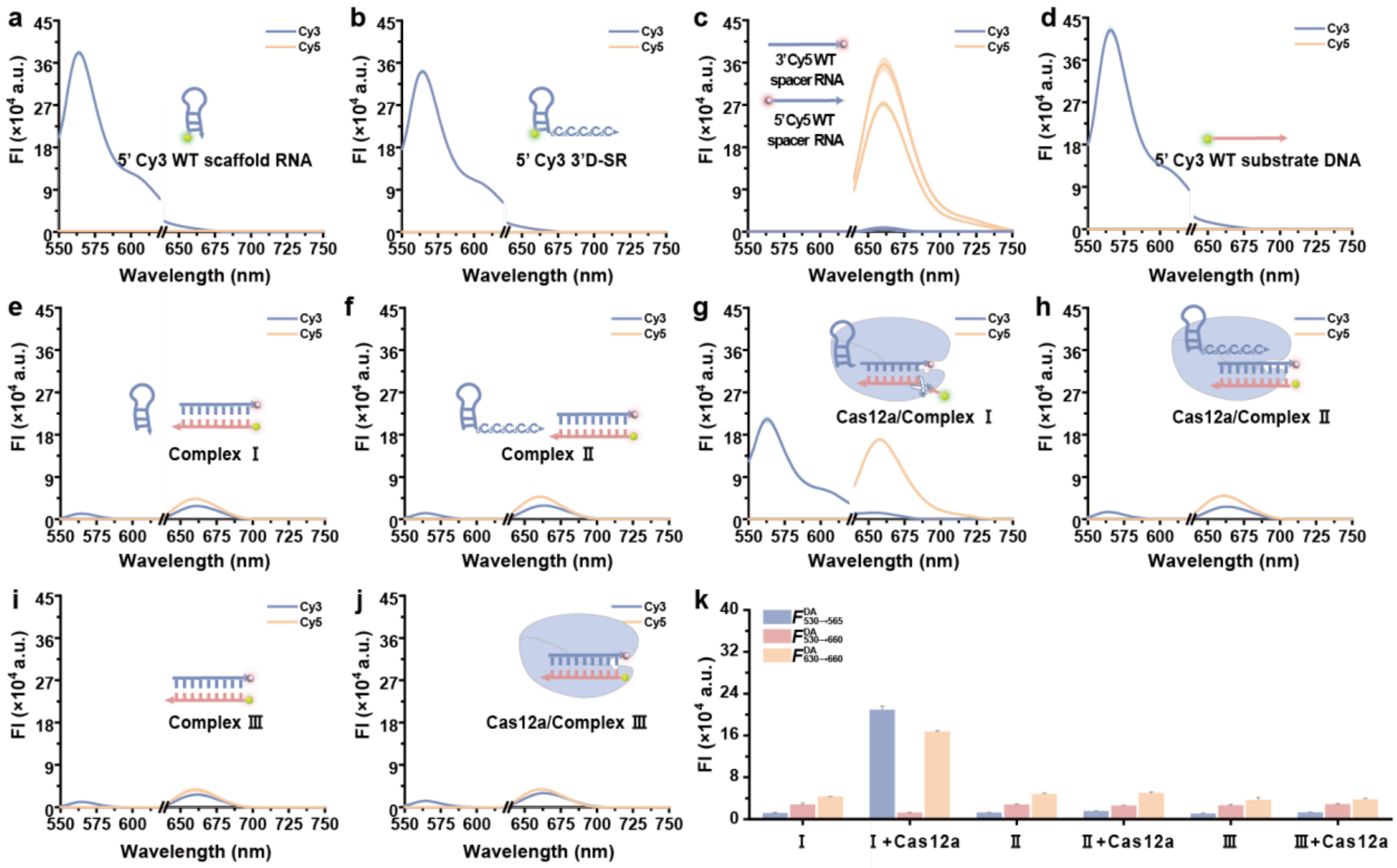

Figure S6. Fluorescence spectral profiles of various molecular systems (nucleic acids and their protein-bound complexes) acquired in the Cy3 and Cy5 channels: (a) 5’ Cy3 WT scaffold RNA; (b) 5’ Cy3 3’D-SR; (c) 3’ Cy5 WT spacer RNA and 5’ Cy5 WT spacer RNA; (d) 5’ Cy3 WT substrate DNA; (e) Complex I; (f) Complex II; (g) Cas12a with Complex I; (h) Cas12a with Complex II; (i) 3’ Cy5 WT spacer and 5’ Cy3 WT substrate, called Complex III; (j) Cas12a with Complex III; (k) Comparison of fluorescence signals across different channels for complex I, II, and III before and after Cas12a addition. Data are expressed as mean ± SD (n = 3).

To interrogate the distance changes of scaffold RNA/spacer RNA during the construction process, we labeled Cy5 at the 3' terminus of the spacer RNA and Cy3 at the 5' end of the scaffold RNA. Under protein-free conditions, no significant FRET was detected irrespective of scaffold RNA with DNA extension, indicating that there is a large spatial separation between the spacer and the scaffold RNA in the base state, which hinders effective energy transfer. The introduction of Cas12a did not cause FRET. Furthermore, the subsequent addition of the substrate strand failed to elevate FRET levels above background levels (Figure S7a-k). And although gel shift assays confirmed that Cas12a retained partial binding capacity for the 3'D-SR. However, distinct substrate cleavage bands were observed exclusively with the WT scaffold RNA system, whereas the 3'D-SR system was entirely cleavage inactive (Figure S7l-m). These collective findings demonstrate that although 3'D-SR could bind to Cas12a, the DNA extension of scaffold RNA results in a loss of catalytic function. To discount any proximity-induced artifacts from the labeling scheme, we engineered an alternative construct with Cy3 at the 5' end of spacer RNA (Figure S8). The consistent outcome provides additional evidence that the 3'D-SR may impose critical steric obstruction, leading to the inhibition of *cis*-activity of Cas12a.

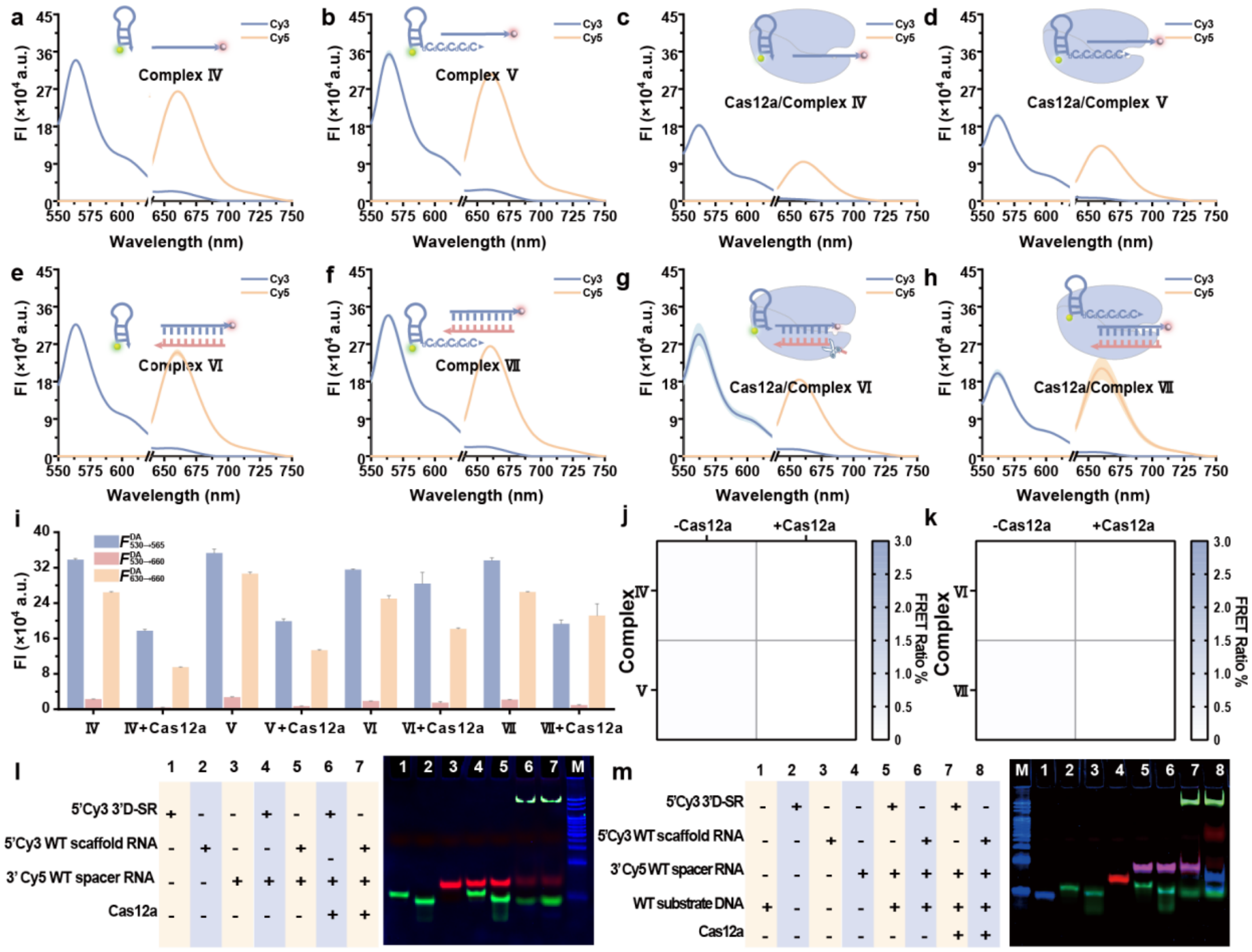

Figure S7. Fluorescence spectral profiles of various molecular systems acquired in

the Cy3 and Cy5 channels: (a) 5’ Cy3 WT scaffold RNA with 3’ Cy5 WT spacer RNA, called Complex IV; (b) 5’ Cy3 3’D-SR with 3’ Cy5 WT spacer RNA, called Complex V; (c) Cas12a with Complex IV; (d) Cas12a with Complex V; (e) 5’ Cy3 WT scaffold RNA with 3’ Cy5 WT spacer RNA and WT substrate DNA, called Complex VI; (f) 5’ Cy3 3’D-SR with 3’ Cy5 WT spacer RNA and WT substrate DNA, called Complex VII; (g) Cas12a with Complex VI; (h) Cas12a with Complex VII; (i) Comparison of fluorescence signals across different channels for complexes IV, V, VI, and VII before and after Cas12a addition; (j, k) the results of the FRET ratio of the interaction of different molecular structures with or without Cas12a; (l, m) Electrophoretic mobility shift assay of the interaction of different molecular structures with or without Cas12a. Data are expressed as mean ± SD (n = 3).

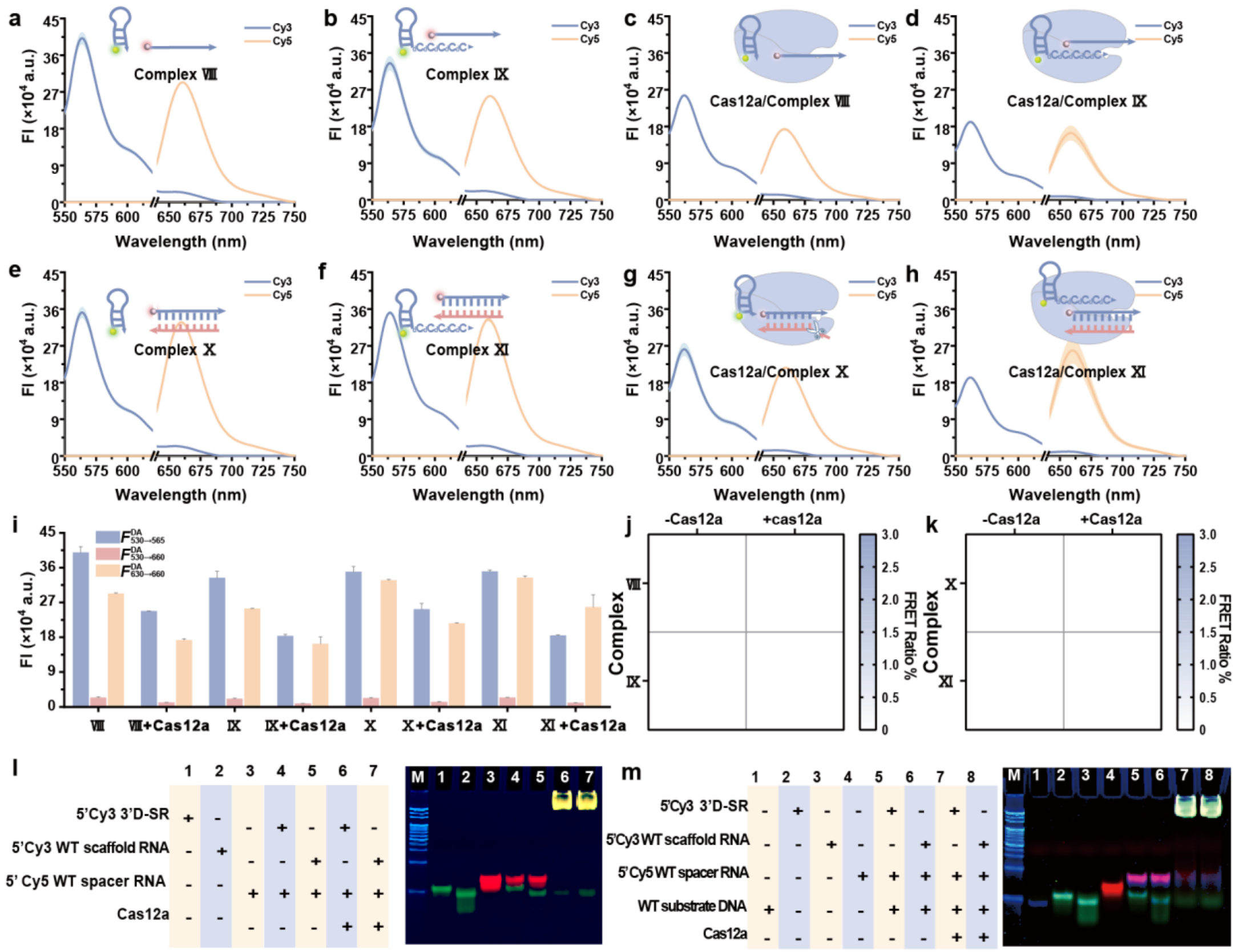

Figure S8. Fluorescence spectral profiles of various molecular systems acquired in the Cy3 and Cy5 channels: (a) 5’ Cy3 WT scaffold RNA with 5’ Cy5 WT spacer RNA, called Complex VIII; (b) 5’ Cy3 3’D-SR with 5’ Cy5 WT spacer RNA, called Complex IX; (c) Cas12a with Complex VIII; (d) Cas12a with Complex IX; (e) 5’ Cy3 WT scaffold RNA with 5’ Cy5 WT spacer RNA and WT substrate DNA, called Complex X; (f) 5’ Cy3 3’D-SR with 5’ Cy5 WT spacer RNA and WT substrate DNA, called Complex XI; (g) Cas12a with Complex X; (h) Cas12a with Complex XI; (i)

Comparison of fluorescence signals across different channels for substrates VIII, IX, X and XI before and after Cas12a addition; (j, k) the results of FRET ratio of the interaction of different molecular structures with or without Cas12a; (l, m) Electrophoretic mobility shift assay of the interaction of different molecular structures with or without LbCas12a. Data are expressed as mean ± SD (n = 3).

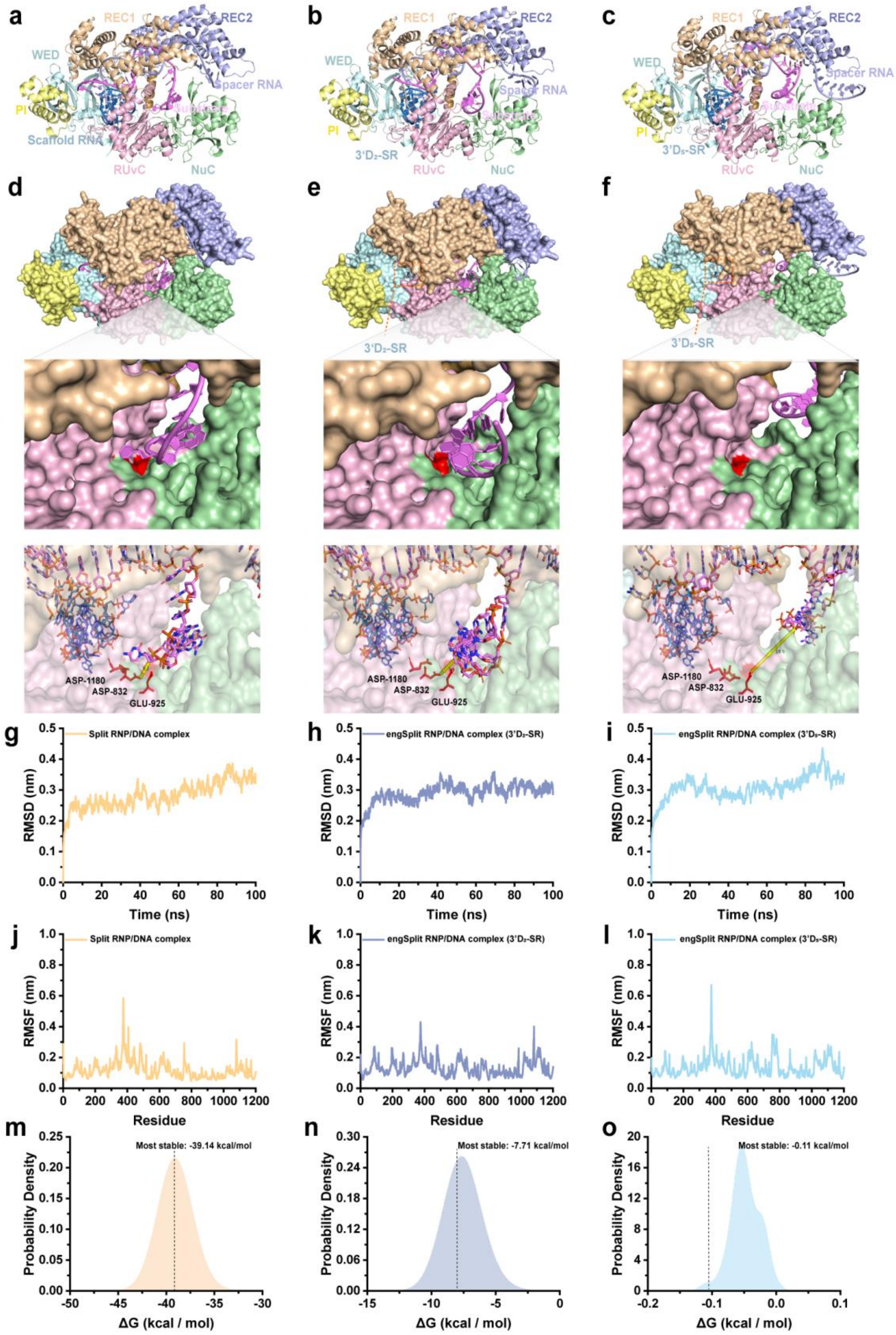

Figure S9. Exploration of the molecular mechanism underlying the inhibition of Cas12a system functionality. (a-f) Molecular docking simulation results of the Split RNP/DNA complex (a,d), engSplit RNP/DNA complex with 3’D_2-SR (b, e), and engSplit RNP/DNA complex with 3’D_5-SR (c, f). (g-i) Time evolution of RMSD of the above complex. (j-l) RMSF of the above complex. (m-o) Comparison of binding free energy of the above complex.

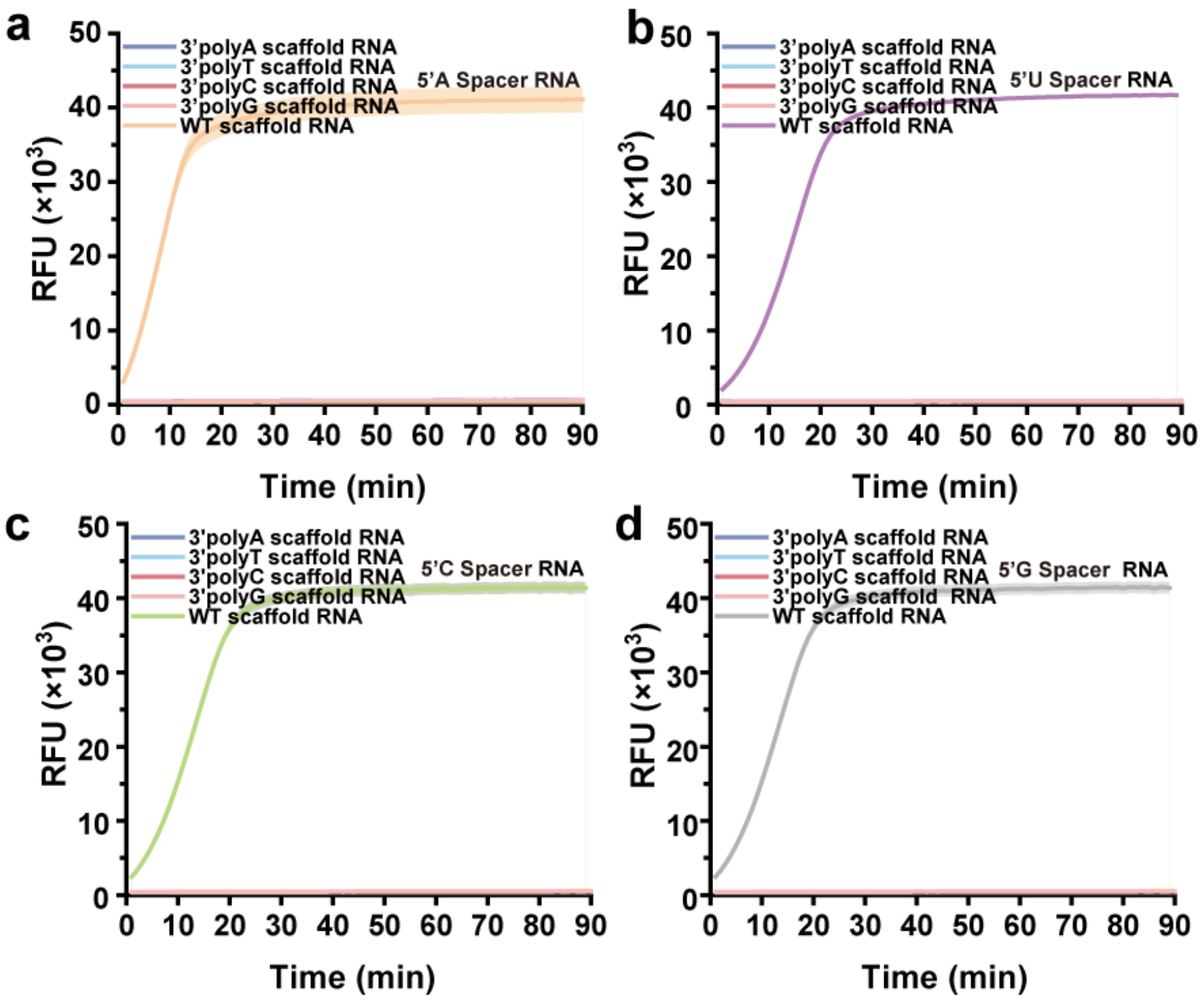

Figure S10. Monitoring real-time fluorescence to assess the *trans*-cleavage activity of Cas12a on different bases of 3'D-SR and spacer RNA. Data are expressed as mean ± SD (n = 3).

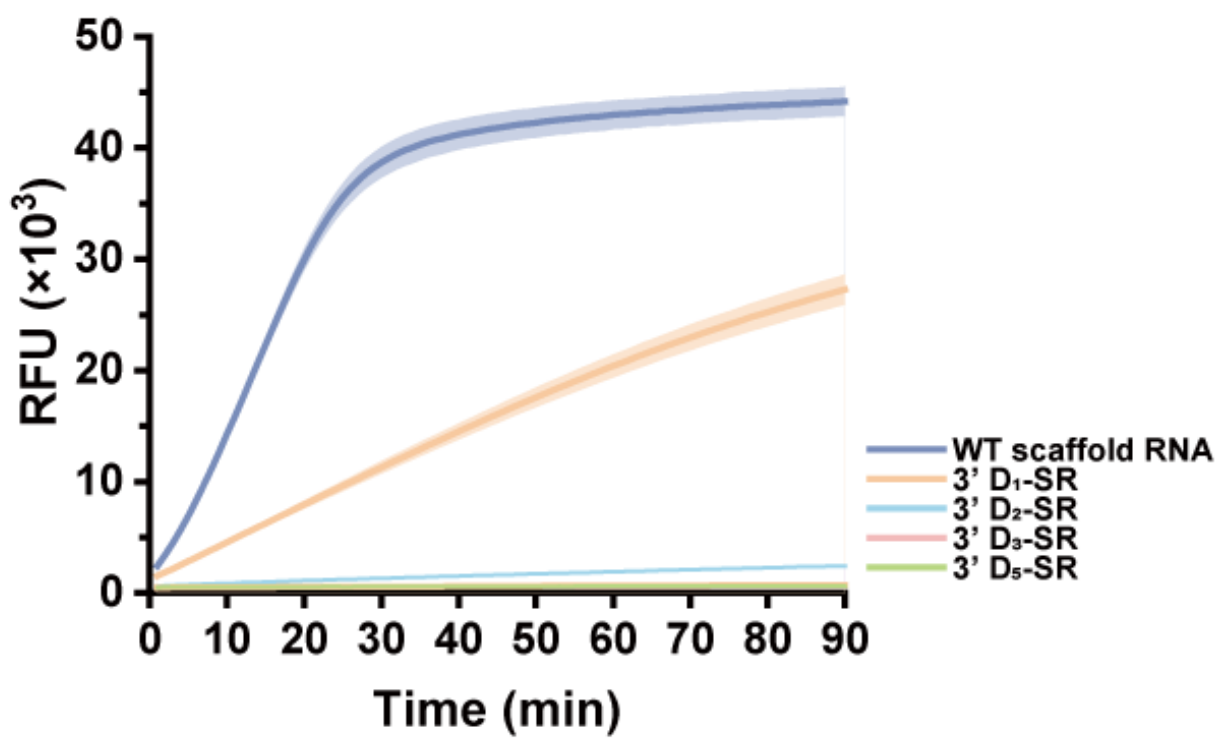

Figure S11. Monitoring real-time fluorescence to assess the *trans*-cleavage activity of Cas12a on 3'D-SR with different lengths of DNA. Data are expressed as mean ± SD (n = 3).

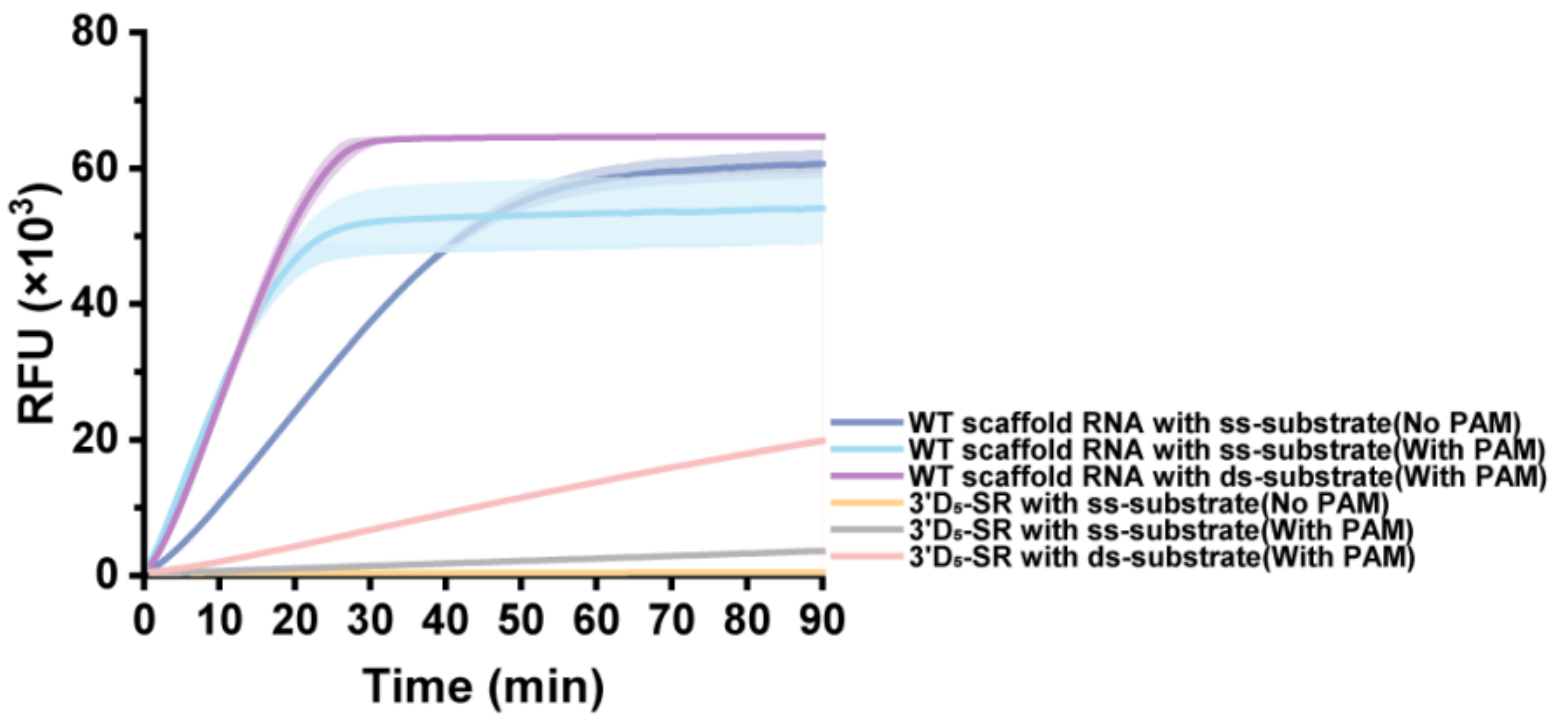

Figure S12. Monitoring real-time fluorescence to assess the *trans*-cleavage activity of Cas12a on ssDNA or dsDNA substrate. Data are expressed as mean ± SD (n = 3).

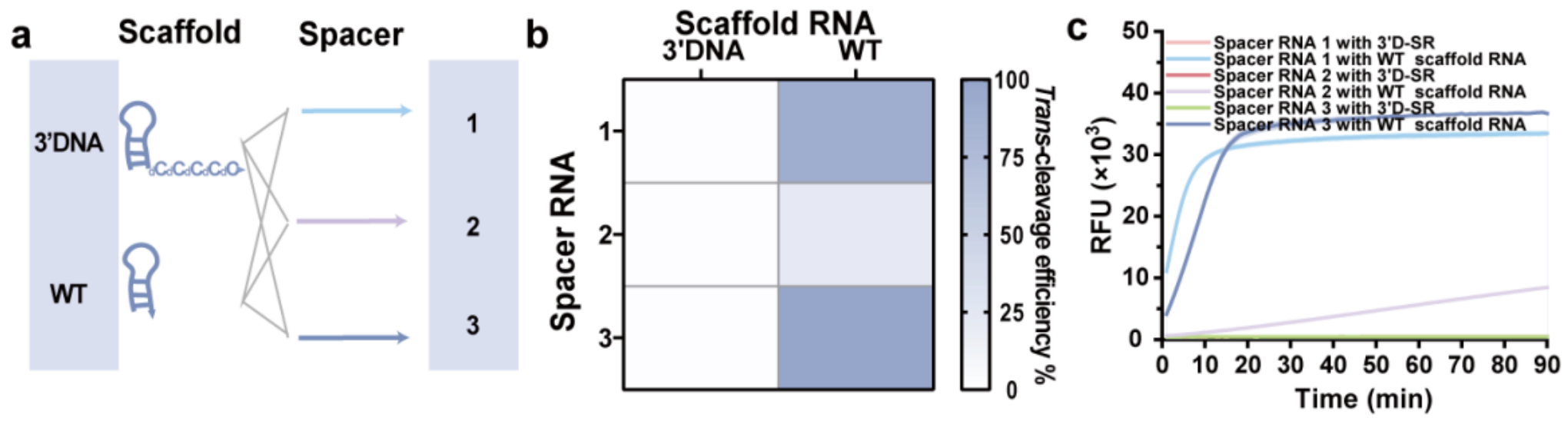

Figure S13. (a,b) Schematic illustration of the inhibitory efficacy of different spacer sequences with the WT scaffold RNA or 3'D-SR. And the *trans*-cleavage efficiency of these substrates on the activity of Cas12a (WT split crRNA 3's *trans*-cleavage efficiency was defined as 100%). (c) Monitoring real-time fluorescence to assess the *trans*-cleavage activity of Cas12a. Data are expressed as mean ± SD (n = 3).

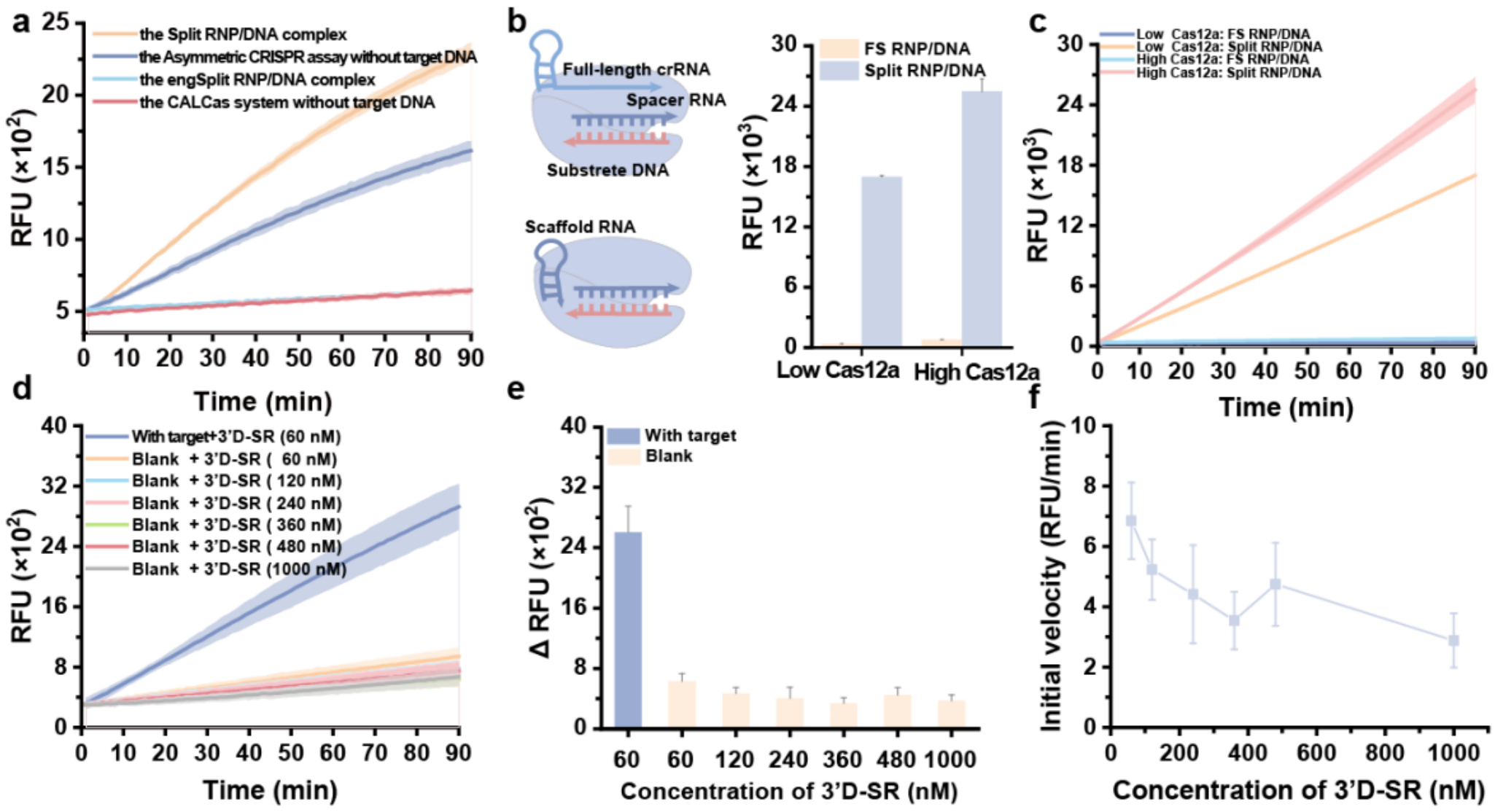

Figure S14. (a) Monitoring the real-time fluorescence signal in Figure 4c to assess the *trans*-cleavage activity of Cas12a. (b) Comparative analysis of the *trans*-cleavage activity of Cas12a mediated by full-length crRNA versus scaffold RNA (Cas12a assembled with full-length crRNA, spacer RNA and substrate, referred to as the FS RNP/DNA complex). Comparison of endpoint RFU generated by the FS RNP/DNA and the Split RNP/DNA complexes at both low and high Cas12a concentrations. (c) Monitoring the real-time fluorescence signal of the above reaction in Figure S14b. Monitoring the real-time fluorescence signal (d) and fluorescence signal intensities (e) of 3'D-SR at various concentrations in the absence of target DNA. (f) Scatter plot showing the correlation between 3'D-SR concentration and the corresponding reaction rate. Data are expressed as mean ± SD (n = 3).

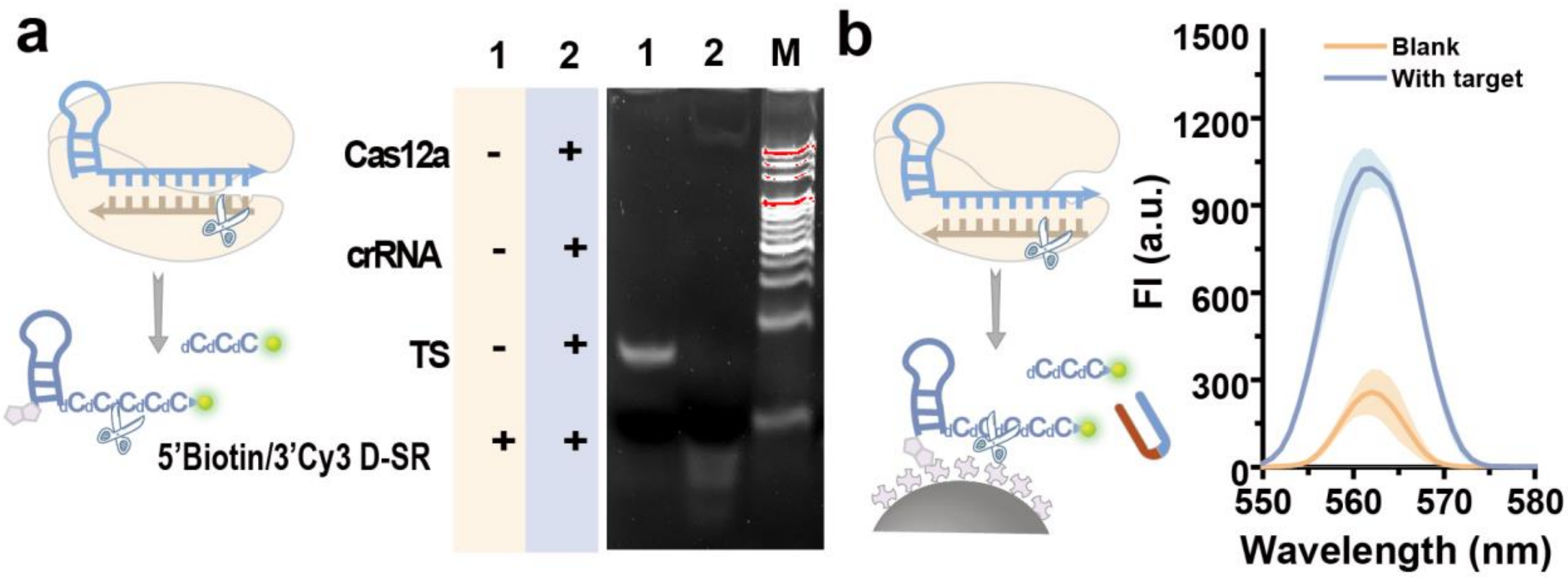

Figure S15. PAGE (a) and magnetic bead capture experiments (b) were employed to investigate the Cas12a-mediated release reaction. Data are expressed as mean ± SD (n = 3).

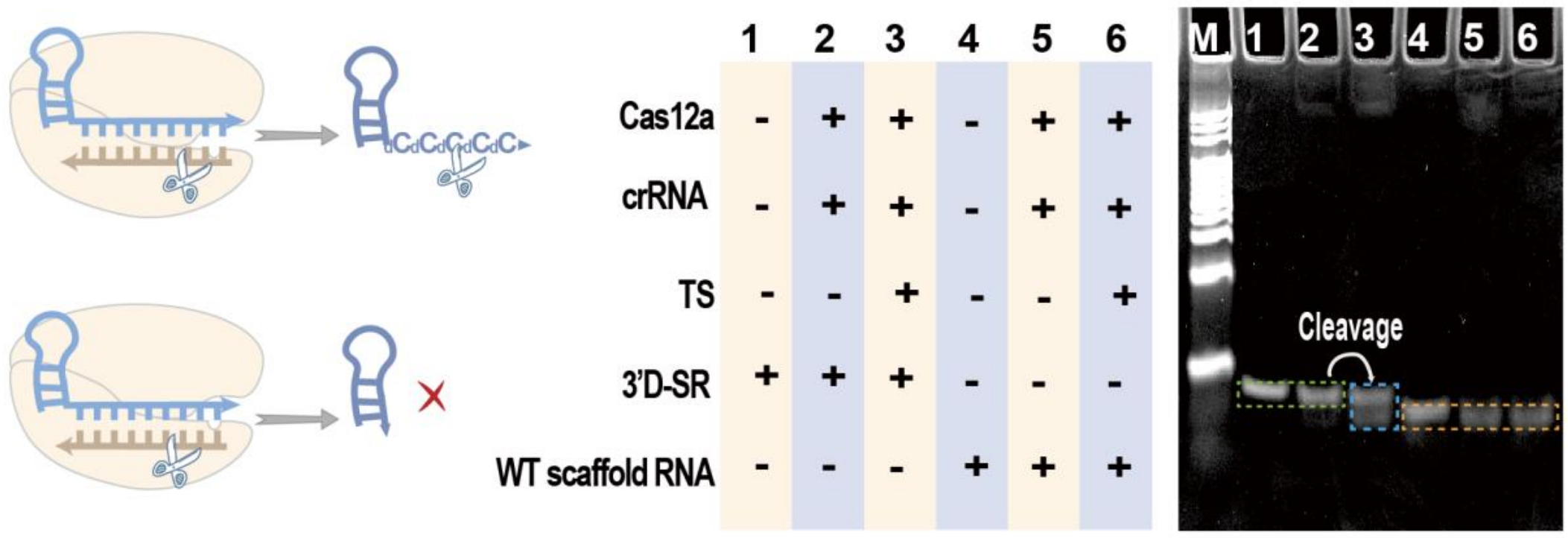

Figure S16. PAGE validation of Cas12a cleavage activity preference for ssDNA over RNA backbones. Lane 1: 3'D-SR; Lane 2: Cas12a, crRNA with 3'D-SR; Lane 3: Cas12a, crRNA, target with 3'D-SR; Lane 4: WT scaffold RNA; Lane 5: Cas12a, crRNA with WT scaffold RNA; Lane 6: Cas12a, crRNA, target with WT scaffold RNA.

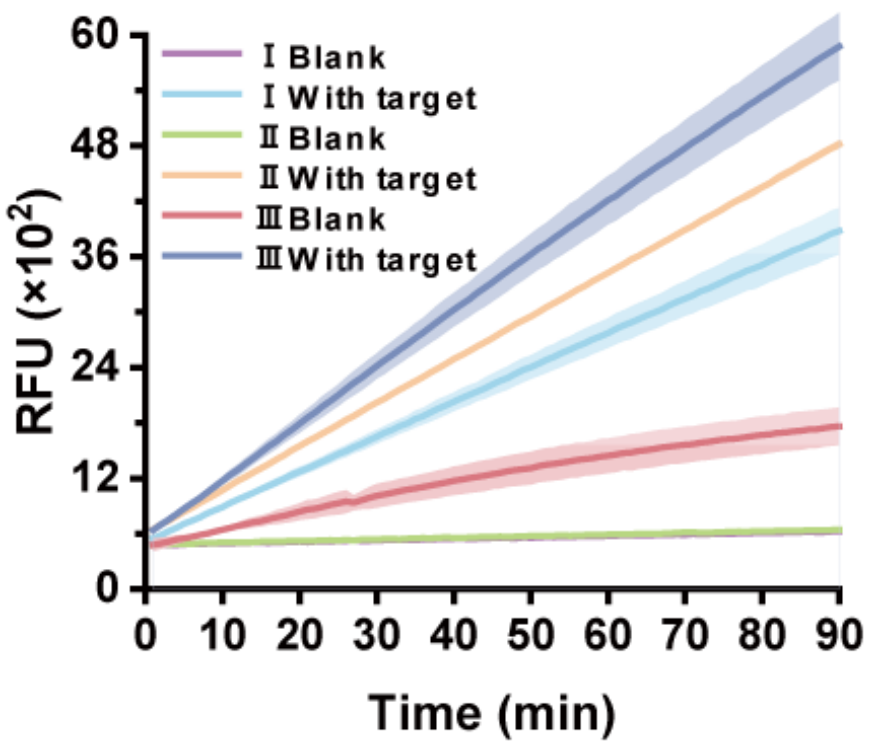

Figure S17. Monitoring real-time fluorescence to compare the performance of the single Cas12a system, Asymmetric CRISPR assay and CALCas system. Data are expressed as mean ± SD (n = 3).

Optimization of experimental parameters

As shown in Figure S18, the change in RFU (ΔRFU) increased as the ratio of upstream Cas12a/crRNA complex rose, reaching its peak at a 1:1 ratio. Therefore, the 1:1 proportion was chosen for subsequent experiments. Next, the concentration of the upstream Cas12a/crRNA complex was further optimized. The ΔRFU gradually increased and reached a peak value at 10 nM. While a slight decrease was observed if the concentration of the upstream Cas12a/crRNA complex was higher than 10 nM, indicating that the high-concentration complex might hinder Cas12a's activity. Thus, 10 nM was selected as the optimum concentration. Then, the concentration of the WT spacer RNA/substrate was examined from 20 to 0.5 nM. The ΔRFU intensified as the concentration of WT spacer/substrate increased from 20 to 10 nM. After that, the ΔRFU gradually declined. Therefore, to balance the reaction signal and background signal, 10 nM was used for further investigations. Next, the concentration of the downstream Cas12a/3'D-SR was further optimized. The concentration progressively increased and attained a peak value at 60 nM. A significant decrease was observed if the concentration of downstream Cas12a/3'D-SR fell below 60 nM. Thus, 60 nM was chosen as the optimum concentration.

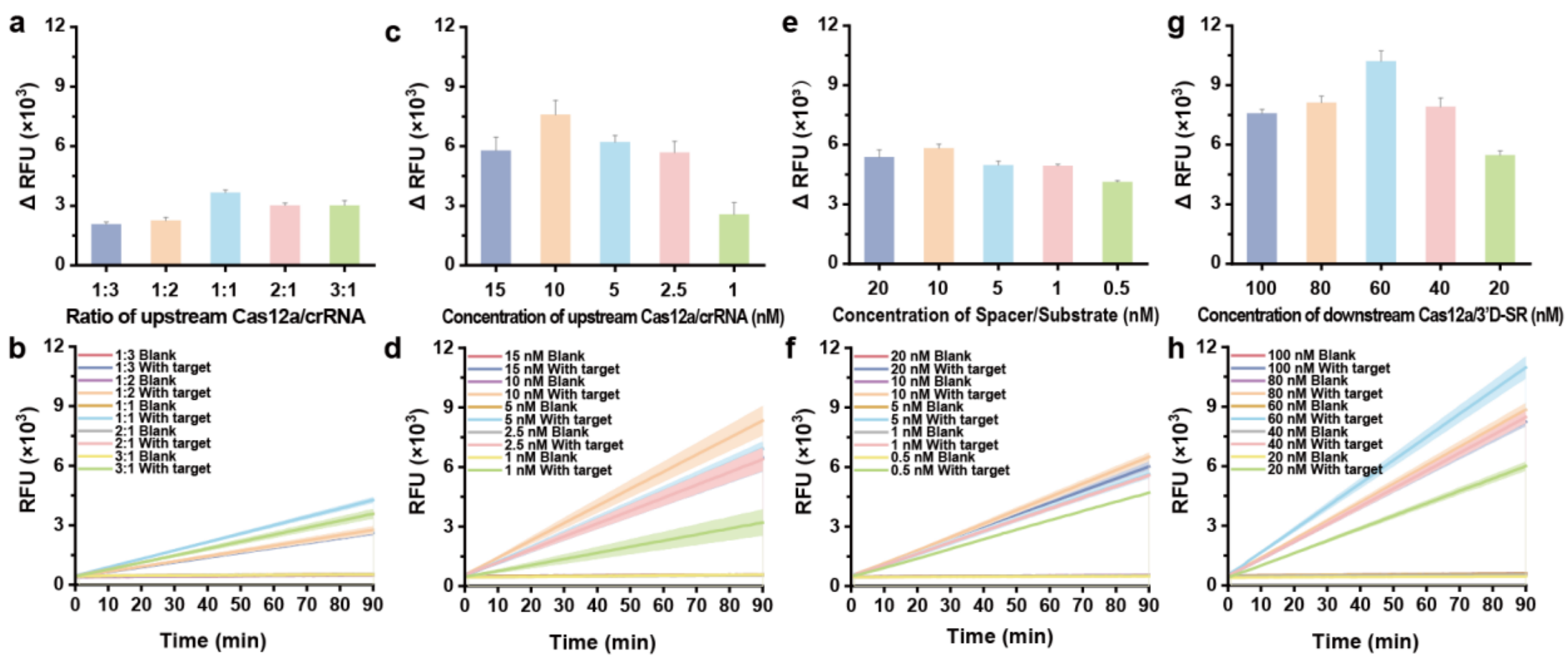

Figure S18. Fluorescence response under optimization of key parameters. Data are represented as ΔRFU and real-time fluorescence kinetics. (a,b) Optimization of the ratio of the upstream Cas12a/crRNA complex. (c,d) Optimization of the concentration of the upstream Cas12a/crRNA complex. (e,f) Optimization of the concentration of the WT spacer RNA and substrate. (g,h) Optimization of the concentration of the downstream Cas12a/3'D-SR. Data are expressed as mean ± SD (n = 3).

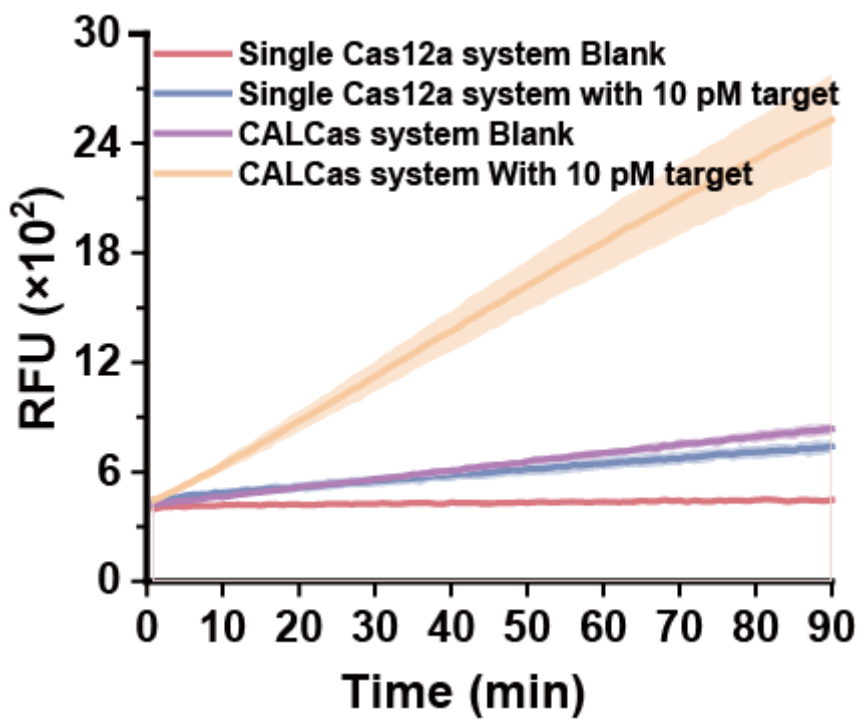

Figure S19. Monitoring real-time fluorescence to compare the performance of the single Cas12a system and the CALCas system. Data are expressed as mean ± SD (n = 3).

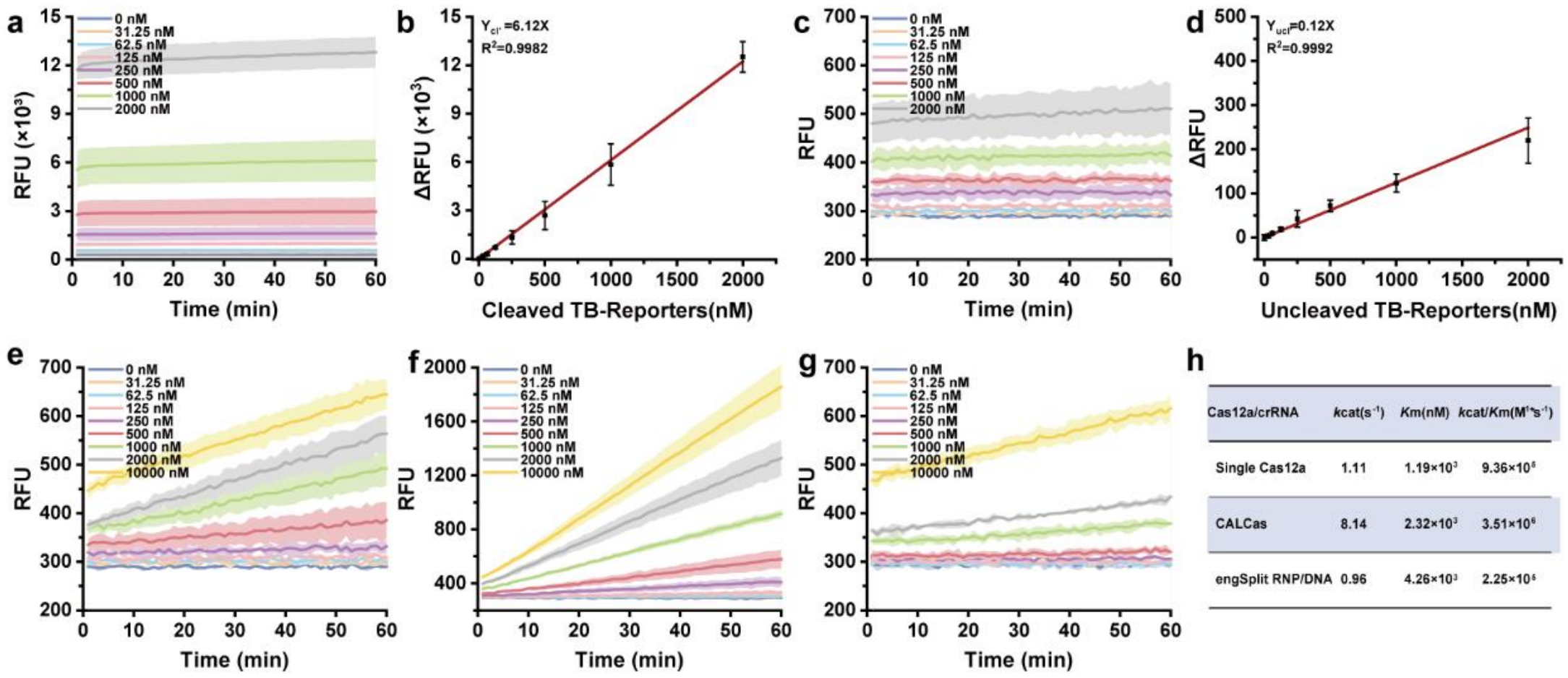

Figure S20. Michaelis-Menten kinetic analysis. (a) Real-time fluorescence signals generated by cutting 10 nM target strand with different concentrations of TB-Reporter. (b) Subtracted fluorescence F_{cl} plotted against the concentration of cleaved reporters C_{cl}. (c) Real-time fluorescence signals generated without the target strand at different concentrations of TB-Reporter. (d) Subtracted fluorescence F_{ucl} plotted against the concentration of uncleaved reporters C_{ucl}. (e) Real-time fluorescence signals for varying concentrations of reporters, ranging from 0 nM to 10 μM, with the single Cas12a system. (f) Real-time fluorescence signals for varying concentrations of reporters, ranging from 0 nM to 10 μM, with the CALCas system. (g) Real-time fluorescence signals for varying concentrations of reporters, ranging from 0 nM to 10 μM, with the engSplit RNP/DNA complex. (h) Comparison of parameters in Michaelis–Menten kinetics for different Cas12a systems. Data are expressed as mean ± SD (n = 3).

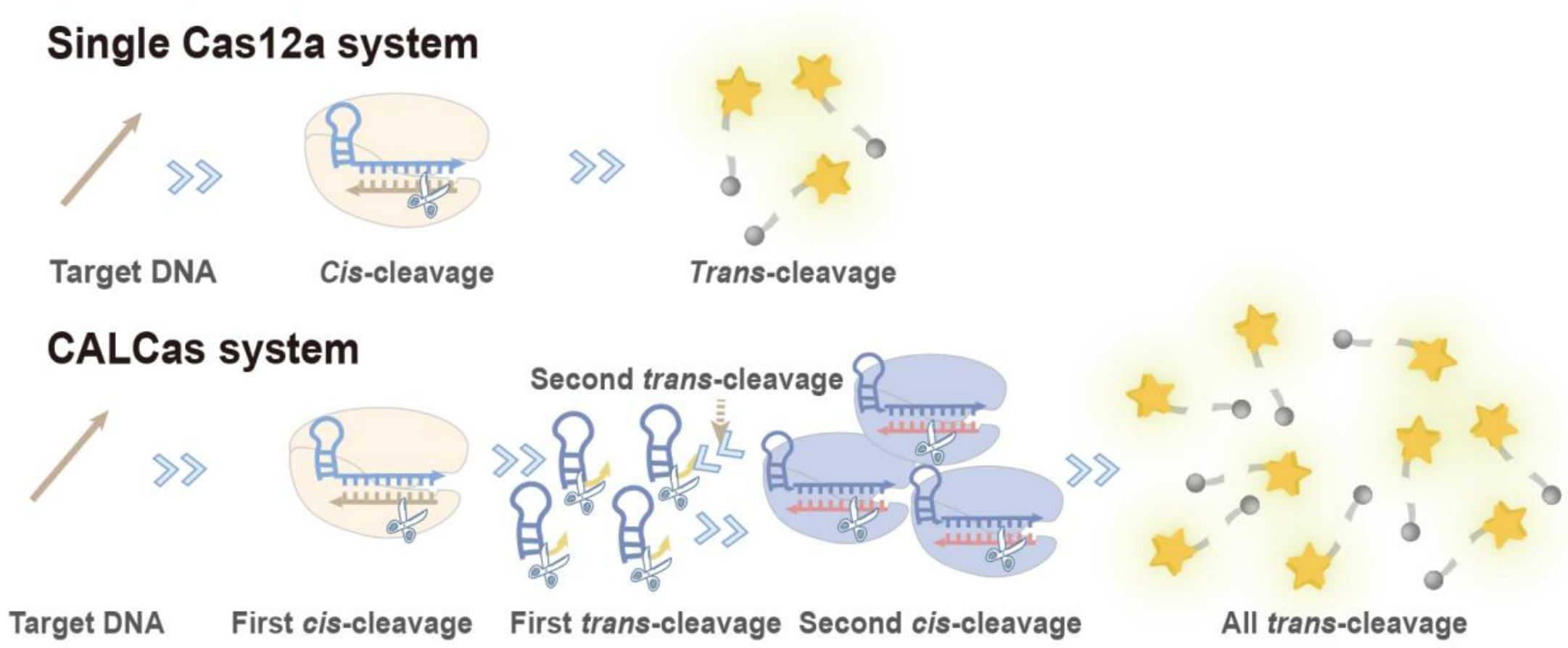

Figure S21. Schematics comparing the reaction patterns of the single Cas12a system (top) and the CALCas system (bottom).

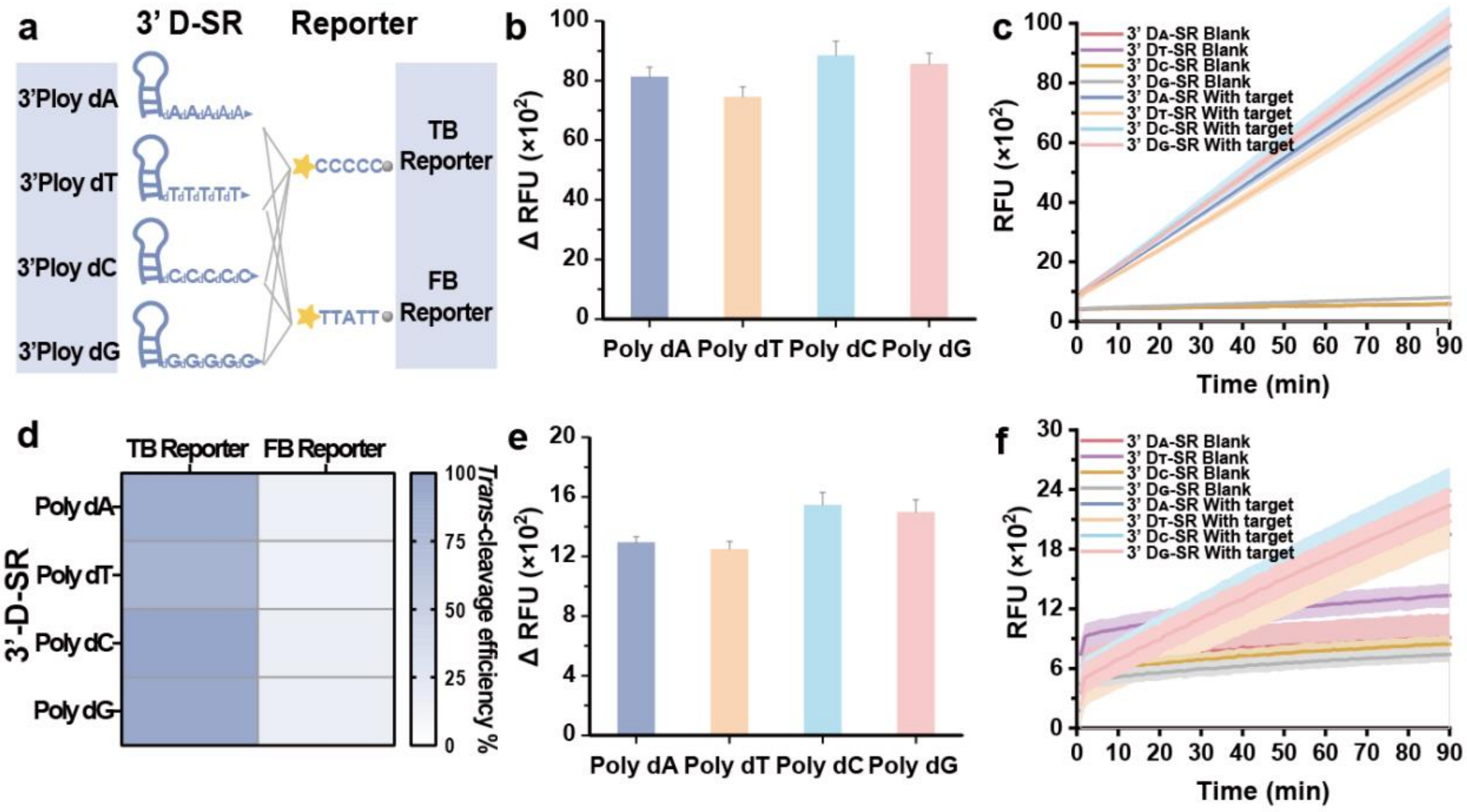

Figure S22. Optimization of the base of 3'D-SR and reporter. (a) Schematic illustration of different DNA bases of 3'D-SR and Reporter. (b, c) Fluorescence response with different DNA bases of 3'D-SR under TB-Reporter. Data are represented as ΔRFU and real-time fluorescence kinetics. (e, f) Fluorescence response with different DNA bases of 3'D-SR under FB-Reporter. Data are represented as ΔRFU and real-time fluorescence kinetics. (d) The *trans*-cleavage efficiency of Cas12a activity under different DNA bases of 3'D-SR and Reporter (The *trans*-cleavage efficiency of the group with 3'D_C-SR and TB-reporter was defined as 100%). Data are expressed as mean ± SD (n = 3).

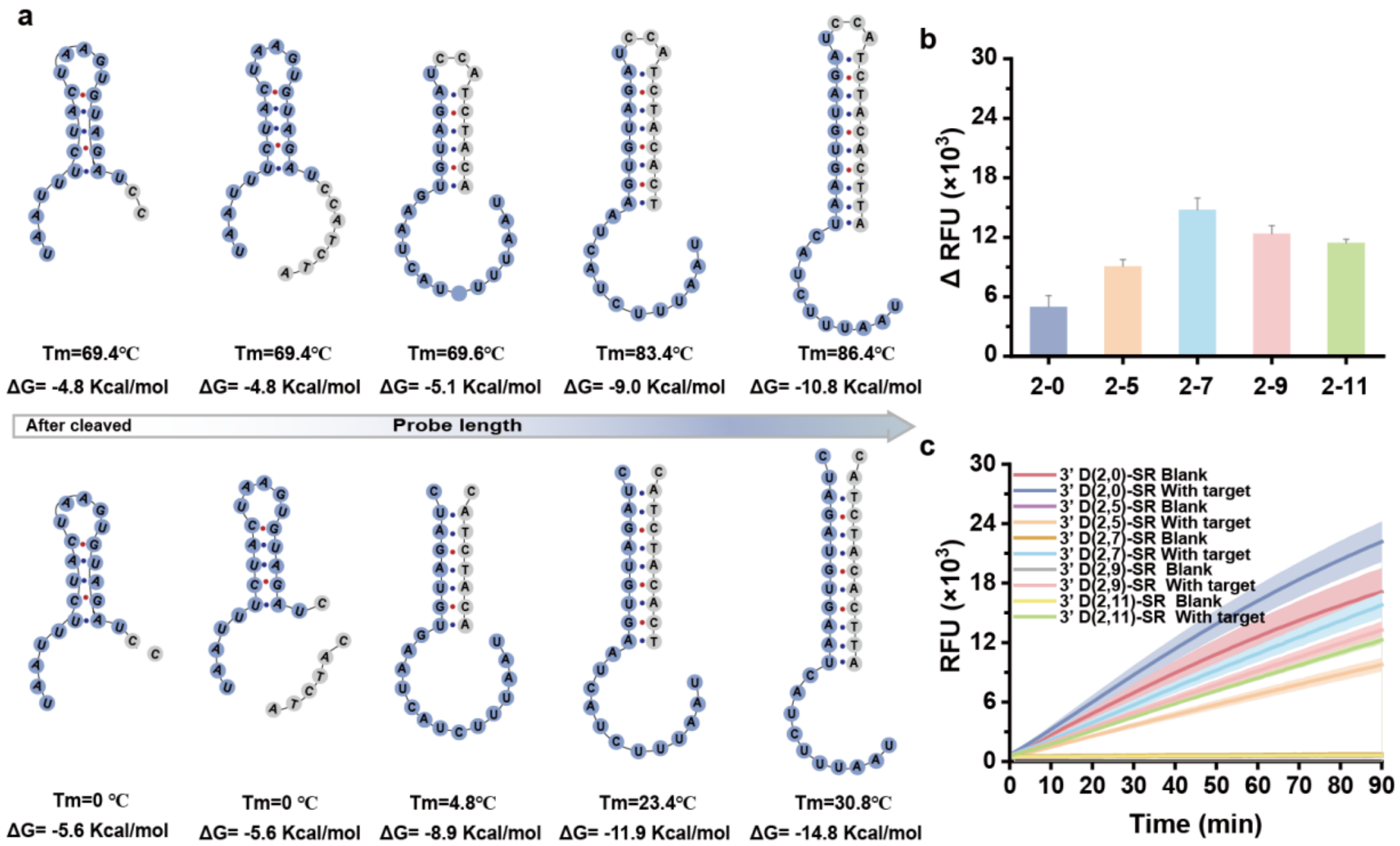

Figure S23. Schematic illustration of the hairpin 3'D-SR with two loops and variable stem lengths (a). Data are shown as ΔRFU in (b) and real-time fluorescence kinetics in (c). Gray and blue represent DNA and RNA, respectively. Data are expressed as mean ± SD (n = 3).

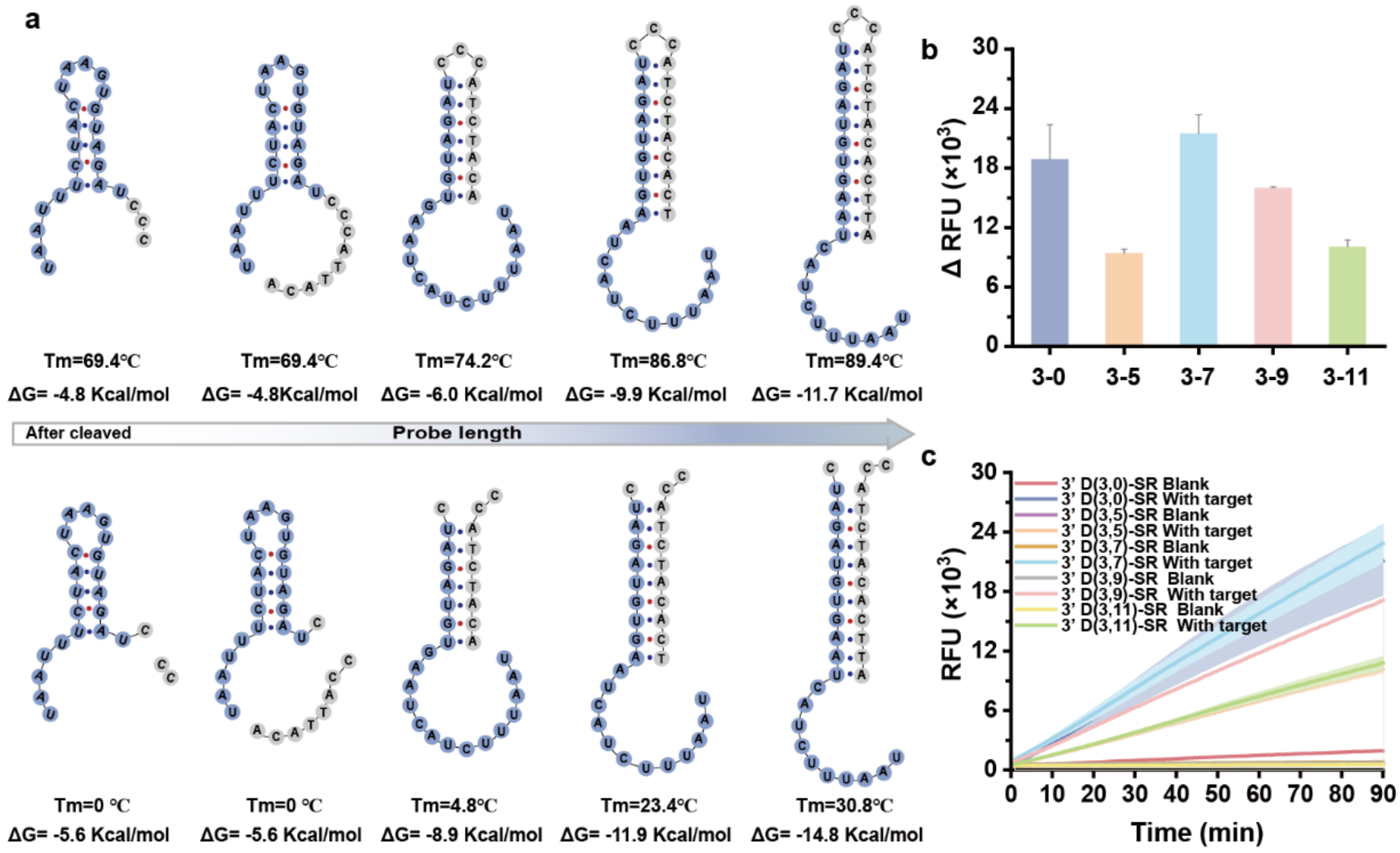

Figure S24. Schematic illustration of the hairpin 3'D-SR with three loops and variable stem lengths (a). Data are shown as ΔRFU in (b) and real-time fluorescence kinetics in (c). Gray and blue represent DNA and RNA, respectively. Data are expressed as mean ± SD (n = 3).

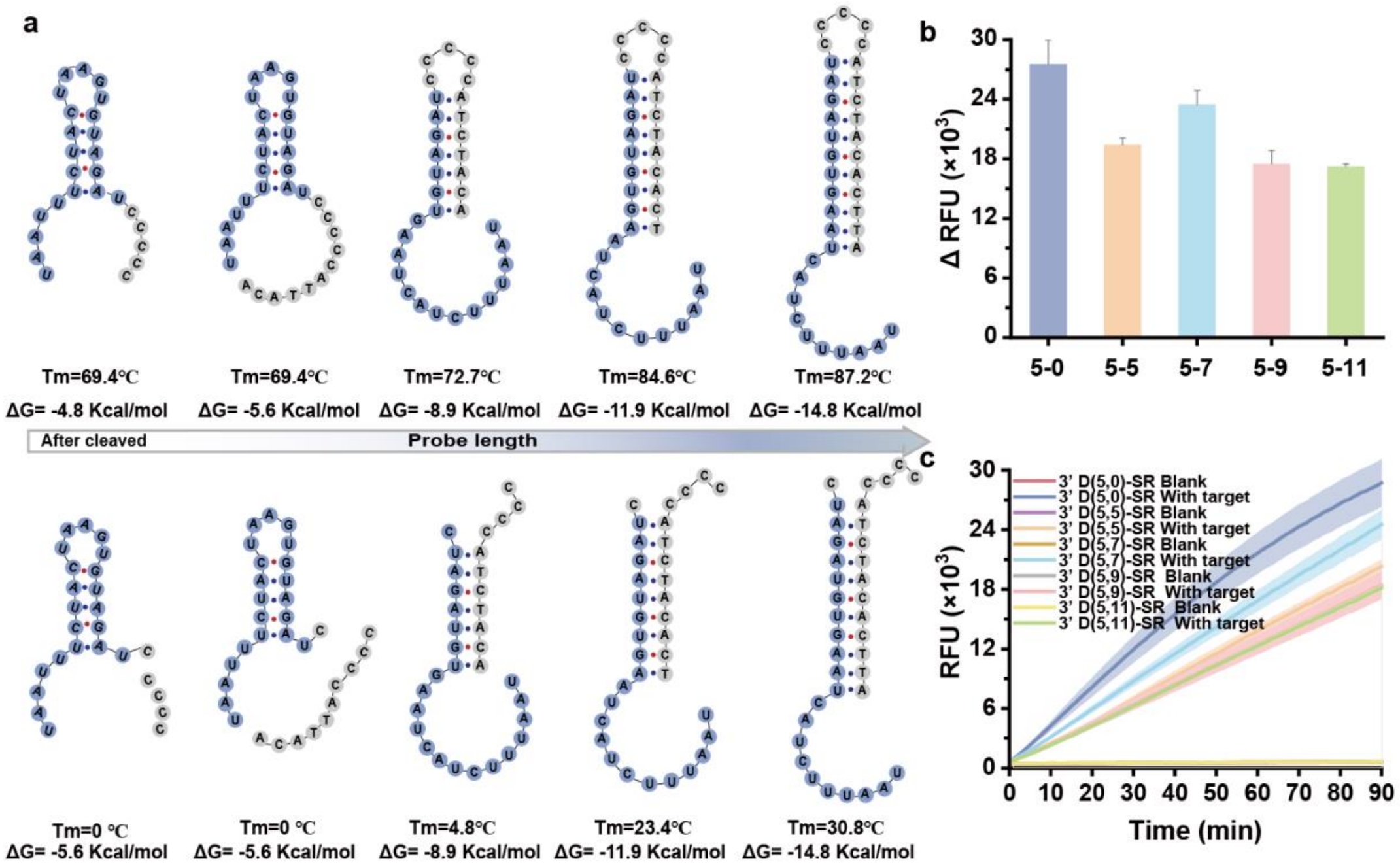

Figure S25. Schematic illustration of the hairpin 3’D-SR with five loops and variable stem lengths (a). Data are shown as ΔRFU in (b) and real-time fluorescence kinetics in (c). Gray and blue represent DNA and RNA, respectively. Data are expressed as mean ± SD (n = 3).

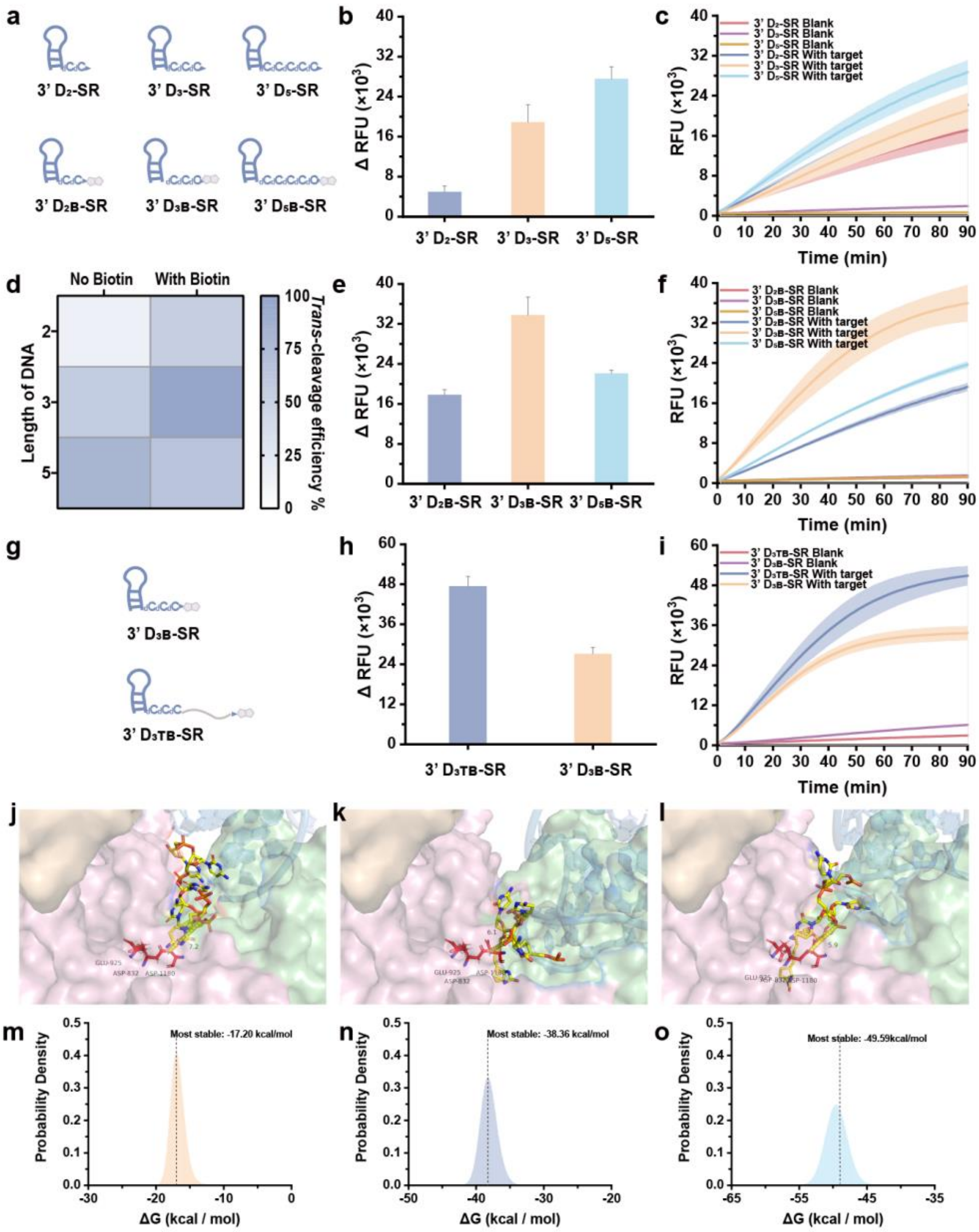

Figure S26. Optimization of 3’D-SR. (a) Schematic illustration of different-length DNA base and biotin modifications of scaffold RNA. (b, c) Fluorescence response with different lengths of DNA of 3’D-SR. (e, f) Fluorescence response with different lengths of DNA of 3’D$_B$-SR. (d) The *trans*-cleavage efficiency of Cas12a under different lengths of DNA base and modifications of scaffold RNA (The combination with the highest cleavage efficiency was set as 100%). (g-i) Schematic illustration of TEG and biotin modification of the 3’D-SR. D (j-l) Molecular docking simulation results of 3’D$_5$-SR (j), 3’D$_{3B}$-SR (k), and 3’D$_{3TB}$-SR (l). (m-o) Comparison of binding free energy of the above complexes. Data are expressed as mean ± SD (n = 3).

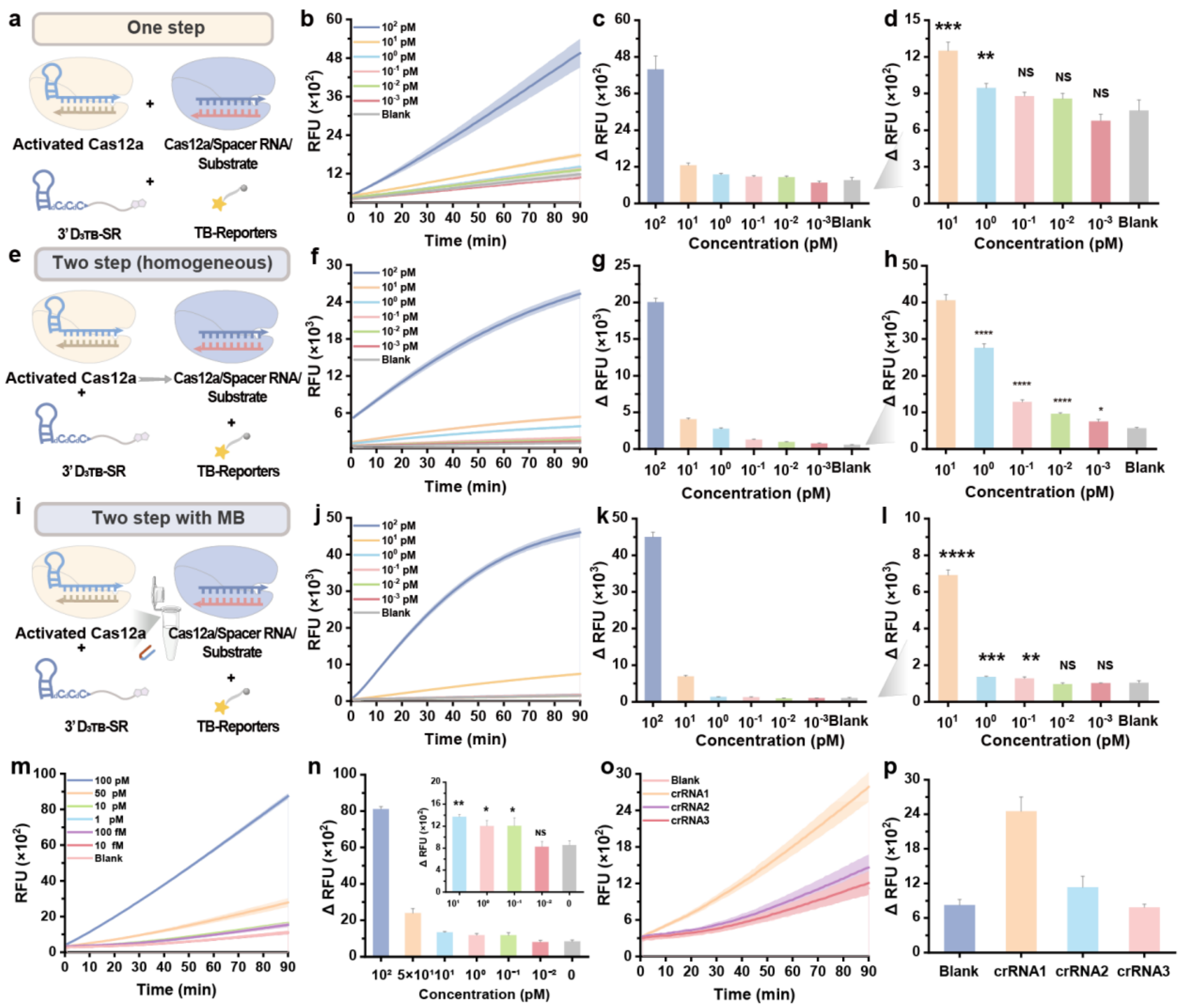

Figure S27. (a) Schematic diagram of the one-step CRISPR system. (b-d) Real-time fluorescence kinetics and ΔRFU of the target DNA from 100 pM to 1 fM, using the one-step method. (e) Schematic diagram of the two-step homogeneous method. (f-h) Real-time fluorescence kinetics and ΔRFU of the target DNA from 100 pM to 1 fM, using the two-step homogeneous method. (i) Schematic diagram of the two-step magnetic bead-assisted method. (j-l) Real-time fluorescence kinetics and ΔRFU of the target DNA from 100 pM to 1 fM, using the two-step magnetic bead-assisted method. (m, n) Real-time fluorescence kinetics and ΔRFU of the target DNA from 100 pM to 10 fM using the Asymmetric CRISPR systems. (o, p) Real-time fluorescence kinetics and ΔRFU of the Asymmetric CRISPR system for detecting different targets. Data are expressed as mean ± SD (n = 3). The levels of significance relative to the control group were assessed by one-way ANOVA with Dunnett's post-hoc test and are denoted as ****, $p < 0.0001$; ***, $p < 0.001$; **, $p < 0.01$; *, $p < 0.05$; NS, not significant.

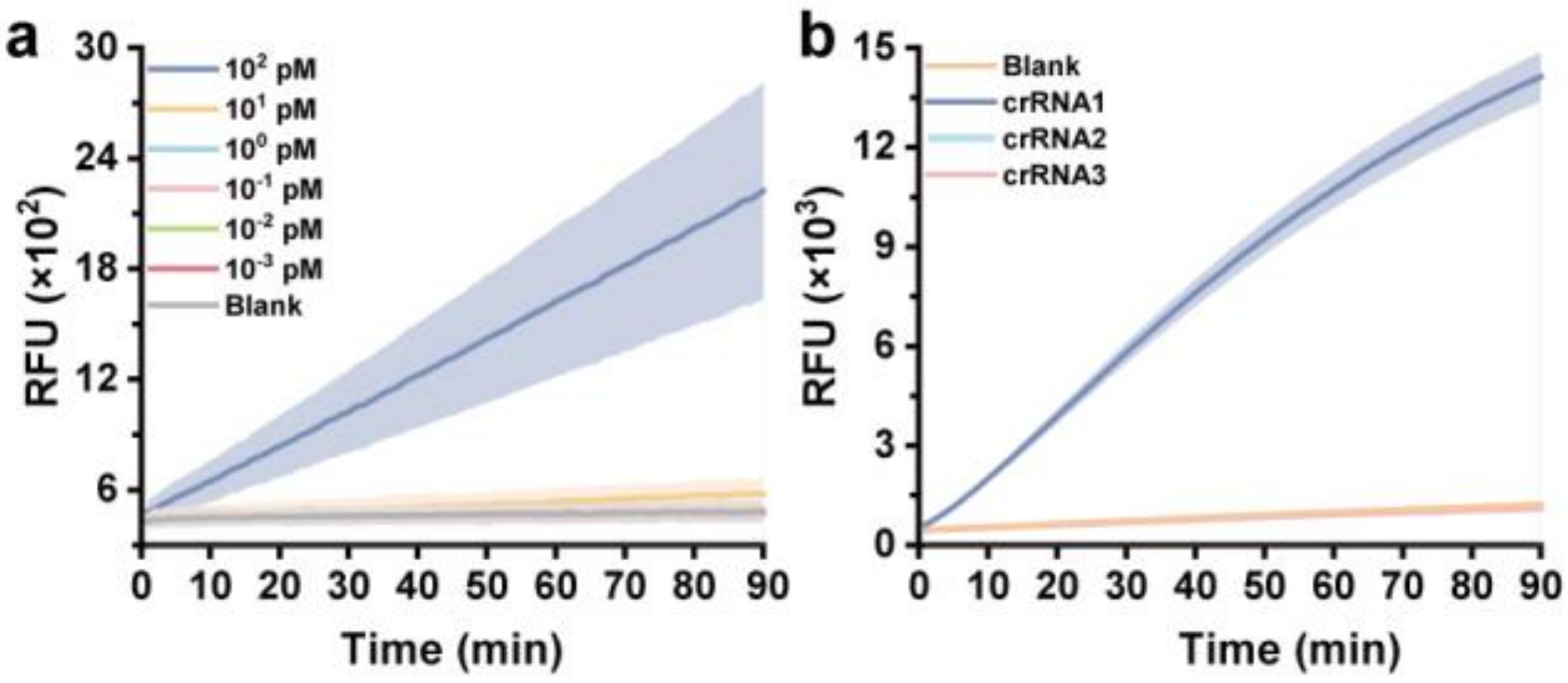

Figure S28. Real-time fluorescence kinetics of the target DNA (from 100 pM to 1 fM) using the single Cas12a system (a) and different crRNAs using the CALCas system (b). Data are expressed as mean ± SD (n = 3).

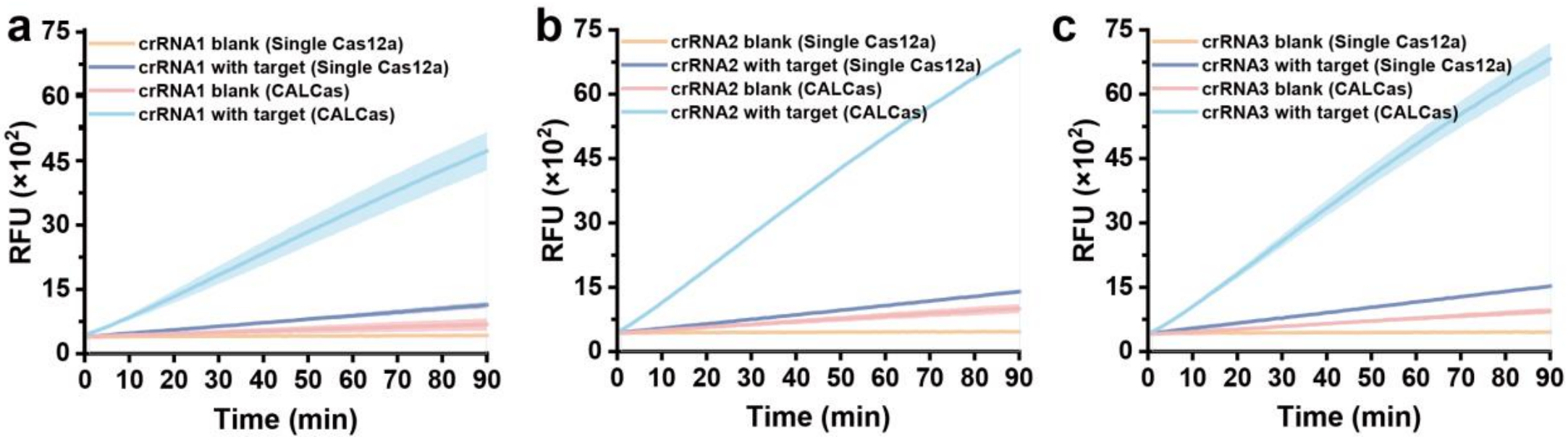

Figure S29. ΔRFU and real-time fluorescence kinetics of the single Cas12a and CALCas systems for detecting different targets. Data are expressed as mean ± SD (n = 3).

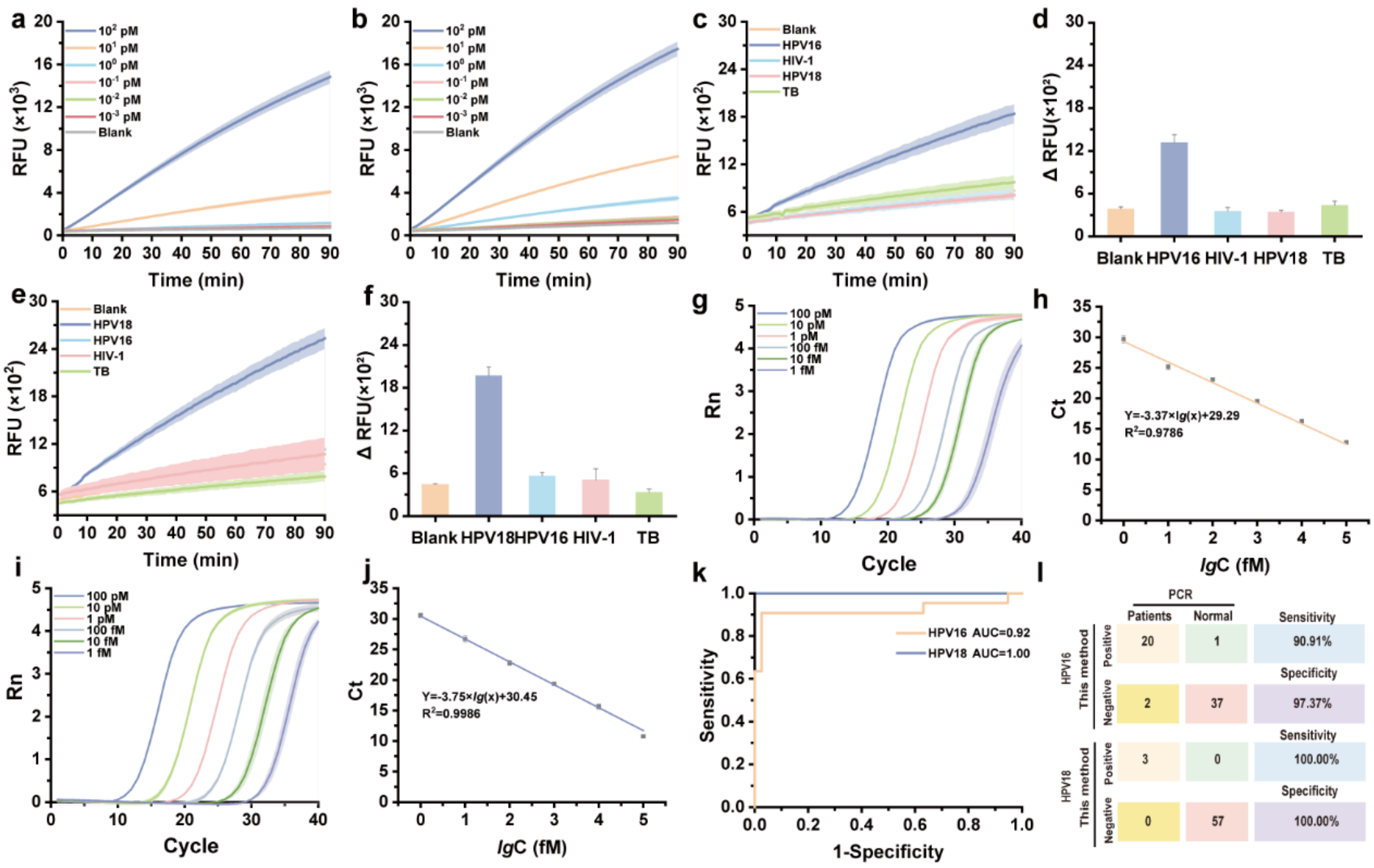

Figure S30. (a, b) Monitoring real-time fluorescence of the CALCas system for detection of HPV16/18 at varying concentrations. (c-f) Real-time fluorescence and ΔRFU for specific detection of HPV16/18. (g-j) Real-time fluorescence signals of HPV16/18 plasmids during PCR amplification, and the linear relationship between Ct value and the logarithm of target concentration. (k) ROC analysis for HPV16/18. (l) Sensitivity and specificity for HPV16/18 detection are provided in the table. Data are expressed as mean ± SD (n = 3).

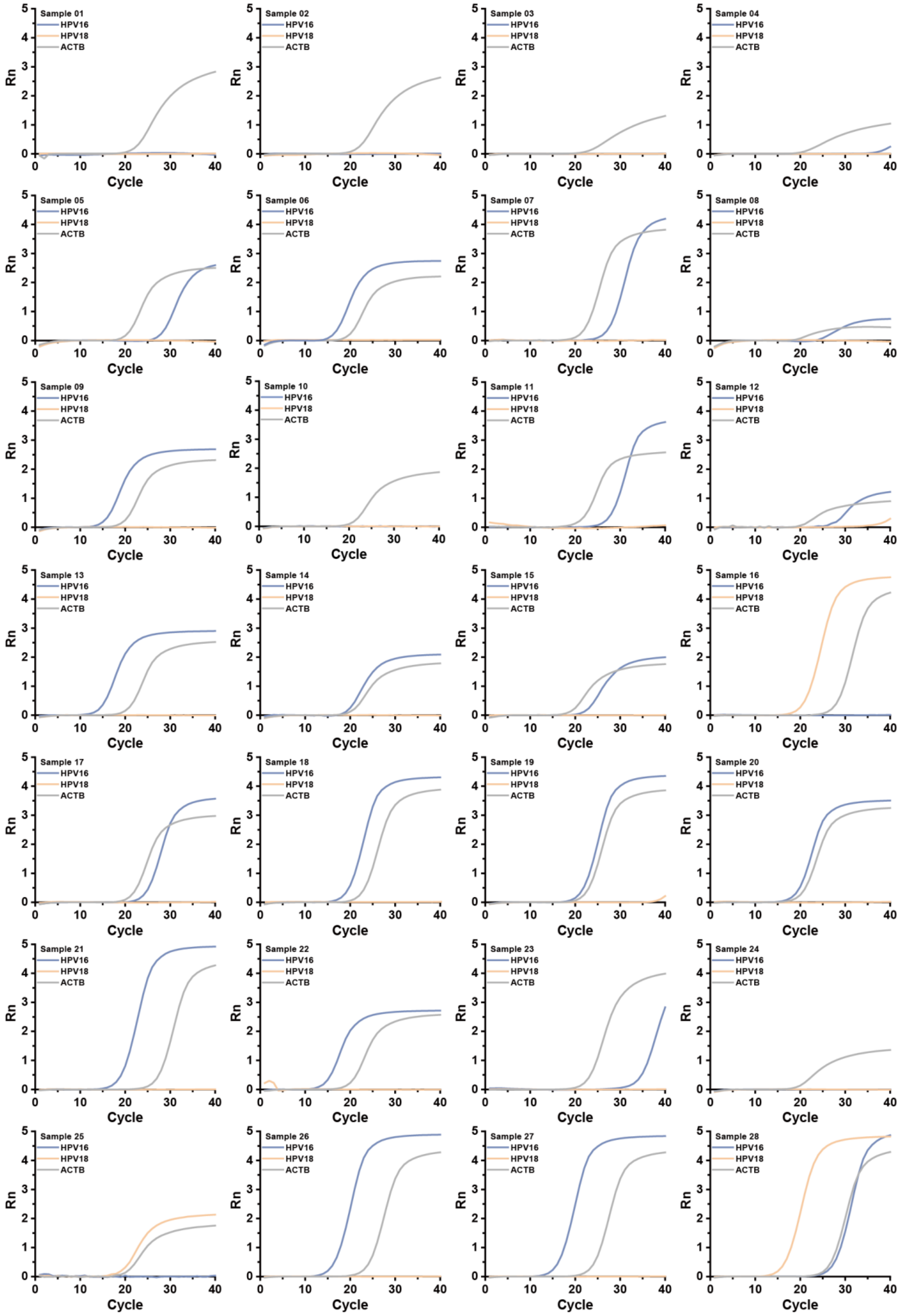

Figure S31. PCR analysis of 28 clinical samples (Samples 1-28).

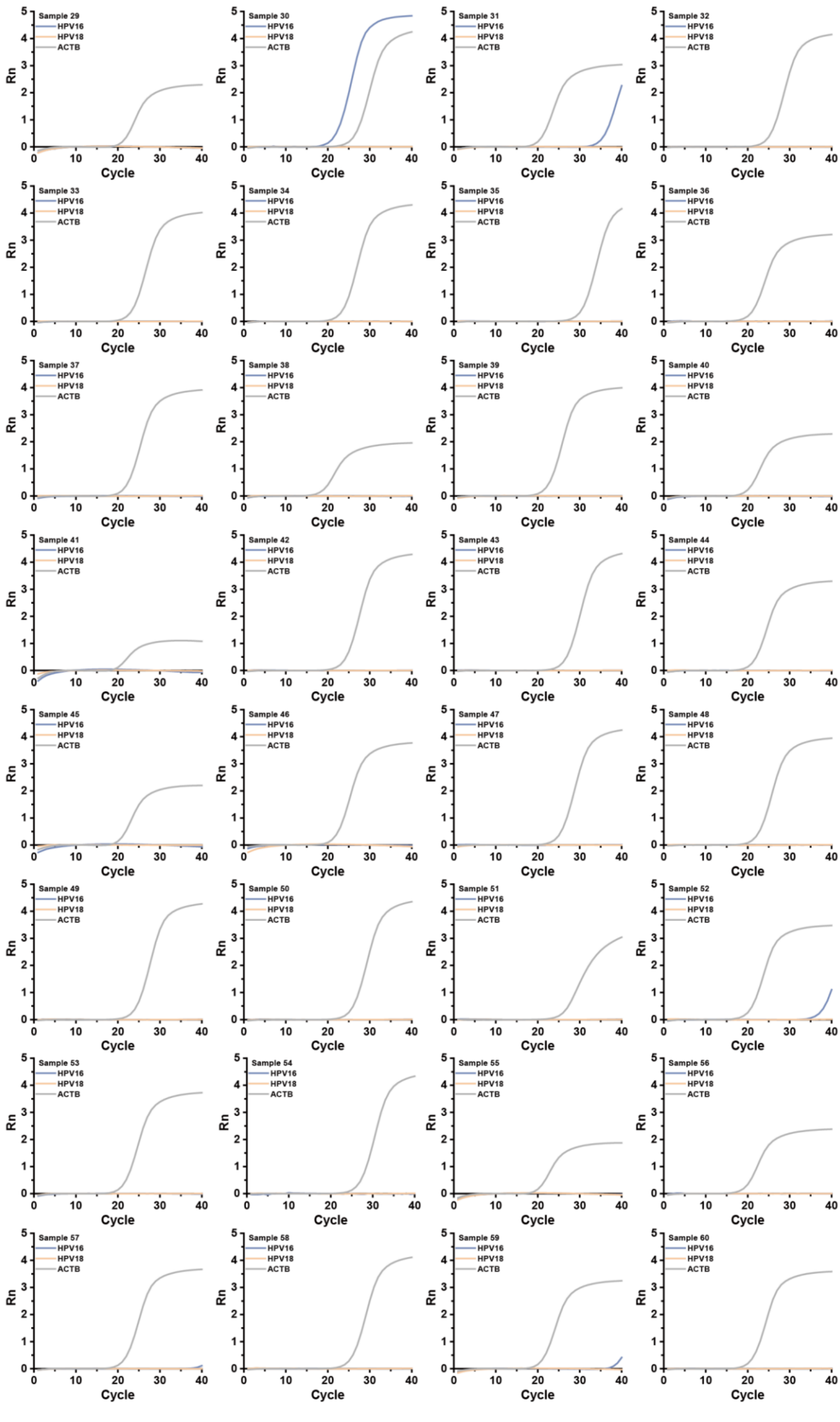

Figure S32. PCR analysis of 32 clinical samples (Samples 29- 60).

Reference

1. S. Yuan, H. C. S. Chan, and Z. Hu, "Using PYMOL as a Platform for Computational Drug Design," WIREs Computational Molecular Science 7, no. 2 (2017): e1298, https://doi.org/10.1002/wcms.1298.

2. M. G. Krokidis, D. E. Koumadorakis, K. Lazaros, et al., "AlphaFold3: An Overview of Applications and Performance Insights," International Journal of Molecular Sciences 26, no. 8 (2025): 3671, https://doi.org/10.3390/ijms26083671.
