# Supplementary material for: Scaffold RNA Reengineering for a Leakage‐Suppressed CRISPR/Cas12a Cascade System Toward Precise Nucleic Acid Diagnostics
Source: Adv Sci (Weinh). 2026 Oct 22:e78246. Online ahead of print. doi: 10.1002/advs.78246 (PMC13690970; doi:10.1002/advs.78246)
Supplement: Supplementary file 2 [file ADVS-9999-e78246-s002.pdf]

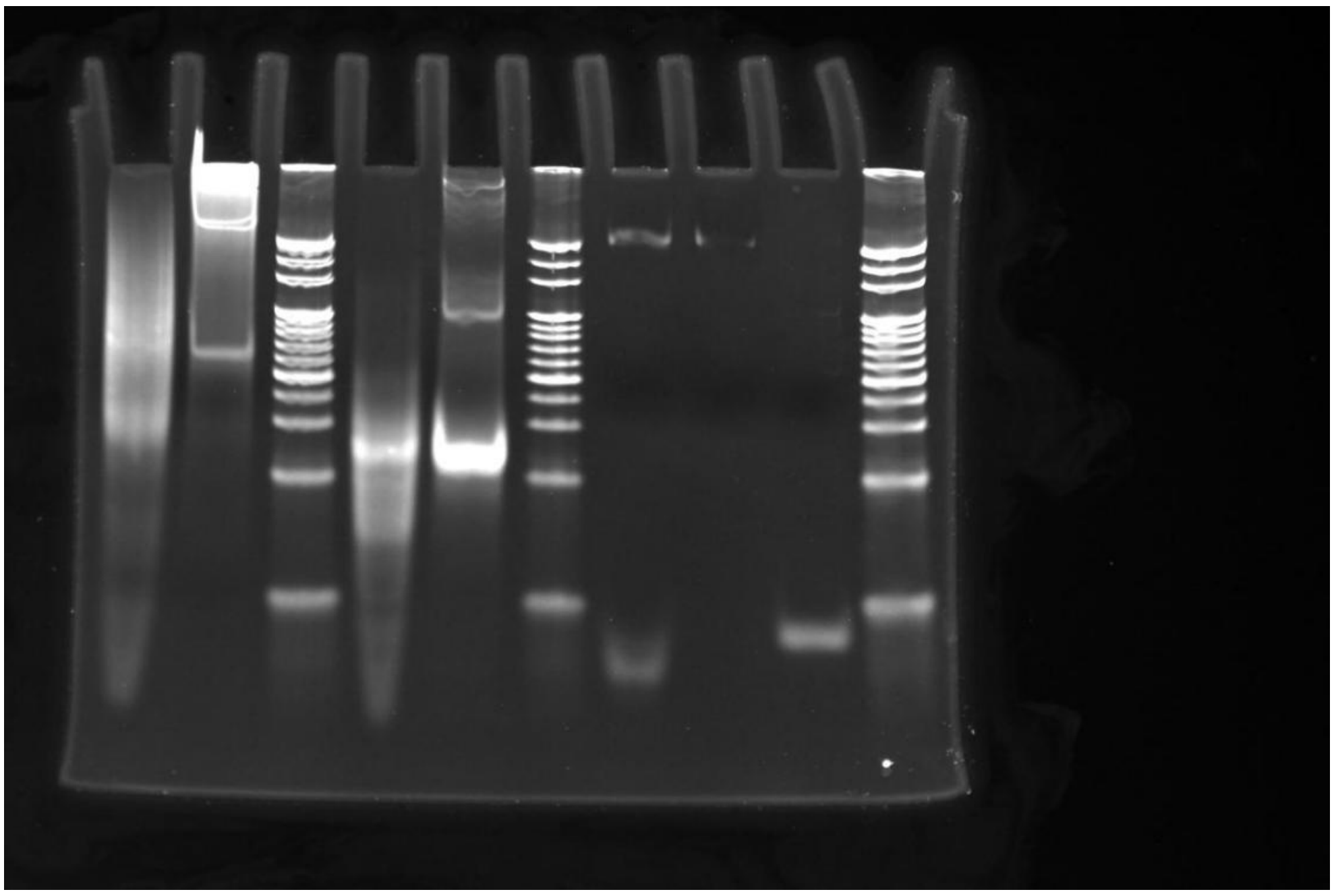

Figure G1. Uncropped gel images corresponding to Figure S5g and Figure S5h. The four rightmost lanes are from a different experiment not related to this study and should be excluded from analysis.

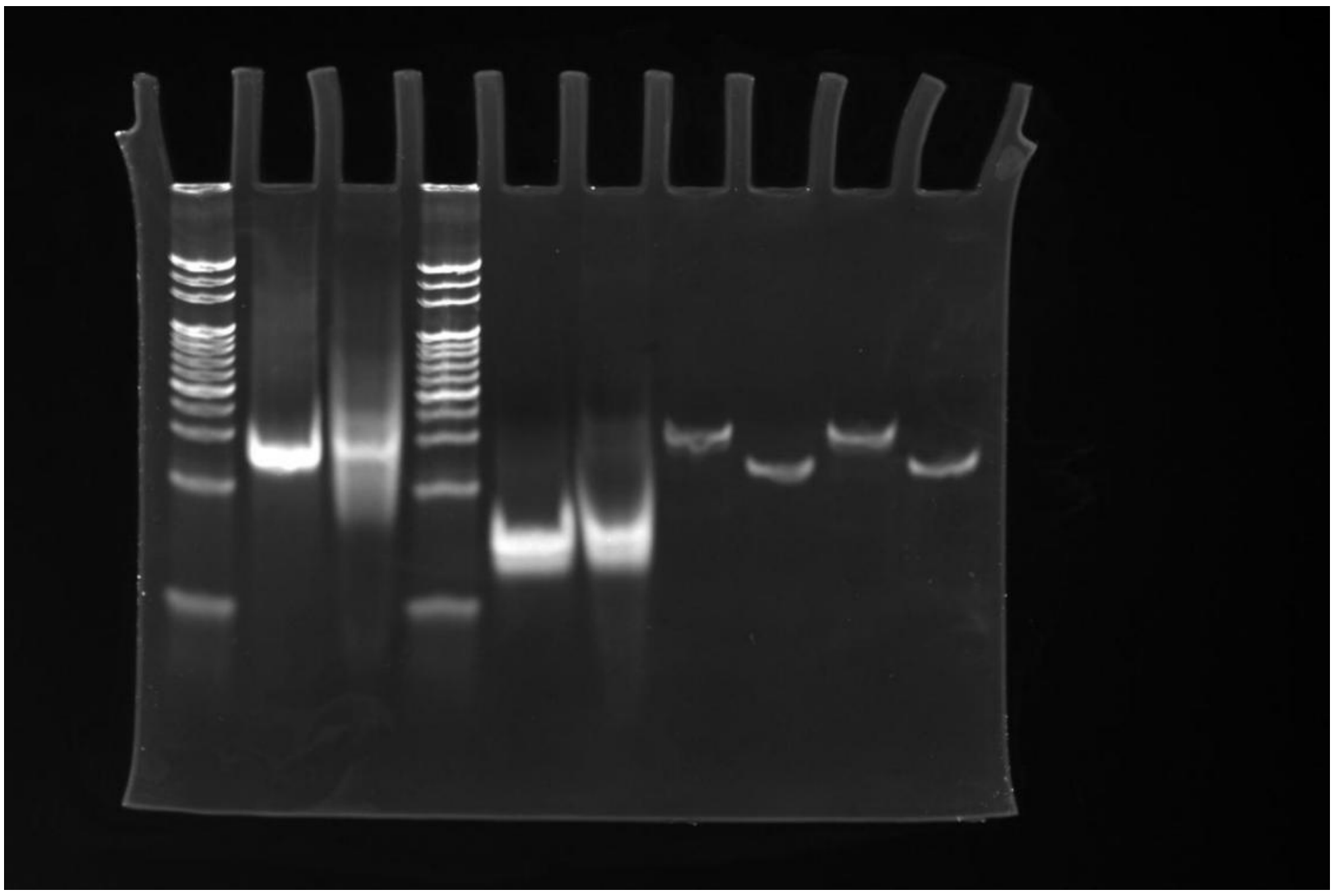

Figure G2. Uncropped gel images corresponding to Figure S5q and Figure S5r. The four rightmost lanes are from a different experiment not related to this study and should be excluded from analysis.

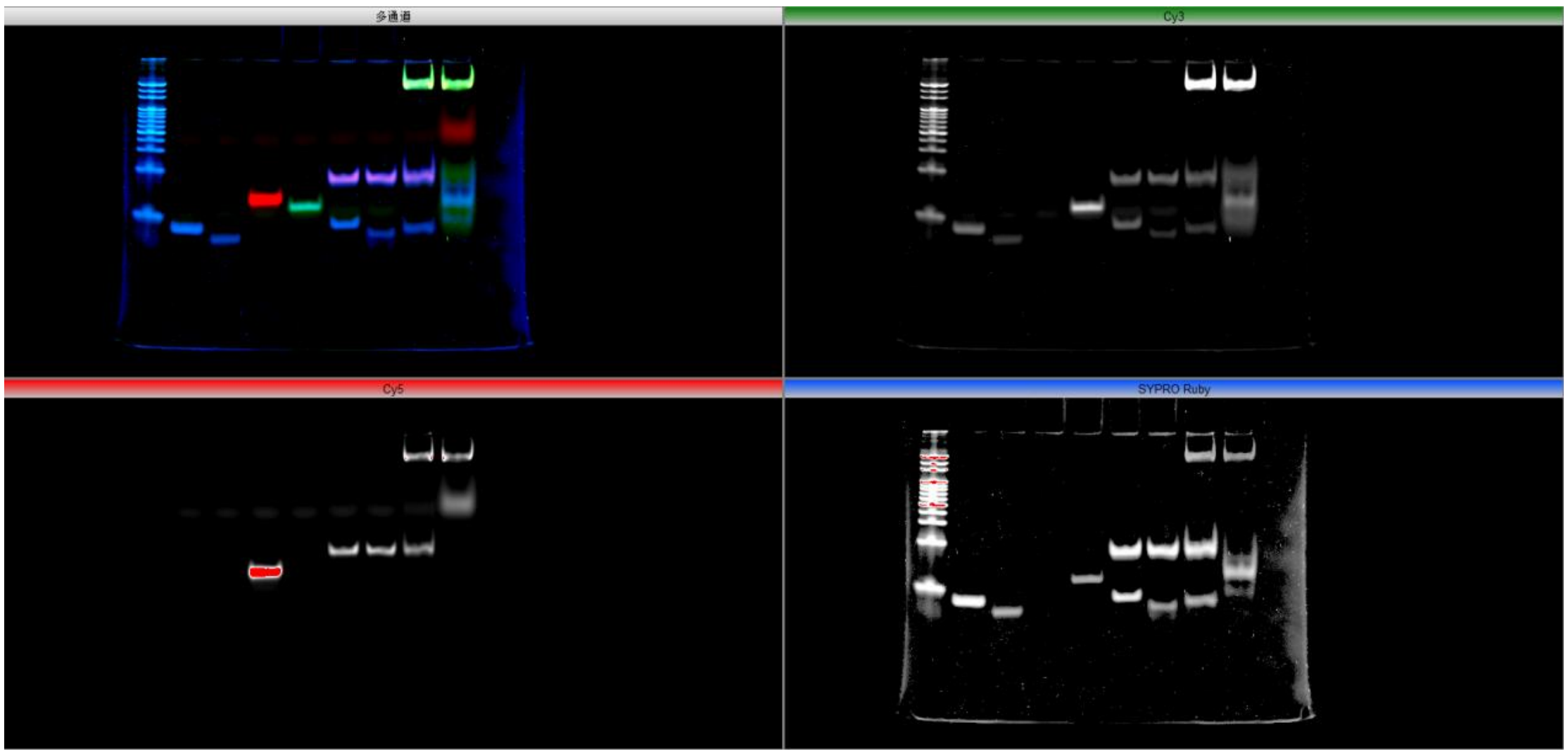

Figure G3. Uncropped gel images corresponding to Figure 2d.

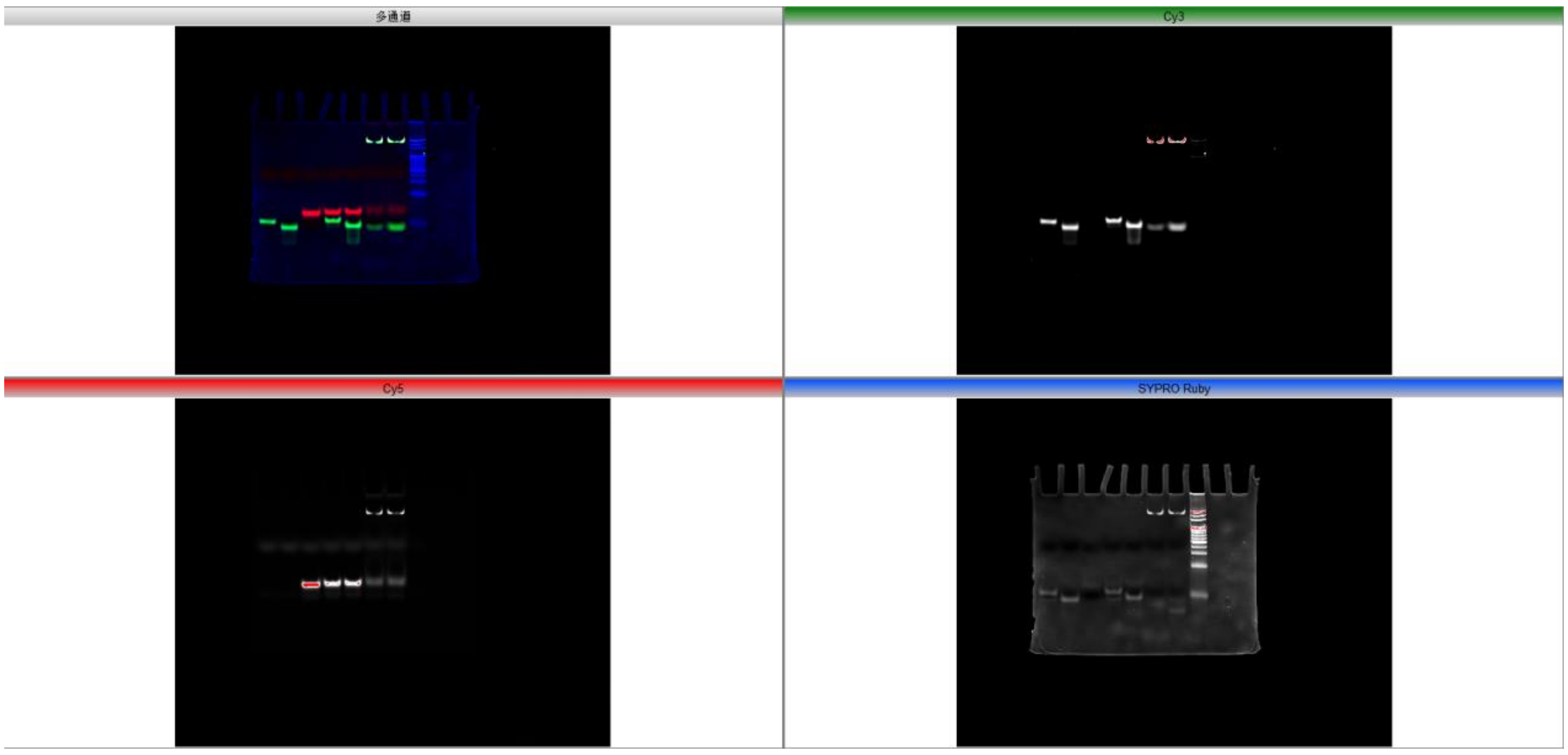

Figure G4. Uncropped gel images corresponding to Figure S7l.

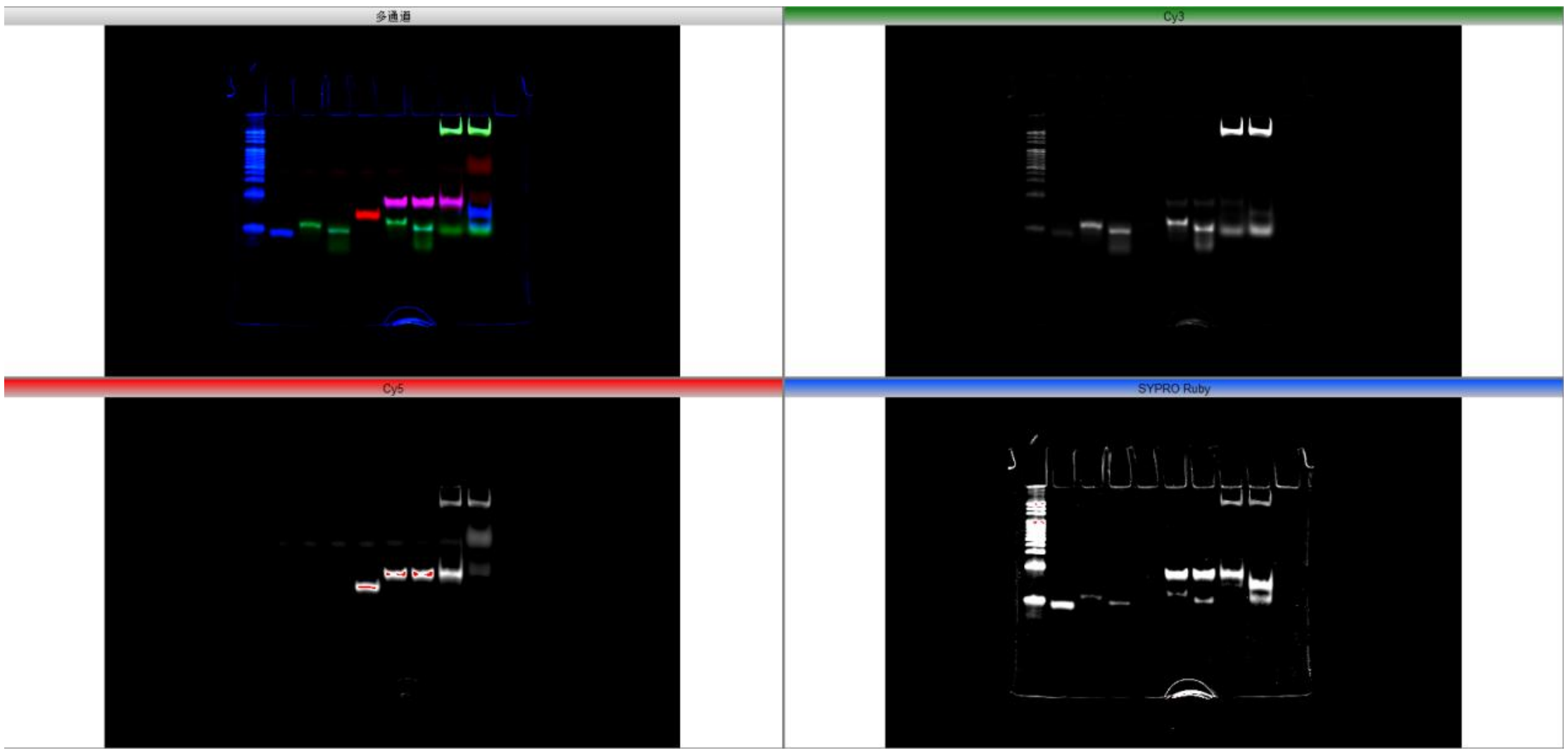

Figure G5. Uncropped gel images corresponding to Figure S7m.

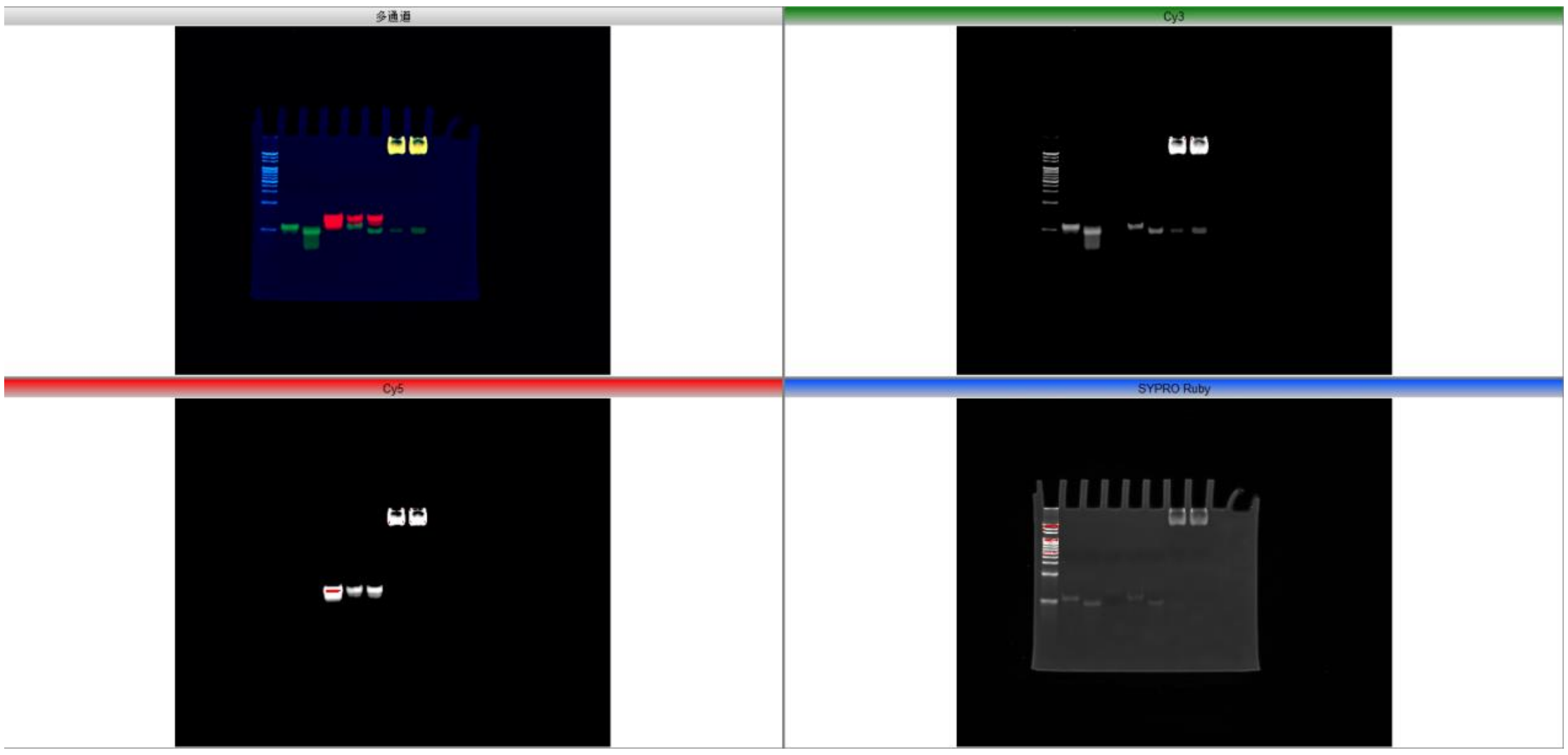

Figure G6. Uncropped gel images corresponding to Figure S8l.

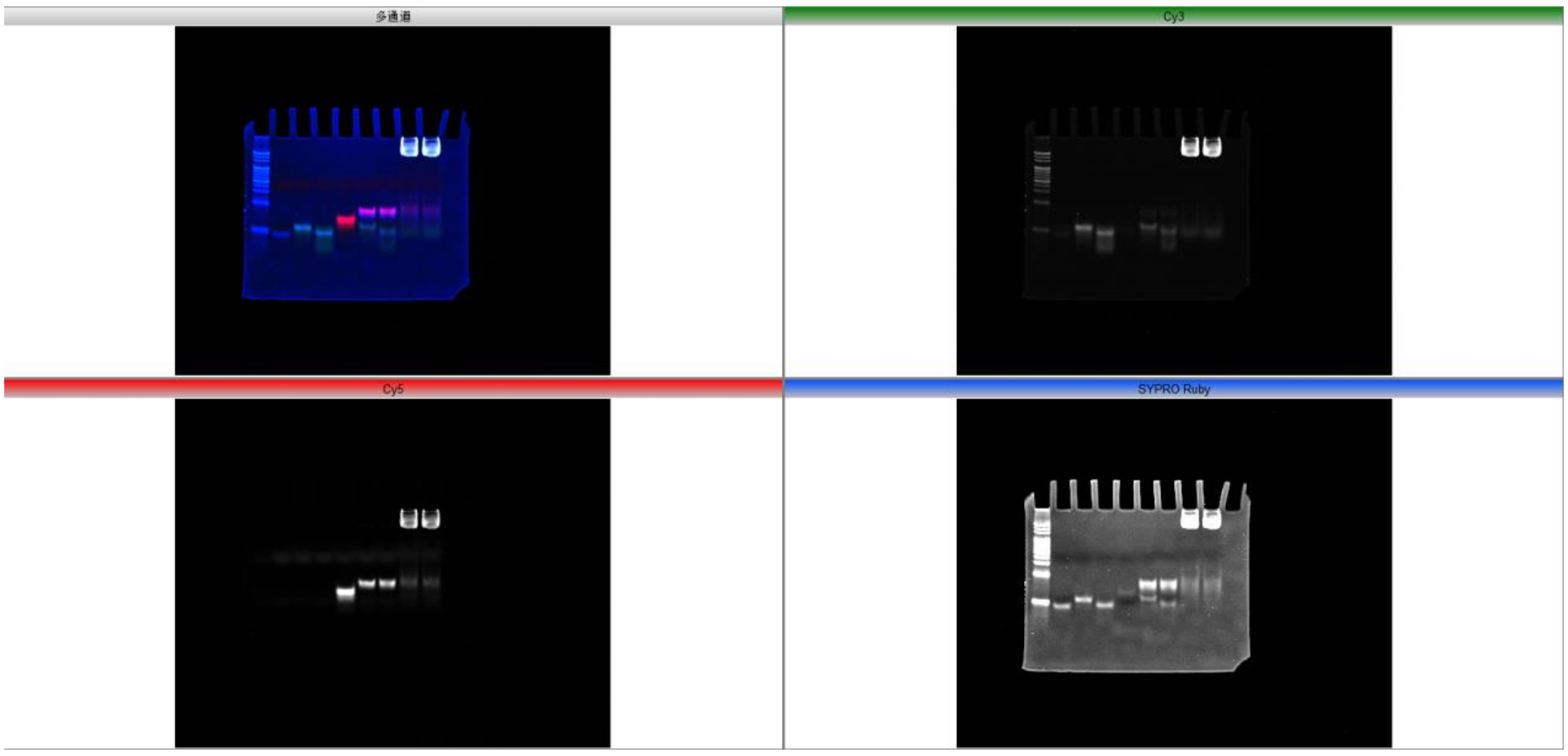

Figure G7. Uncropped gel images corresponding to Figure S8m.

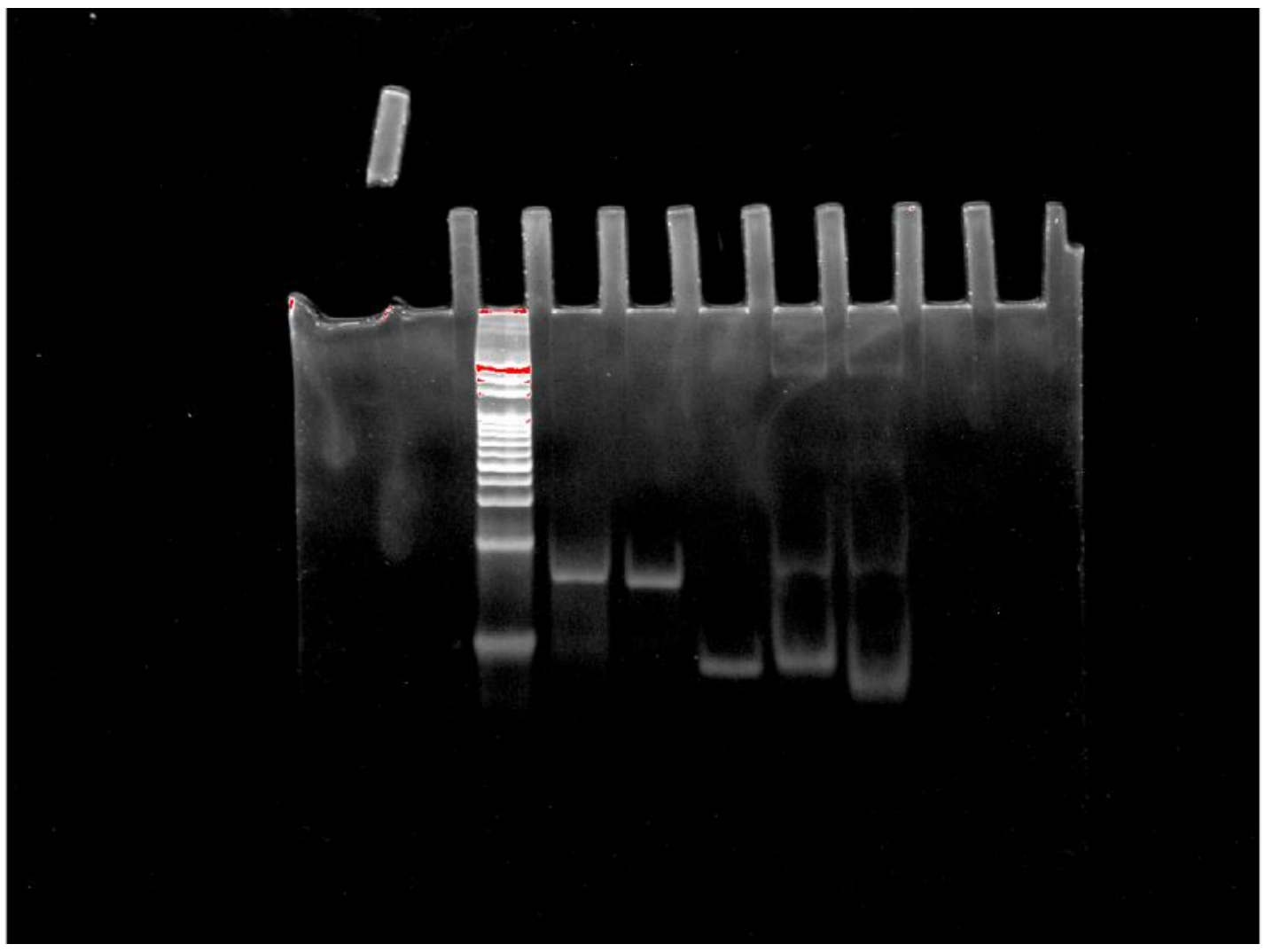

Figure G8. Uncropped gel images corresponding to Figure 4e.

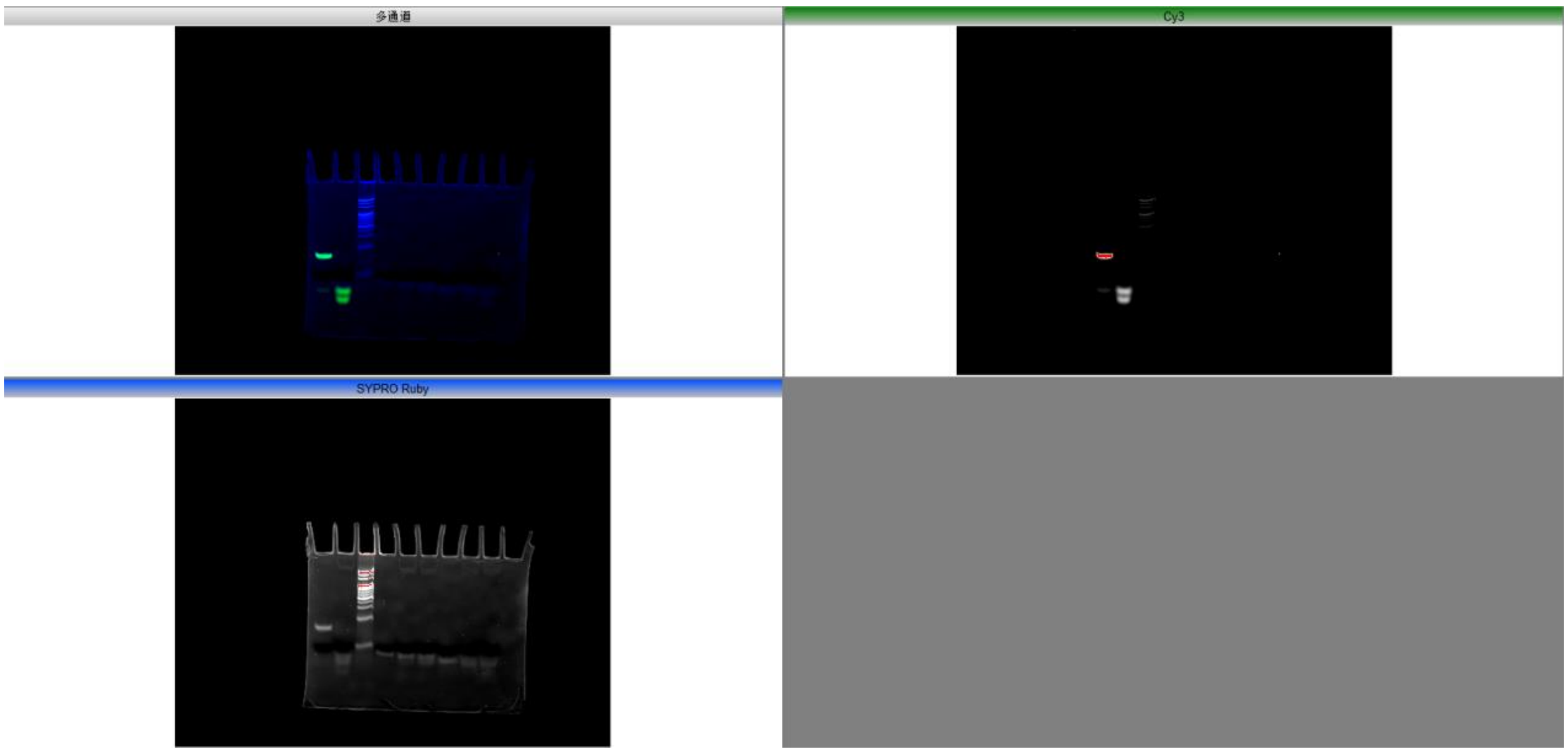

Figure G9. Uncropped gel images corresponding to Figure S15a.The six rightmost lanes are from a different experiment not about this study and should be excluded from analysis.

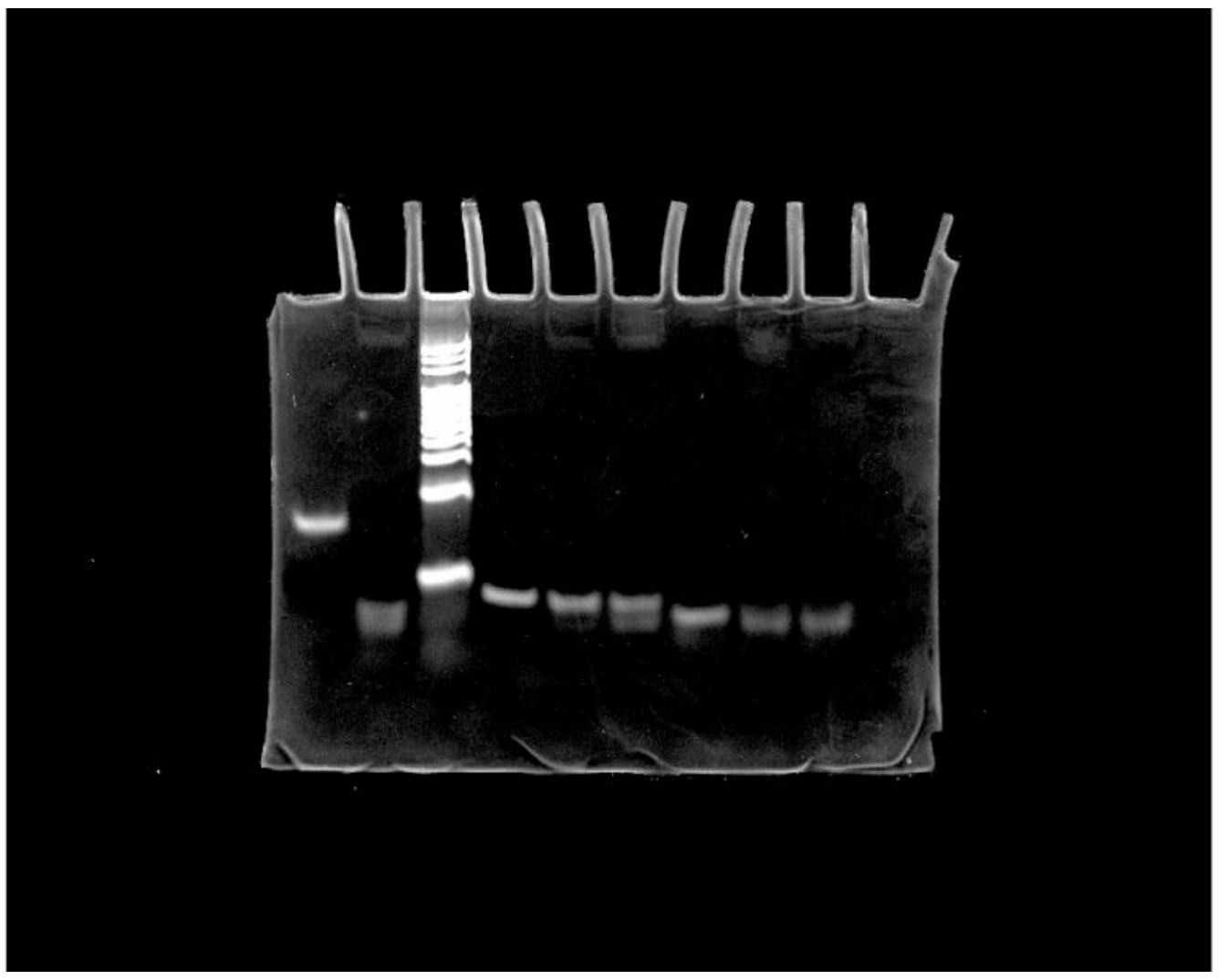

Figure G10. Uncropped gel images corresponding to Figure S16.The two leftmost lanes are from a different experiment not pertaining to this study and should be excluded from analysis.
